# Distinct Group B *Streptococcus* Sequence and Capsule Types Differentially Impact Macrophage Stress and Inflammatory Signaling Responses

**DOI:** 10.1128/IAI.00647-20

**Published:** 2021-04-16

**Authors:** Rebecca A. Flaherty, David M. Aronoff, Jennifer A. Gaddy, Margaret G. Petroff, Shannon D. Manning

**Affiliations:** a Department of Microbiology and Molecular Genetics, Michigan State University, East Lansing, Michigan, USA; b Department of Medicine, Division of Infectious Disease, Vanderbilt University Medical Center, Nashville, Tennessee, USA; c Department of Pathology, Microbiology, and Immunology, Vanderbilt University Medical Center, Nashville, Tennessee, USA; d Department of Obstetrics and Gynecology, Vanderbilt University Medical Center, Nashville, Tennessee, USA; e Tennessee Valley Healthcare System, Department of Veterans Affairs, Nashville, Tennessee, USA; f Pathobiology and Diagnostic Investigation, Michigan State University, East Lansing, Michigan, USA; g Cell and Molecular Biology Program, Michigan State University, East Lansing, Michigan, USA; Albert Einstein College of Medicine

**Keywords:** group B *Streptococcus*, *Streptococcus agalactiae*, macrophages, inflammatory response, mitogen-activated protein kinase, cytotoxicity, cell signaling, host response, host-pathogen interactions

## Abstract

Group B *Streptococcus* (GBS) is an opportunistic bacterial pathogen that can contribute to the induction of preterm birth in colonized pregnant women and to severe neonatal disease. Many questions regarding the mechanisms that drive GBS-associated pathogenesis remain unanswered, and it is not yet clear why virulence has been observed to vary so extensively across GBS strains. Previously, we demonstrated that GBS strains of different sequence types (STs) and capsule (CPS) types induce different cytokine profiles in infected THP-1 macrophage-like cells. Here, we expanded on these studies by utilizing the same set of genetically diverse GBS isolates to assess ST and CPS-specific differences in upstream cell death and inflammatory signaling pathways. Our results demonstrate that particularly virulent STs and CPS types, such as the ST-17 and CPS III groups, induce enhanced Jun-N-terminal protein kinase (JNK) and NF-κB pathway activation following GBS infection of macrophages compared with other ST or CPS groups. Additionally, we found that ST-17, CPS III, and CPS V GBS strains induce the greatest levels of macrophage cell death during infection and exhibit a more pronounced ability to be internalized and to survive in macrophages following phagocytosis. These data provide further support for the hypothesis that variable host innate immune responses to GBS, which significantly impact pathogenesis, stem in part from genotypic and phenotypic differences among GBS isolates. These and similar studies may inform the development of improved diagnostic, preventive, or therapeutic strategies targeting invasive GBS infections.

## Abstract (precis)

Group B *Streptococcus* (GBS) is an opportunistic bacterial pathogen that can contribute to the induction of preterm birth in colonized pregnant women and to severe neonatal disease. Many questions regarding the mechanisms that drive GBS-associated pathogenesis remain unanswered, and it is not yet clear why virulence has been observed to vary so extensively across GBS strains.

## INTRODUCTION

Streptococcus agalactiae, also known as group B *Streptococcus* (GBS), commonly colonizes the genitourinary and gastrointestinal tracts of approximately one-third of healthy individuals (1). Although GBS colonization is generally asymptomatic in otherwise healthy adults, it may lead to severe pregnancy or neonatal complications, such as preterm birth, stillbirth, and neonatal sepsis and meningitis (1). Maternal GBS colonization is the foremost risk factor for preterm birth and neonatal disease. Pregnancy complications in colonized mothers may occur when GBS ascends the mother’s vaginal tract, crosses the extraplacental membranes, and initiates an infection while the baby is still developing *in utero* (2). Alternatively, babies may be exposed during the birthing process by inhaling vaginal fluid containing GBS (3). Infected newborns may develop early-onset disease (EOD) within the first week following delivery, which usually presents as pneumonia and sepsis, or late-onset disease (LOD), which occurs from 1 week to 3 months after birth and generally presents as meningitis and sepsis (1). The risk of EOD can be reduced by administering antibiotics prophylactically to colonized mothers during delivery, but no treatment is currently available to mitigate the risk of GBS-related pregnancy complications or LOD in neonates (1).

To address the existing gaps in prevention, diagnostics, and treatment of GBS-related conditions, a more comprehensive understanding of the host immune response to GBS infection is needed. Prior studies have demonstrated that a dysregulated inflammatory response can promote severe pregnancy and postdelivery outcomes, including extraplacental membrane weakening and neonatal sepsis (3, 4). The role of macrophages in responding to both neonatal and *in utero* infections is of particular relevance. Because neonates initially have an immature adaptive immune system, they rely heavily on the innate immune system, in which macrophages play a central role, to combat bacterial infections (5). Additionally, macrophages are common at the maternal-fetal interface where they promote maternal tolerance to the developing fetus and destroy pathogens that cross the extraplacental membranes (6).

We previously tested the role of macrophages in the inflammatory response to GBS using multiplexed cytokine arrays and enzyme-linked immunosorbent assays (ELISAs) to compare cytokine production in THP-1 macrophage-like cells in response to different sequence types (STs) and capsule types (serotypes) of GBS (7). There is extensive phenotypic and genotypic variation between GBS strains, and this diversity is believed to be a key factor in determining the severity of GBS disease (8–11). For example, we and others have shown that ST-17 GBS strains are more common in severely infected newborns than in colonized pregnant mothers (12–15). Consistent with these observations, the aforementioned analysis of macrophage responses to 15 distinct clinical strains revealed that certain inflammatory cytokines were universally induced in response to all strains, while other responses were unique to specific strains, genotypes, or serotypes.

Here, we expanded on these studies by utilizing the same set of genetically diverse GBS strains to assess differences in upstream stress and inflammatory signaling pathways using protein arrays and quantitative biochemical analyses. Our results demonstrate ST and CPS-specific differences in the activation of the Jun-N-terminal protein kinase (JNK) and NF-κB pathways. We also observed variation in the level of macrophage death during infection and differences in macrophage internalization and survival following phagocytosis across GBS strains. These data provide further support for the hypothesis that variable host innate immune responses to GBS, which significantly impact pathogenesis, stem in part from genotype and phenotype-specific differences in GBS isolates. These and similar studies may inform the development of improved diagnostic or therapeutic strategies targeting invasive GBS infections.

## RESULTS

### Antibody-based protein array reveals general and strain-specific responses to GBS infection in THP-1 macrophages.

To evaluate global changes in protein expression or activation state in macrophages in response to GBS infection, a protein array analyzing 386 different human proteins was utilized following exposure of THP-1 macrophages to various sequence types of GBS for 1 h. This array identified many proteins with significantly altered total or phosphorylated levels relative to mock infection controls (see Table S1 in the supplemental material). Results from the five strains were initially pooled to identify the most common macrophage responses to GBS regardless of the strain type. Averaging the responses for each protein target identified 16 proteins with a 2-fold or greater fold change increase (f.c.i.) in phosphorylated or total levels. These proteins include cofilin 1 (21.3 f.c.i.), STAT1 (4.8 f.c.i.), JNK2 (3.7 f.c.i.), p38a MAPK (3.7 f.c.i.), KIT (3.3 f.c.i.), InsR (3.0 f.c.i.), ACTA1 (3.0 f.c.i.), STAT3 (2.9 f.c.i.), PKCl/l (2.9 f.c.i.), Connexin 43 (2.8 f.c.i.), KSR (2.6 f.c.i.), p70 S6K (2.6 f.c.i.), JAK1 (2.6 f.c.i.), STAT5A (2.5 f.c.i.), STAT5B (2.4 f.c.i.), and RelB (2.2 f.c.i.) (Table S2). When the protein targets were grouped by function, the most highly affected signaling pathways were those responsible for the regulation of inflammation and the immune response, cell survival and metabolism, rearrangement of the cytoskeleton and cell-cell contacts, hormone signaling, and angiogenesis (Table 1). Notably, some THP-1 signaling responses were unique to specific GBS strains; approximately 50 proteins with a fold change of two or more were identified for at least one of the five GBS strains. These proteins were ACTA1, arrestin b, catenin b, cofilin 1, connexin 43, COT, DNAPK, eIF4G, EphA3, FGRF2, FOS, HCA59, histone H2A.X, histone H2B, HSP90AB1, Huntingtin, IGF1R, IKKa, InsR, ITSN2, JAK1, JNK2, Jun, Kit, KSR, MEK3/6, MSK1, MST1, mTOR, NEK2, p38 MAPK, p53, p70 S6K, PAK1, PAK2, PKC, Rb, RelB, ROCK2, RPS6, SMC1, SRC, STAT1, STAT2, STAT3, STAT5, SYK, Tau, and TEC (Table S1).

**TABLE 1:**
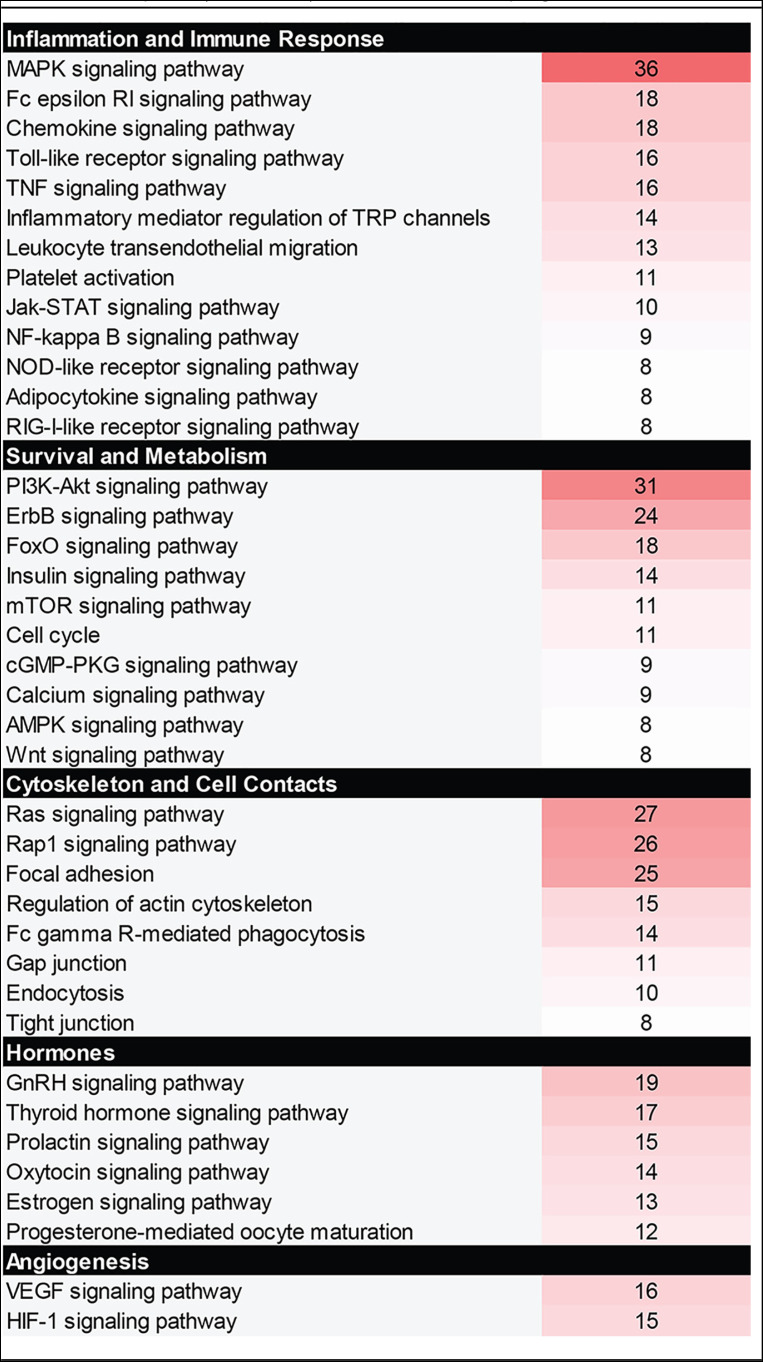
Cellular pathways affected by GBS infection in macrophages^*a*^

a Color coding corresponds to the number of array hits for each pathway with the darker shades having more hits.

### GBS induces mitogen-activated protein kinase (MAPK) signaling in macrophages.

The signaling pathway with the most significantly altered protein targets in response to GBS infection was the MAPK pathway (Table 1). This pathway is critically important for inducing stress responses to various stimuli, the modulation of inflammatory signaling, and the regulation of cell death signaling cascades (16). Significant changes in protein activity and level in response to GBS infection were particularly evident in proteins involved in the stress-activated p38 and Jun-N-terminal kinase (JNK) MAPK pathways (Fig. S1). For example, p38 was increased up to 3.9-fold in THP-1 cells compared to mock infection in response to some GBS strains, while JNK was increased up to 4.5-fold. Consequently, key members of these pathways were selected for follow-up analyses with the five strains included in the array.

**FIG 1:**
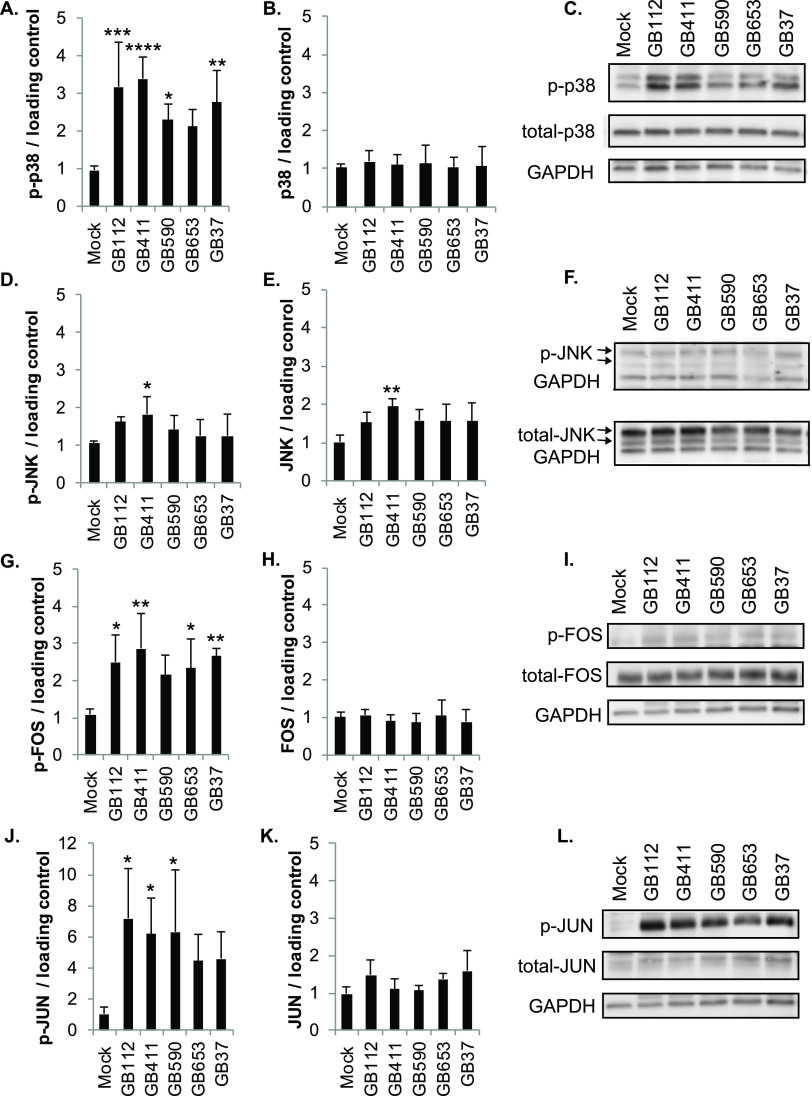
GBS induces strain-specific activation of stress-responsive MAPKs. THP-1 macrophages were infected with GBS at a multiplicity of infection (MOI) of 10 for 1 h, washed, and treated with antibiotics for an additional hour prior to lysate collection. (A to L) Lysates were assessed for phosphorylated (active) or total protein levels of p38 (A to C), JNK (D to F), FOS (G to I), and JUN (J to L), and densitometry was used to compare differences between infection conditions. Densitometry values represent pooled results from at least three independent biological replicates, and error bars represent standard deviations of the mean. Significance was determined by ANOVA (*P* values: phospho-p38, 0.0001; total p38, 0.2608; phospho-JNK, 0.119, total JNK, 0.0316; phospho-FOS, 0.0136; total FOS, 0.818; phospho-JUN, 0.0347; total JUN, 0.0663) with *post hoc* Dunnett’s testing to compare each infection condition to the mock infection (*, *P* = 0.01 to 0.05; **, *P* = 0.001 to 0.01; ***, *P* = 0.0001 to 0.001; ****, *P* < 0.0001). Representative Western blots from one biological replicate with its corresponding loading control (GAPDH) are shown (C, F, I, and L). Equal amounts of the same protein lysate preparations were loaded onto the gels for each protein.

Analysis by Western blotting and densitometry indicated significantly increased phosphorylated (active) p38 levels compared to mock infection in response to infection with four of the five GBS strains; phospho-p38 levels were consistently higher in response to the two ST-17 and capsule type III strains (GB112 and GB411) (Fig. 1A and C). No significant difference was observed in total p38 levels for any of the GBS strains (Fig. 1B and C), while a modest increase in both phosphorylated (active) and total JNK was observed in response to the invasive ST-17 strain, GB411. No significant differences in active or total JNK, however, were observed in response to the remaining strains (Fig. 1D to F).

Enhanced phosphorylated levels in the downstream stress-activated MAPK pathway targets FOS, and JUN supported GBS-induced activation of the p38 and JNK MAPK pathways. We observed significant increases in phospho-FOS in response to all strains except GB590 (Fig. 1G and I) and in phospho-JUN for three of the five strains (GB112, GB411, and GB590) (Fig. 1J and L). Nonetheless, these proteins trended toward increased active levels for all strains tested. A significant increase in the total levels of these downstream targets, however, was not observed for any of the strains (Fig. 1H, I, K and L).

To determine whether these strain-specific differences in the activation of the p38 and JNK MAPK pathways were consistent with responses induced by GBS strains with the same STs and CPS types, an additional set of 10 diverse GBS clinical isolates was evaluated. Consistent with our earlier results, most of the additional strains tested induced significant increases in phospho-p38 levels (Fig. S2A and E). Similarly, only an invasive ST-17 strain (GB418) and an invasive ST-12 strain (GB910) exhibited significant increases in phospho-JNK levels, though several others trended toward enhanced phospho-JNK (Fig. S2C and E). Neither total p38 nor total JNK levels were significantly enhanced in response to any of the additional strains tested (Fig. S2B, D, and E).

**FIG 2:**
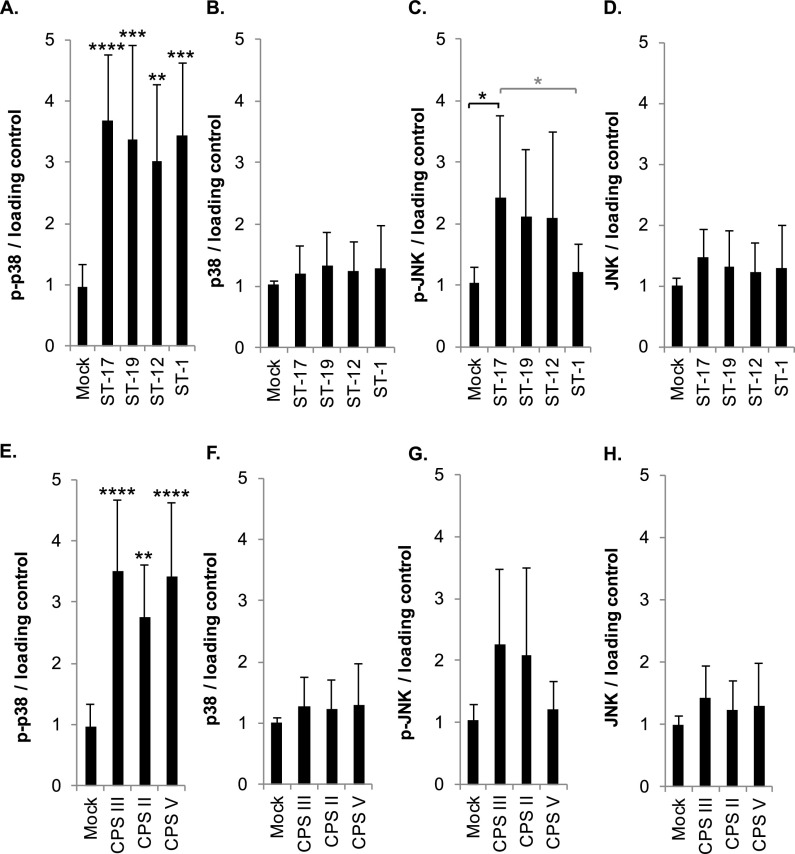
GBS sequence type (ST) and capsule (CPS) type influences MAPK activation. (A to H) Densitometry results from p38 and JNK Western blot analyses from all 15 strains were grouped according to sequence type (A to D) and capsule group (E to H). Within these groups, changes in phosphorylated or total protein levels were pooled and normalized to the mock infection results. Statistics were calculated using at least three independent biological replicates per strain; each ST or CPS group includes a minimum of three unique clinical isolates. ANOVAs (panel A, *P* < 0.0001; B, *P* = 0.6643; C, *P* = 0.0194; D, *P* = 0.2133; E, *P* < 0.0001; F, *P* = 0.6147; G, *P* = 0.0177; H, *P* = 0.2363), and *post hoc* Tukey’s tests (*, *P* = 0.01 to 0.05; **, *P* = 0.001 to 0.01; ***, *P* = 0.0001 to 0.001; ****, *P* < 0.0001) were used to compare the mean of each ST or CPS group to the mean of every other ST or CPS group. Comparisons to the mock infection are indicated with black asterisks, while comparisons to other ST or CPS groups are indicated with gray asterisks.

Next, we pooled the normalized densitometry results from the p38 and JNK Western blot analyses by ST and CPS type as a more robust method to compare MAPK pathway activation between these groups. Interestingly, phospho-p38 was significantly increased in response to all four STs (Fig. 2A) and all three CPS types (Fig. 2E) compared to mock infection. No significant differences were observed between STs or CPS types by analysis of variance (ANOVA) and *post hoc* Tukey’s tests for phospho-p38, though the greatest phospho-p38 levels were observed in the ST-17 and CPS III strains. Total p38 levels were not significantly altered in response to any of the ST or CPS groups (Fig. 2B and F); however, phospho-JNK was significantly increased for the ST-17 strains compared to the mock infection and ST-1 strains (Fig. 2C). When stratified by CPS, no significant differences were observed relative to the mock infection or other groups by Tukey’s test, though CPS III bordered on significance (*P* = 0.0564) relative to the mock infection (Fig. 2G). Total JNK levels were not significantly increased in response to any of the ST or CPS groups (Fig. 2D and H).

### GBS induces nuclear factor kappa B signaling in macrophages downstream of p38 and JNK MAPK activation.

**FIG 3:**
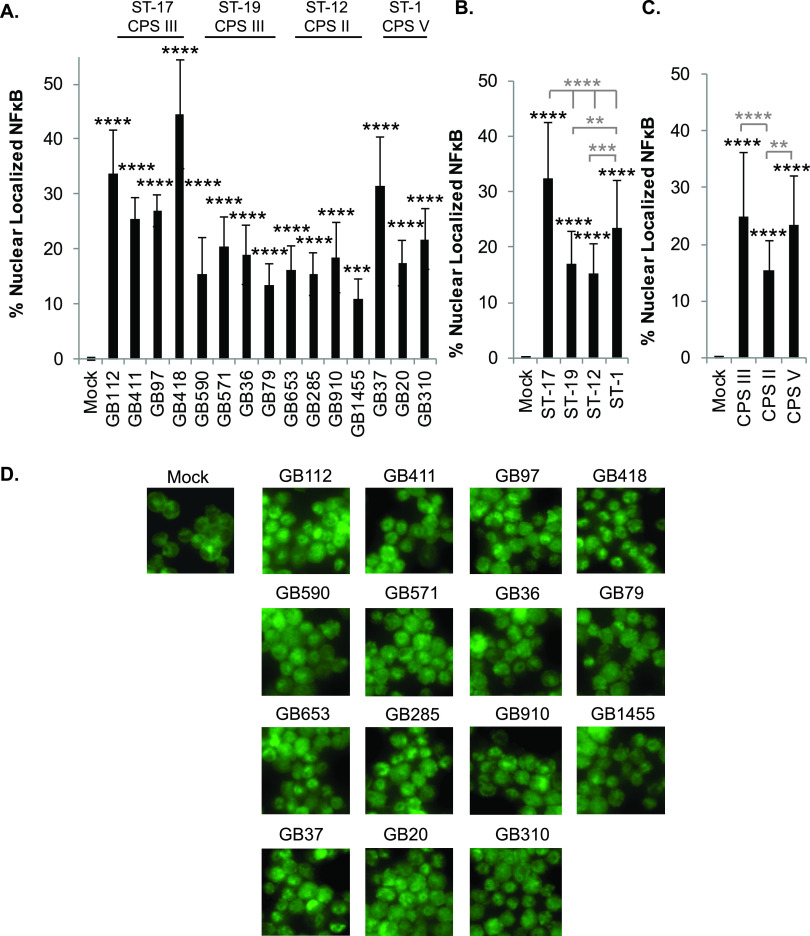
GBS-mediated NF-κB signaling in macrophages varies by sequence and capsule type. THP-1 macrophages were infected with GBS at a MOI of 10 for 1 h, washed, and treated with antibiotics for an additional hour prior to fixation, nuclear staining (DAPI), and detection of NF-κB p65 (Alexa Fluor 488) by immunofluorescence microscopy. The percentage of NF-κB nuclear localization compares the number of cells with positive nuclear localization (Alexa Fluor 488) to the total cell number in a given field (DAPI) using ImageJ. Results from three independent biological replicates were pooled for each of the 15 GBS strains analyzed. For each biological replicate, results were pooled from at least three separate fields to obtain a minimum of 2,500 cells per condition. (A) ANOVA (*P* < 0.0001) and a *post hoc* Dunnett’s test (*, *P* = 0.01 to 0.05; **, *P* = 0.001 to 0.01; ***, *P* = 0.0001 to 0.001; ****, *P* < 0.0001) were performed to compare the mean of the mock infection to the mean of each of the other infection conditions. (B and C) Results were then grouped according to ST (B) or CPS type (C), and ANOVAs (*P* values: B, *P* < 0.0001; C, *P* < 0.0001) and *post hoc* Tukey’s tests (*, *P* = 0.01 to 0.05; **, *P* = 0.001 to 0.01; ***, *P* = 0.0001 to 0.001; ****, *P* < 0.0001) were used to compare the mean of each to the mean of every other ST or CPS group. Comparisons to the mock infection are indicated with black asterisks, while comparisons to other ST or CPS groups are indicated with gray asterisks. (D) Representative microscopy images of NF-κB localization for each condition are shown.

The MAPK pathway initiates numerous downstream signaling cascades, including the nuclear factor kappa B (NF-κB) pathway. NF-κB is a central regulator of many proinflammatory cellular responses (17). Since the antibody array data indicated activation of several key NF-κB pathway proteins, this pathway was also selected for follow-up analyses with all 15 strains. To confirm pathway activation, immunofluorescence microscopy was used to examine nuclear localization of NF-κB in response to mock or GBS infection (Fig. 3 and Fig. S3 to S6). Notably, a significant increase in NF-κB activation was observed for all 15 strains compared to the mock infection controls (Fig. 3A and D and Fig. S3 to S6). After stratifying by ST, the ST-17 strains induced the greatest degree of NF-κB activation, which was significantly higher than each of the other three STs analyzed (Fig. 3B). The ST-1 strains also induced NF-κB nuclear localization at levels that were significantly higher than those of the ST-19 and ST-12 strains (Fig. 3B). When grouped by serotype, CPS III and CPS V induced similarly high levels of NF-κB activation, which were significantly higher than those observed for CPS II strains (Fig. 3C).

**FIG 4:**
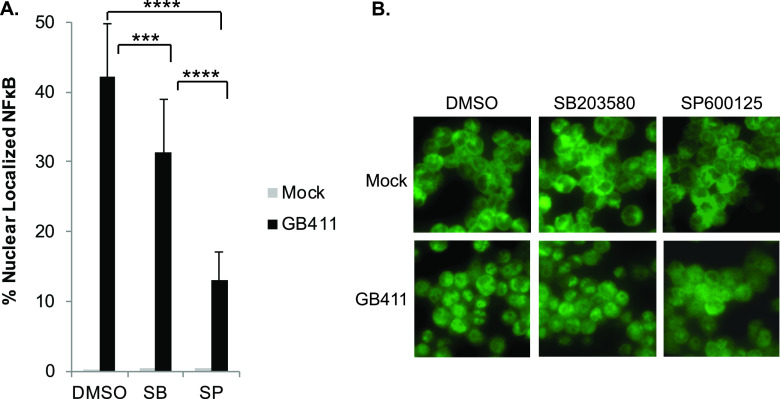
GBS-mediated NF-κB signaling in macrophages occurs downstream of MAPK activation. THP-1 macrophages were treated with a p38 inhibitor (SB203580, 10 μM), a JNK inhibitor (SP600125, 25 μM), or a vehicle control (DMSO) 1.25 h prior to infecting them with GBS at a MOI of 10 for 1 h. The cells were washed and treated with antibiotics for an other hour prior to fixation, nuclear staining (DAPI), and detection of NF-κB p65 (Alexa Fluor 488) by immunofluorescence microscopy. The percentage of NF-κB nuclear localization compares the number of cells with positive nuclear localization (Alexa Fluor 488) to the total cell number in a given field (DAPI) using ImageJ. Results from three independent biological replicates were pooled for each condition, each of which was obtained from at least three separate fields for a minimum of 2,200 cells per condition. (A) ANOVA (*P* < 0.0001) and a *post hoc* Tukey’s test (*, *P* = 0.01 to 0.05; **, *P* = 0.001 to 0.01; ***, *P* = 0.0001 to 0.001; ****, *P* < 0.0001) were used to compare the mean of each ST or CPS group to the mean of every other ST or CPS group. For simplicity, only statistical differences for the GB411 infection conditions are shown. There was no statistical difference between the mock infection vehicle control and mock infection inhibitor treatments, but GB411 conditions were all significantly increased compared to all mock infection conditions. (B) Representative microscopy images of NF-κB localization (Alexa Fluor 488) for each condition are shown.

To determine whether the increase in NF-κB activity was occurring downstream of p38 or JNK activation, we treated THP-1 cells with a p38 inhibitor (SB203580 [SB]), a JNK inhibitor (SP600125 [SP]), or a vehicle control (dimethyl sulfoxide [DMSO]) prior to GBS infection. We selected the invasive ST-17 CPS III GB411 strain for these experiments, as both p38 and JNK signaling were observed in THP-1 macrophages following infection with this strain. Importantly, p38 inhibition significantly reduced NF-κB activation by approximately 10%, while JNK inhibition significantly reduced NF-κB activation by nearly 30% (Fig. 4A and B).

### p38 and JNK MAPK influence GBS-mediated cytokine activation.

**FIG 5:**
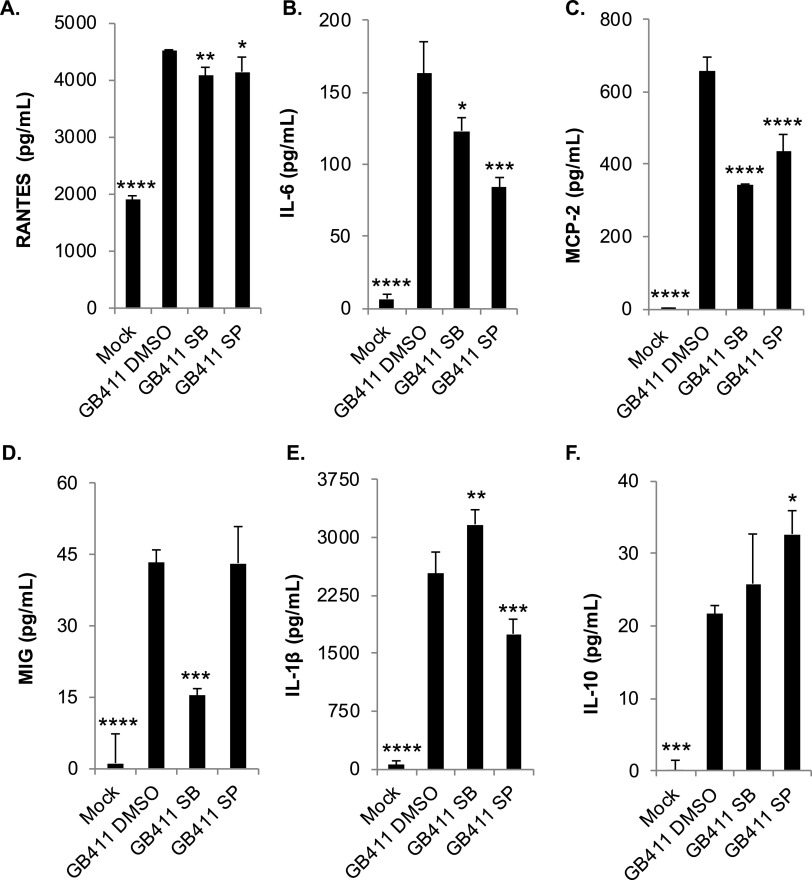
p38 and JNK MAPK influence GBS-mediated cytokine activation. THP-1 macrophages were treated with a p38 inhibitor (SB203580, 10 μM), a JNK inhibitor (SP600125, 25 μM), or a vehicle control (DMSO) 1.25 h prior to infecting them with GBS at a MOI of 10 for 1 h. They were washed and treated with antibiotics for an additional hour prior to collecting cell culture supernatants for cytokine analysis. Data from at least three independent biological replicates were pooled to determine the average cytokine concentrations (pg/ml) produced under each of the infection conditions shown. The average and standard deviation of each condition were plotted for comparison, and significance was determined by ANOVA, followed by post-ANOVA Dunnett’s tests (*, *P* = 0.01 to 0.05; **, *P* = 0.001 to 0.01; ***, *P* = 0.0001 to 0.001; ****, *P* < 0.0001) to compare the mean of each condition to the mean of the GB411 vehicle control infection condition (GB411 DMSO). All cytokines tested had ANOVA *P* values of <0.0001.

To elucidate whether the activation of p38, JNK, and their downstream mediators contributed to the GBS-induced cytokine responses observed in our earlier study (7), we treated THP-1 macrophages with p38 and JNK inhibitors prior to infection with GB411. Inhibition of both p38 and JNK significantly reduced the production of RANTES, interleukin-6 (IL-6), and MCP-2 in response to GBS (Fig. 5A to C). Inhibition of p38 also significantly reduced the production of MIG (monokine induced by gamma interferon or CXCL9) following GBS infection, while JNK inhibition did not significantly alter the production of this cytokine (Fig. 5D). Interestingly, p38 inhibition significantly increased IL-1β in response to GBS, whereas JNK inhibition significantly reduced IL-1β (Fig. 5E). JNK inhibition also resulted in a significant increase in the production of the anti-inflammatory cytokine IL-10 following GBS infection but p38 inhibition had no effect on IL-10 production (Fig. 5F).

### p38 and JNK MAPK play contrasting roles in the regulation of GBS-mediated macrophage cell death.

**FIG 6:**
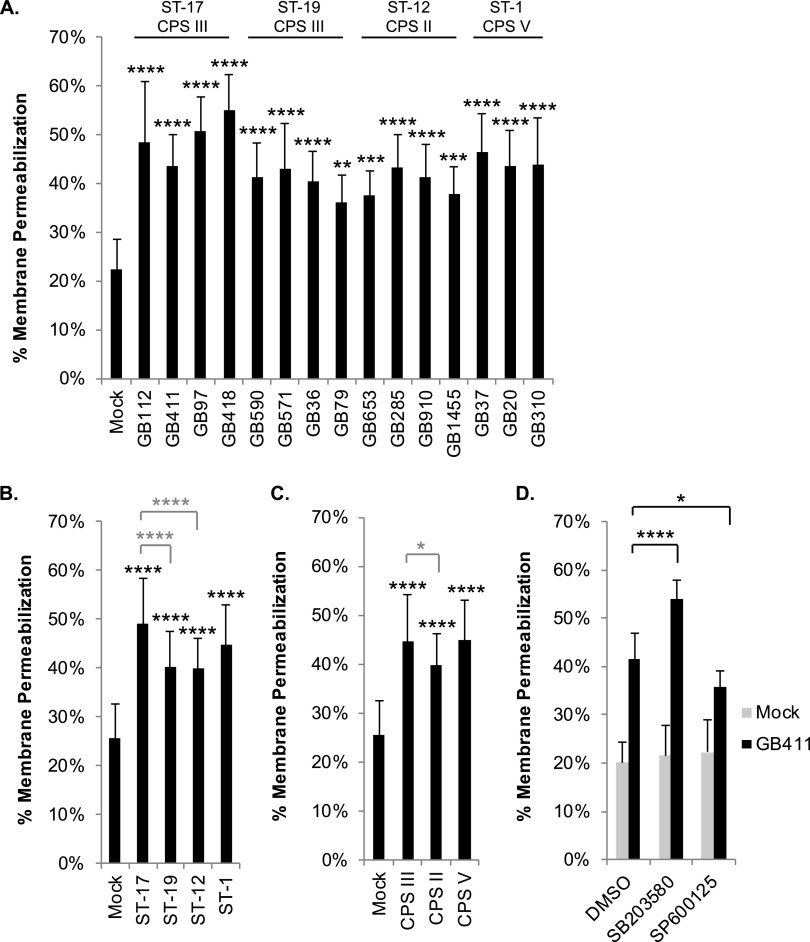
GBS-mediated cytotoxicity in macrophages is influenced by ST and CPS type and regulated by p38 and JNK MAPK. THP-1 macrophages were infected with GBS at a MOI of 10 for 1 h, washed, and treated with antibiotics for 24 h. Cell death was assessed using an ethidium homodimer membrane permeabilization assay. Results from at least three independent biological replicates performed in technical triplicate were pooled for each of the conditions analyzed. (A) ANOVAs and *post hoc* Dunnett’s tests were used to compare each infection condition to the mock infection. (B and C) ANOVA and *post hoc* Tukey’s tests were used to compare the mean of each ST or CPS group to the mean of every other ST or CPS group. Statistical comparisons between mock and infection conditions are shown with black asterisks, while comparisons between STs and CPS groups are shown in gray. (D) THP-1 macrophages were treated with a p38 inhibitor (SB203580, 10 μM), a JNK inhibitor (SP600125, 25 μM), or a vehicle control (DMSO) 1.25 h prior to infection with GBS at a MOI of 10 for 1 h. ANOVA and *post hoc* Dunnett’s tests were used to compare the p38 and JNK inhibitor conditions (10 μM SB203580 and 25 μM SP600125, respectively) to the corresponding vehicle control (DMSO). ANOVA *P* values for all comparisons were <0.0001. Dunnett’s test significance values: *, *P* = 0.01 to s0.05; **, *P* = 0.001 to 0.01; ***, *P* = 0.0001 to 0.001; ****, *P* < 0.0001.

In addition to regulating inflammatory responses, the stress-activated MAPKs, p38 and JNK, are key mediators of cell death (16). Depending on the specific cellular and environmental conditions, these mediators have been shown to either promote or inhibit cell death. Using an ethidium homodimer membrane permeabilization assay, we compared changes in THP-1 cell viability 24 h postinfection. Significantly enhanced cell death was observed in response to all 15 GBS strains compared to mock infection (Fig. 6). When the strains were grouped by STs, significantly higher levels of cell death were observed in response to the ST-17 strains compared to the ST-19 and ST-12 strains (Fig. 6B). ST-1 strains induced macrophage death at levels similar to the those of ST-17 strains. When grouped by serotype, CPS III strains induced significantly higher levels of cell death than CPS II strains but were similar to the CPS V strains (Fig. 6C). Notably, treatment of the THP-1 macrophages with p38 and JNK inhibitors prior to infection with GB411 contributed to significant changes in cell death. Inhibition of p38 resulted in a significant increase in macrophage cell death in response to GBS infection, while inhibition of JNK caused a significant decrease (Fig. 6D).

### GBS strains exhibit variation in phagocytic uptake and in their ability to survive in macrophages.

**FIG 7:**
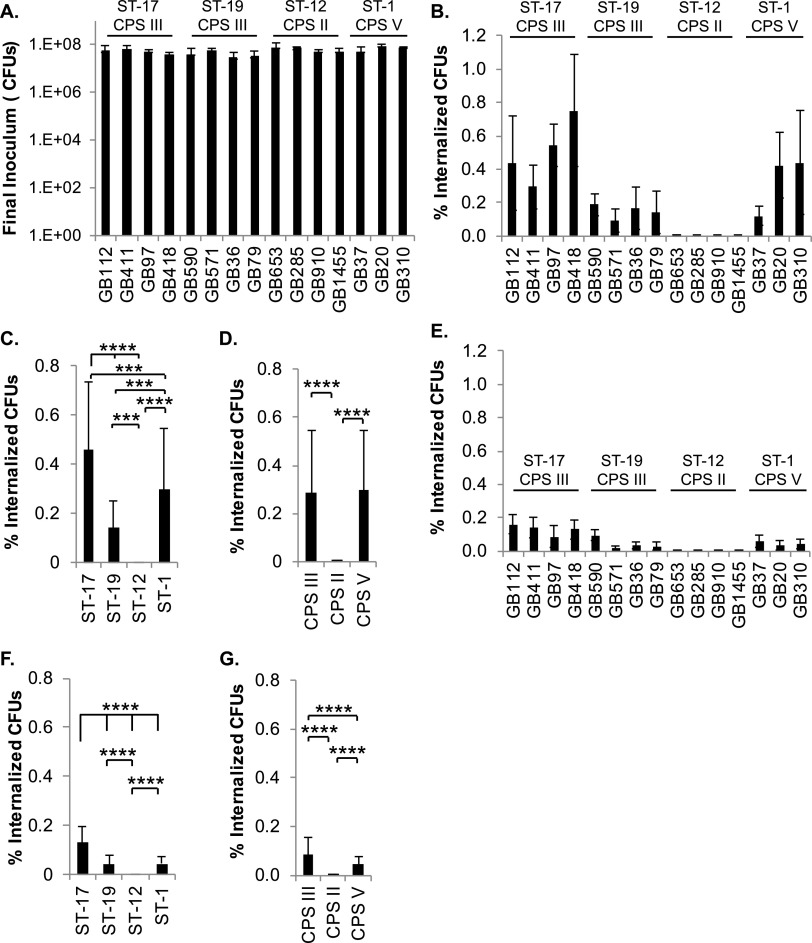
GBS strains exhibit variation in phagocytic uptake and survival in macrophages. (A) THP-1 macrophages were infected with GBS at a MOI of 10 for 1 h prior to the collection of a final inoculum sample. (B to G) Cells were washed and treated with antibiotics for 1 h (B to D) or 24 h (E to G). The percentage of internalized, viable GBS relative to the final inoculum was determined by CFU counting at both time points. Results from at least three independent biological replicates performed in technical triplicate were pooled for each of the conditions analyzed. In panels A, B, and E, ANOVAs and *post hoc* Tukey’s tests were used to compare the mean of each infection condition to the mean of every other condition. Statistical results for these comparisons are summarized in Table S3 due to the large number of significant results. In panels C, D, F, and G, ANOVA and *post hoc* Tukey’s tests were used to compare the mean of each ST or CPS group to the mean of every other ST or CPS group. ANOVA *P* values for all comparisons were <0.0001. Tukey’s test significance values: *, *P* = 0.01 to 0.05; **, *P* = 0.001 to 0.01; ***, *P* = 0.0001 to 0.001; ****, *P* < 0.0001.

The antibody array identified many proteins involved in pathways that regulate cytoskeletal rearrangements and downstream immunological responses, such as Fc gamma R-mediated phagocytosis and Toll-like receptor signaling. Based on these observations and data generated in our prior study (10), we investigated whether specific GBS traits impact the rate of phagocytic uptake by macrophages or the ability to survive within the macrophages after phagocytosis. Our results regarding ST or CPS-based differences in phagocytic uptake show that the ST-17 stains had significantly greater percentages of internalized bacteria relative to other STs after 1 h (Fig. 7A to C). Macrophage viability was similar among a subset of GBS strains examined at this 1-h time point in our initial analyses (data not shown). Together, these data suggest that ST-17 strains are phagocytosed by macrophages at significantly higher rates than other STs. When the strains were grouped by serotype, we observed equal phagocytic uptake of the CPS III and CPS V strains, with notably lower uptake of CPS II strains by comparison (Fig. 7B and D).

When the viability of the internalized GBS was assessed at 24 h, similar trends were observed. ST-17 strains had the most viable bacteria relative to the final inoculum, followed by ST-19 and ST-1 strains, with ST-12 strains having the lowest numbers of viable bacteria (Fig. 7E and F). When grouped by serotype, CPS III strains had the most viable bacteria, followed by CPS V strains, with CPS II strains having minimal viable bacteria remaining (Fig. 7E and G).

### MAPK signaling influences phagocytosis and survival of GBS in macrophages.

**FIG 8:**
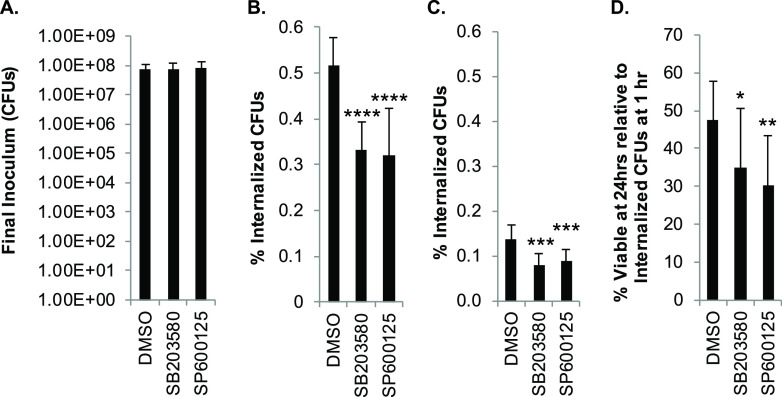
p38 and JNK signaling influence phagocytosis and survival of GBS in macrophages. (A) THP-1 macrophages were treated with a p38 inhibitor (SB203580, 10 μM), a JNK inhibitor (SP600125, 25 μM), or a vehicle control (DMSO) 1.25 h prior to GBS infection at a MOI of 10 for 1 h and collection of the final inoculum sample. (B and C) Cells were washed and treated with antibiotics for 1 h (B) or 24 h (C). The percentage of internalized, viable GBS relative to the final inoculum was determined via CFU counting at both time points. (D) The percentage of viable, internalized bacteria at 24 h was also normalized to the percentage of viable, internalized bacteria at 1 h for the corresponding treatment condition. Results from at least three independent biological replicates performed in technical triplicate were pooled for each of the conditions analyzed. ANOVAs (*P* values: A, *P* = 0.506; B and C, *P* < 0.0001; D, *P* = 0.0045) and *post hoc* Dunnett’s tests (*, *P* = 0.01 to 0.05; **, *P* = 0.001 to 0.01; ***, *P* = 0.0001 to 0.001; ****, *P* < 0.0001) were used to compare the mean of each inhibitor condition to the mean of the DMSO vehicle control condition.

To determine the role of p38 and JNK in phagocytic uptake of GBS, GB411 was used to infect THP-1 macrophages for 1 h following treatment with p38 or JNK inhibitors. Notably, inhibition by both p38 and JNK significantly reduced GBS uptake by macrophages at the 1-h time point (Fig. 8A and B). We also assessed the role of p38 and JNK in GBS survival in macrophages 24 h following antibiotic treatment. Inhibition of both p38 and JNK resulted in significantly lower numbers of viable GBS inside macrophages than in the vehicle controls (Fig. 8C). This trend was maintained when the number of viable bacteria at 24 h was normalized to the number of internalized bacteria at the 1-h time point for the corresponding infection conditions (Fig. 8D).

## DISCUSSION

Through our array analysis of the macrophage response to GBS infection, we identified numerous general and strain-specific changes in cellular signaling cascades. Some of the key changes in macrophage signaling involved activation of the p38 and JNK MAPK pathways following GBS infection. Our subsequent studies revealed that changes in the activation of these pathways are important for the regulation of many downstream responses to GBS, including NF-κB activation, cytokine production, macrophage cell death, GBS phagocytic uptake, and GBS survival in macrophages following phagocytosis. Of note, many of the specific proteins and signaling pathways affected in THP-1 cells were similar to those identified in our prior study examining GBS infection in decidual human endometrial stromal cells (dT-HESCs), while others were unique to macrophages (18).

Some of the observed responses were found to be regulated by both p38 and JNK, while others were regulated primarily by one or the other. For example, inhibition of JNK reduced NF-κB activation more dramatically than inhibition of p38. These results suggest that both p38 and JNK contribute to NF-κB activation following GBS infection of macrophages but that JNK may be the more significant upstream mediator of this response. Similarly, though both p38 and JNK aid in the modulation of the macrophage cytokine response to GBS infection, they play redundant roles with respect to some cytokines (i.e., RANTEs, IL-6, and MCP-2) and distinct (i.e., MIG, IL-10) or contrasting (i.e., IL-1β) roles with respect to others. Additional differences were observed for GBS-induced macrophage cell death. Inhibition of p38 significantly increased cell death, indicating that the observed activation of p38 by GBS has a prosurvival effect in the infected macrophages. In contrast, inhibition of JNK caused a significant decrease in macrophage cell death, suggesting that signaling changes in JNK may contribute to the induction of cell death in macrophages in response to GBS infection. When we assessed the roles of p38 and JNK with respect to GBS uptake by macrophages and GBS survival within macrophages, however, their roles were similar. Both p38 and JNK promoted GBS phagocytosis by macrophages as well as GBS survival within macrophages since greater bacterial numbers were observed in the absence of the inhibitors for these proteins.

Our results are largely supported by prior studies that have demonstrated GBS-mediated activation of p38, JNK, and NF-κB pathways in macrophages and other phagocytes (19–33). However, our work expands upon these prior studies by analyzing a more comprehensive assortment of host signaling proteins as well as 15 unique clinical isolates, including four GBS STs and three serotypes. To our knowledge, most analyses focusing on the role of these signaling responses during GBS infection have been limited to a single GBS strain, have utilized heat-killed or otherwise inactivated GBS, or have assessed the host response to purified GBS components rather than live bacteria (19–33).

Through our comparisons of key macrophage responses to these distinct ST and CPS groups, we observed that ST-17 strains, which have been linked to more severe disease outcomes than other STs (15, 34), induced the most robust stress and inflammatory signaling responses. Of the three serotypes, CPS III induced the most dramatic increase in stress and inflammatory pathway signaling followed by CPS V. These CPS types have previously been associated with severe disease (8, 10, 15, 34, 35). Here, we provided a link between these epidemiological and bacterial virulence factor-based studies and the macrophage inflammatory response at the cellular level. Our findings indicate that some of the reported differences in the virulence of these strains may be explained by their differential ability to activate key stress and inflammatory pathways in infected macrophages.

Differences in stress and inflammatory signaling may, in turn, be tied to differential uptake and survival in macrophages. Our data show that ST-17 strains are phagocytosed by macrophages at significantly higher rates than other STs, which may partially explain the greater impact these strains have on MAPK signaling in macrophages. This work expands upon our prior studies demonstrating strain-specific differences in GBS phagocytic uptake and survival within macrophages (10). By assessing uptake and long-term survival within macrophages using a larger assortment of clinical isolates, we have further supported our conclusion that ST-17 strains are phagocytosed either more rapidly or to a greater extent than other STs and that they are more resistant to phagocytic killing. Interestingly, these findings conflict with our prior study using heat-inactivated versions of many of the same strains, in which no link between phagocytic uptake and ST or CPS type was observed (19). This discrepancy indicates that the virulence factors driving differential macrophage uptake and survival require active changes in protein expression, which can only be achieved by live bacteria. Furthermore, we observed that macrophages require p38 and JNK to efficiently phagocytose GBS. Generally, this would be considered a beneficial host response to assist with clearing the bacterial infection. The activation of these pathways, however, promotes the survival of GBS inside macrophages once it is internalized, which could enhance pathogenesis. Indeed, this may be an important mechanism for GBS movement across protective tissue barriers to promote dissemination in cases of severe disease.

**FIG 9:**
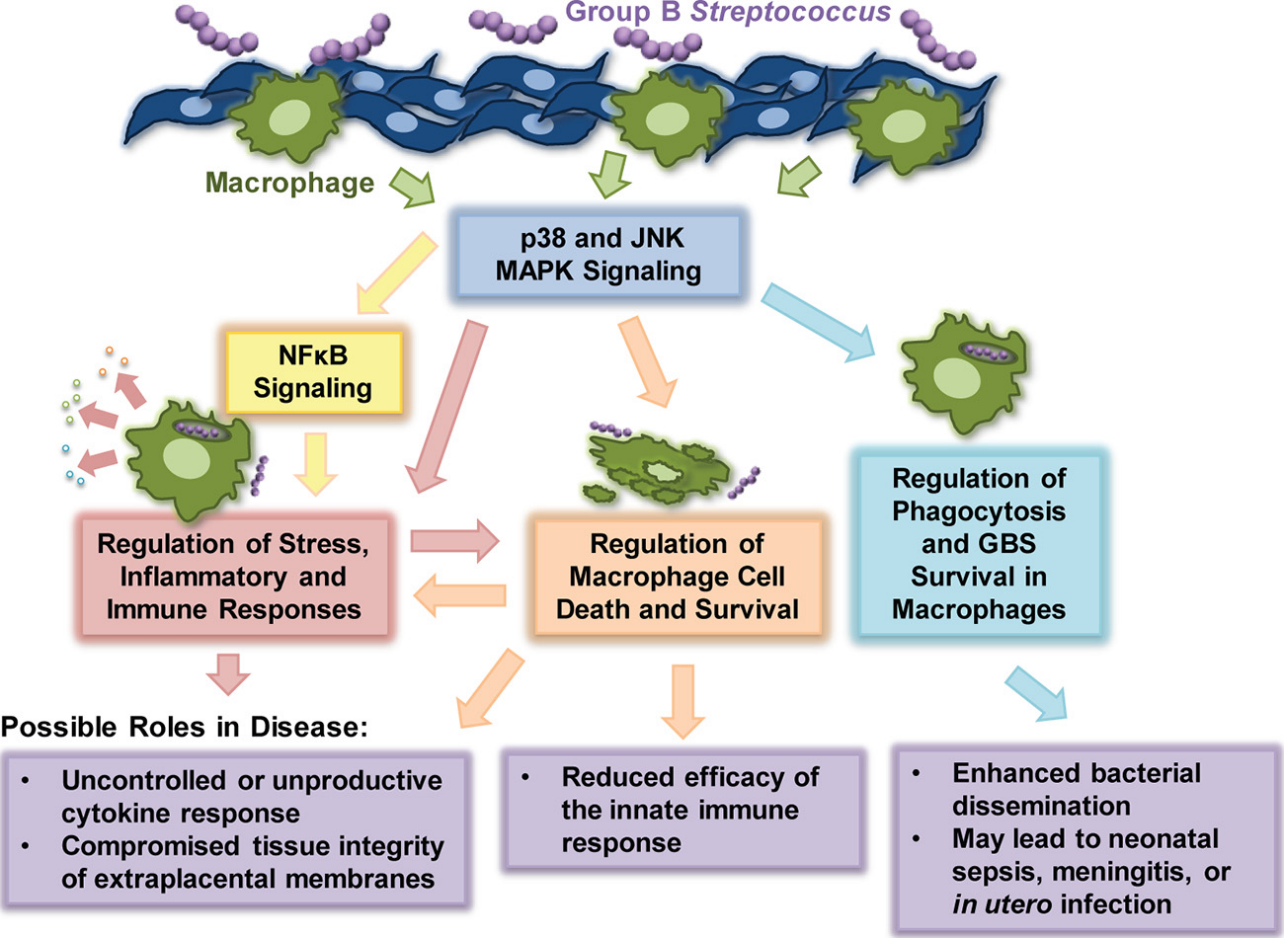
Model of responses and disease outcomes downstream of GBS-induced p38 and JNK signaling in macrophages.

Overall, we demonstrated enhanced activation of the MAPK and NF-κB pathways in response to certain GBS STs and CPS types and confirmed the role of these effectors in the induction of several important macrophage responses to GBS infection (Fig. 9). Such findings are critical to guide the development of diagnostic strategies to identify higher-risk GBS infections as well as p38 and JNK MAPK-based therapeutics to reduce the severity of GBS infection. Nonetheless, future work is needed to explore the physiological implications of these findings *in vivo* to confirm that the observed host responses are consistent with those occurring in human extraplacental membranes (EPMs). Comparing the activation of these inflammatory mediators in EPMs from mothers with pre- and full-term deliveries will indicate how well our model mimics the GBS-macrophage interaction in this particular context. Additional *in vivo* studies are also needed to determine whether the observed changes in host cell signaling primarily benefit the host or the pathogen. Use of a pregnant murine model, for example, could confirm if inhibiting these inflammatory mediators reduces preterm birth or fetal demise rates. These approaches are important next steps for the development of therapeutic interventions capable of alleviating harmful GBS-induced inflammation during pregnancy or in neonates suffering from GBS-induced complications.

## MATERIALS AND METHODS

### Bacterial strains.

This study utilized 15 previously characterized GBS strains, which were isolated from neonates with invasive disease (15) or colonized mothers before or after childbirth (34). Strains were selected based on ST, CPS, and source (Table 2). Differential cytokine production induced in THP-1 macrophages was observed with these strains previously (7), as was variation in phagocytic uptake using heat-inactivated versions of the strains (19). Five strains (GB00112 and GB00411 [ST-17], GB00590 [ST-19], GB00653 [ST-12, and GB00037 [ST-1]) were used for the antibody array analyses assessing changes in cell signaling proteins, while the remaining 10 strains were evaluated using additional biochemical analyses to further assess hits and signaling pathways identified in the arrays. Strains were grown at 37°C for 16 to 20 h in Todd-Hewitt broth (THB), subcultured to THB and grown to log phase (optical density at 600 nm [OD_600_] of 0.4), washed in sterile phosphate-buffered saline (PBS), and resuspended in RPMI 1640 (Gibco) prior to infection.

**TABLE 2:** Characteristics of the group B streptococcal strains used in the study^*a*^

Strain ID	ST	CPS	Clinical type
GB00112	17	III	Colonizing
GB00411	17	III	Invasive
GB00097	17	III	Colonizing
GB00418	17	III	Invasive
GB00590	19	III	Colonizing
GB00571	19	III	Colonizing
GB00036	19	III	Invasive
GB00079	19	III	Invasive
GB00653	12	II	Colonizing
GB00285	12	II	Colonizing
GB00910	12	II	Invasive
GB01455	12	II	Invasive
GB00037	1	V	Invasive
GB00020	1	V	Colonizing
GB00310	1	V	Invasive

a The sequence type (ST) and capsular (CPS) type as determined by multilocus sequence typing (MLST) and molecular serotyping, respectively, are shown for each strain along with the clinical type. Colonizing strains were isolated from mothers during pre- or postnatal screening visits (34), while invasive strains were recovered from symptomatic neonates with early- or late-onset GBS disease (15).

### THP-1 cell culture and infection.

THP-1 cells (ATCC TIB-202) were cultured at 37°C with 5% CO_2_ in RPMI 1640 medium supplemented with 2 mM l-glutamine (Gibco), 10% fetal bovine serum (FBS; Atlanta Biologicals), and 1% penicillin/streptomycin (Gibco); phorbol 12-myristate 13-acetate (PMA; Sigma) was used to differentiate cells into macrophage-like cells as described (7, 10). The cells were seeded at a density of 1 × 10^6^ cells per well in 24-well plates or at 4 × 10^6^ cells per well in 6-well plates.

PMA-treated THP-1 cells were washed with PBS, and fresh RPMI 1640 medium was added prior to infection with GBS at a multiplicity of infection (MOI) of 10 bacteria per host cell as described previously (7). Briefly, the infected cells were incubated for 1 h at 37°C with 5% CO_2_. Following aspiration of extracellular bacteria and medium, the cells were washed, and fresh RPMI 1640 with 2% FBS, 100 μg/ml gentamicin (Gibco), and 5 μg/ml penicillin G (Sigma) were added. Cells were incubated for up to 24 h at 37°C with 5% CO_2_. Antibiotics were maintained in the cell culture medium until sample collection to inhibit the growth and survival of extracellular GBS.

### Antibody array.

The KAM-900P antibody microarray kit (Kinexus; Vancouver, Canada) was used to analyze changes in the activity, regulatory state, or total protein level of 386 different human proteins in response to GBS infection as described in our prior study (18). After infection of THP-1 cells with GBS (MOI, 10) for 1 h, cells were washed with PBS, treated with antibiotics, and incubated for 1 h. Cells were then washed twice with PBS, lysed, and disrupted using microprobe sonication (Branson Sonifier 250). Protein collection, labeling, and array incubation conditions were performed according to the manufacturer’s instructions. Statistics to identify significant changes between each infection condition and the mock infection control were performed as described previously (18). Proteins with significantly different total or phosphorylated levels were annotated based on function and grouped into major signaling pathways using the KEGG and STRING software programs as well as the UniProt protein database (36).

### SDS-PAGE and Western blotting.

Bicinchoninic acid assays (BCA) (Pierce) with bovine serum albumin (BSA) protein standards were utilized to determine the protein concentration per lysate. Normalized protein lysates were loaded onto a 4 to 15% polyacrylamide gel (Bio-Rad). Samples were transferred to a polyvinylidene difluoride (PVDF) membrane, blocked, washed, incubated with primary and secondary antibodies, and developed as was done previously (18). Densitometry was performed using ImageJ to determine relative protein levels. GAPDH (glyceraldehyde-3-phosphate dehydrogenase) and beta-tubulin were used as loading controls. Graphed densitometry data were pooled from at least 3 independent biological replicates.

### Immunofluorescence staining and imaging.

Cells were plated in 24-well cell culture-treated plates, and GBS was added as described (18). Following infection, cells were washed, fixed, blocked, and incubated with primary and secondary antibodies and DAPI nuclear stain (18); imaging was performed using a BioTek Cytation 3 imager (×20 objective), and captured images were processed using ImageJ and Adobe Photoshop. A minimum of three biological replicates were performed per condition, and at least three fields were captured and counted. Statistics were calculated based on at least 2.2 × 10^4^ cells per condition, which were pooled from the three biological replicates. Graphs represent averaged values from each replicate, and error bars indicate the standard deviation from the mean.

### Antibodies and stains.

Antibodies to NF-κB p65 (no. 8242S), beta-tubulin (no. 2128S), GAPDH (no. 5174S), phospho-MAPK p38 (T180+Y182; no. 4511S), total MAPK p38 (no. 8690S), and total SAPK/JNK (no. 9252T) were obtained from Cell Signaling Technology. Antibodies to phospho-JNK (T183+Y185; no. 6254), phospho-FOS (S374; no. 81485), total c-FOS (no. 166940), phospho-JUN (S63; no. 822), and total c-JUN (no. 74543) were obtained from Santa Cruz Biotechnology. Goat anti-rabbit and anti-mouse IgG-horseradish peroxidase (HRP) secondary antibodies (no. 31460 and no. 31430) were obtained from Thermo Fisher Scientific. DAPI nuclear stain was obtained from Cell Signaling, and goat anti-rabbit IgG AlexaFluor488 was obtained from Molecular Probes (Life Technologies).

### Vehicle controls and chemical inhibitors.

Dimethyl sulfoxide (DMSO; Sigma) was used as the chemical solvent and vehicle control for all experiments utilizing chemical inhibitors. SB203580 (Cell Signaling) was used to inhibit p38 MAPK activity at a final concentration of 10 μM, and SP600125 (Cell Signaling) was used at a concentration of 25 μM to inhibit JNK. Each inhibitor was added to cell culture medium and applied to THP-1 cells for 1.25 h prior to GBS infection. These inhibitors were previously tested on GBS at time points and drug concentrations matching or exceeding experimental conditions to ensure that there were no off-target effects on GBS growth and viability (18).

### Ethidium homodimer cell death assays.

THP-1 cells were plated in 24-well tissue culture plates and infected with GBS (MOI, 10) under the conditions described above. Cell death was determined using the membrane permeabilization method described by Flaherty et al. (36). After infection, the cells were washed and incubated with 4 μM ethidium homodimer 1 (Fisher Scientific) in PBS. Fluorescence (528 nm excitation and 617 nm emission) was determined using a BioTek Cytation 3 plate reader. Saponin (0.1%; Sigma) was applied to each well to lyse the entire cell population, and fluorescence was remeasured. Percentage membrane permeabilization values were obtained by dividing the post-treatment fluorescence reading by the post-saponin fluorescence reading. At least 3 independent biological replicates were performed and pooled per condition, each of which was performed in technical triplicate (at minimum). The pooled averages of each condition were plotted, and error bars represent standard deviation of the mean.

### Macrophage survival assays.

As described previously (10), THP-1 cells were infected with GBS (MOI, 10) for 1 h, washed with PBS, treated with 100 μg/ml of gentamicin and 5 μg/ml of penicillin G, and incubated for either 1 h or 24 h. Prior to aspirating and washing the cells, a sample of medium was collected, plated on Todd-Hewitt agar (THA), and incubated overnight to calculate the final GBS concentration. Medium and nonadherent bacteria were aspirated, and the THP-1 cells were washed twice with PBS and lysed with 0.1% Triton X-100 (Sigma). The number of viable internalized bacterial cells was determined at 1 h and 24 h by plating the lysates on THA and quantifying the CFU after overnight incubation. The percentage of internalized bacteria was calculated by dividing the number of CFU from these lysates by the final concentration (CFU). At least three biological replicates were performed per time point with technical triplicates. It is important to note that the antibiotics (100 μg/ml of gentamicin and 5 μg/ml of penicillin G) were maintained in the cell culture medium until sample collection at both the 1-h and 24-h time points to inhibit the growth and survival of extracellular GBS. These antibiotics were maintained until the ethidium homodimer 1 was added to evaluate membrane permeability (viability) of the THP-1 cells and prevent GBS escape from the macrophages and subsequent growth in the medium.

### Cytokine analysis using ELISA.

Cell culture medium was collected 24 h post-antibiotics and centrifuged (2,400 relative centrifugal force [rcf] for 10 min) to remove bacterial and cellular debris; supernatants were stored at −20°C. Samples were thawed on ice and centrifuged at 16,000 rcf for 5 min prior to cytokine analysis. The following kits were used: IL-1β human ELISA kit, EH2IL1B2 (Thermo Fisher Scientific); MIG human ELISA kit, EHCXCL9 (Thermo Fisher Scientific); IL-6 human ELISA kit, KHC0061 (Thermo Fisher Scientific); MCP-2 human ELISA kit, EHCCL8 (Thermo Fisher Scientific); RANTES human ELISA kit, EHRNTS (Thermo Fisher Scientific); and IL-10 human ELISA kit, ab100549 (Abcam). MIG is often referred to as CXCL9 (C-X-C motif chemokine ligand or monokine induced by gamma interferon), and MCP-2 and RANTES are referred to as CCL8 (C-C motif chemokine 8) and CCL5 (C-C motif chemokine ligand 5). The average cytokine concentrations (pg/ml) were quantified per condition, and the standard deviation of the mean was calculated using pooled data from at least 3 independent biological replicates.

### Data analysis.

Graph Pad Prism 8.0 or Microsoft Excel was used for statistical analyses; *P* < 0.05 was considered significant. ANOVA was used to test for differences followed by *post hoc* Dunnett’s or Tukey’s tests. Dunnett’s tests were used to compare the mean for each treatment condition to the control mean per experiment. Tukey’s tests were used to compare the mean of each condition to the mean of all others. Individual *P* values from *post hoc* Dunnett’s tests and *post hoc* Tukey’s tests were reported as follows for all comparisons: *, *P* = 0.01 to 0.05; **, *P* = 0.001 to 0.01; ***, *P* = 0.0001 to 0.001; ****, *P* < 0.0001).

## Supplementary Material

Supplemental file 2

Supplemental file 4

Supplemental file 3

Supplemental file 1
