# Supplementary material for: Distinct Group B Streptococcus Sequence and Capsule Types Differentially Impact Macrophage Stress and Inflammatory Signaling Responses
Source: Infect Immun. 2021 Apr 16;89(5):e00647-20. doi: 10.1128/IAI.00647-20 (PMC8091095; doi:10.1128/IAI.00647-20)
Supplement: Supplemental file 2 [file IAI.00647-20-s0002.pdf]

Table S2: Array Analysis with Averaged Fold Changes for All Strains Compared to Mock Infection

Target Protein Name	Phospho Site (Human)	Full Target Protein Name	Average Fold Change
Cofilin 1	S3	Cofilin 1 (CFL1)	21.30
STAT1	Y701	Signal transducer and activator of transcription 1 beta	4.77
JNK2	Pan-specific	Jun N-terminus protein-serine kinase (stress-activated protein kinase (SAPK)) 2 (SAPKa, MAPK9)	3.67
p38a MAPK	Pan-specific	Mitogen-activated protein-serine kinase p38 alpha (MAPK14)	3.65
KIT	Y936	'Mast/stem cell growth factor receptor Kit	3.32
p38a MAPK	Pan-specific	Mitogen-activated protein-serine kinase p38 alpha (MAPK14)	3.16
InsR (IR)	Pan-specific	Insulin receptor beta chain	3.04
ACTA1	Pan-specific	ACTA1 (Alpha-actin)	3.03
STAT3	Pan-specific	Signal transducer and activator of transcription 3 (acute phase response factor)	2.93
PKCl/I	T564	Protein-serine kinase C lambda/iota (PRKCI)	2.91
Connexin 43	S368	Gap junction alpha-1 protein (Cx43, GJA1)	2.83
KSR	Pan-specific	Protein-serine kinase suppressor of Ras 1	2.63
p70 S6K	T252	Ribosomal protein S6 kinase beta-1 (RPS6KB1, p70S6Ka)	2.60
JAK1	Y1034	Janus protein-tyrosine kinase 1	2.55
STAT5A	Pan-specific	Signal transducer and activator of transcription 5A	2.53
STAT5B	Pan-specific	Signal transducer and activator of transcription 5B	2.39
RelB	Pan-specific	Transcription factor RelB	2.15
p70 S6K	S447	Ribosomal protein S6 kinase beta-1 (RPS6KB1, p70S6Ka)	1.97
TEC	Y519	Tyrosine-protein kinase Tec	1.95
NEK2	T170+S171	NIMA (never-in-mitosis)-related protein-serine kinase 2	1.94
eIF4G	S1106	Eukaryotic translation initiation factor 4 gamma 1 (eIF4G1)	1.93
PKCq	Pan-specific	Protein-serine kinase C theta (PRKCQ)	1.93
IGF1R	Y1280	Insulin-like growth factor 1 receptor protein-tyrosine kinase	1.91
IKKa	Pan-specific	Inhibitor of NF-kappa-B protein-serine kinase alpha (CHUK, IkBKA)	1.82
COT	Pan-specific	Osaka thyroid oncogene protein-serine kinase (TPL2) (MAP3K8)	1.77
MEK3/6	S218/S207	MAPK/ERK protein-serine kinase 3 beta isoform (MKK3 beta, MAP2K3)	1.63
Tau	T522	Microtubule-associated protein tau	1.60
KIT	Y721	'Mast/stem cell growth factor receptor Kit	1.55
SRC	Pan-specific	Src proto-oncogene-encoded protein-tyrosine kinase	1.51
FOS	Pan-specific	Fos-c FBJ murine osteosarcoma oncoprotein-related transcription factor	1.50
p53	S37	Tumor suppressor protein p53 (antigenNY-CO-13) (TP53)	1.49
STAT2	Pan-specific	Signal transducer and activator of transcription 2	1.49
MSK1	S376	Mitogen & stress-activated protein-serine kinase 1 (RPS6KA5)	1.49
RPS6	S235	40S ribosomal protein S6	1.47
InsR (IR)	Y999	Insulin receptor beta chain	1.47
SMC1	S957	Structural maintenance of chromosomes protein 1A	1.47
ITSN2	Y968	Intersectin-2	1.43
p53	S6	Tumor suppressor protein p53 (antigenNY-CO-13) (TP53)	1.43
RelB	S573	Transcription factor RelB	1.43
ROCK2	Pan-specific	Rho-associated protein kinase 2 (ROKa)	1.40
STAT5A	S780	Signal transducer and activator of transcription 5A	1.39
p38b MAPK	Pan-specific	Mitogen-activated protein-serine kinase p38 beta (MAPK11)	1.37
JUN	Pan-specific	Jun proto-oncogene-encoded AP1 transcription factor (c-Jun)	1.36
Vimentin	S34	VIM (Vimentin)	1.31

PAK2	S141	p21-activated kinase 2 (gamma) (serine/threonine-protein kinase PAK 2) (PAKg)	1.28
PRKACA	Pan-specific	cAMP-dependent protein kinase catalytic subunit alpha	1.27
Arrestin b	Pan-specific	Arrestin beta 1 (ARRB1)	1.24
Rb	S807	Retinoblastoma-associated protein 1	1.24
PAK2	Pan-specific	p21-activated kinase 2 (gamma) (serine/threonine-protein kinase PAK 2) (PAKg)	1.22
IkBe	S161	NF-kappa-B inhibitor epsilon	1.19
PKCd	S645	Protein-serine kinase C delta (PRKCD)	1.17
CDK1/2	Pan-specific	Cyclin-dependent protein-serine kinase 1/2 (CDC2)	1.17
p38d MAPK	Pan-specific	Mitogen-activated protein-serine kinase p38 delta (MAPK13)	1.17
mTOR	Pan-specific	Mammalian target of rapamycin (FRAP)	1.16
EphA3	Y779	Ephrin type-A receptor 3 protein-tyrosine kinase	1.15
Histone H2A.X	S139	Histone H2A variant X	1.15
PKCb	Pan-specific	Protein-serine kinase C beta 1 (PRKCB1)	1.13
p38b MAPK	Pan-specific	Mitogen-activated protein-serine kinase p38 beta (MAPK11)	1.12
Integrin a4	S1021	Integrin alpha 4 (VLA4, ITGA4)	1.10
p70 S6K	S434	Ribosomal protein S6 kinase beta-1 (RPS6KB1, p70S6Ka)	1.09
Catenin b	Pan-specific	Catenin (cadherin-associated protein) beta 1 (CTNNB1)	1.08
PKCl/I	Pan-specific	Protein-serine kinase C lambda/iota (PRKCI)	1.07
SYK	Pan-specific	Spleen protein-tyrosine kinase	1.06
CLK1	S337	Dual specificity protein kinase CLK1	1.05
Paxillin 1	Y118	Paxillin 1 (PXN)	1.03
GFAP	S8	Glial fibrillary acidic protein	1.00
mTOR	S2478+S2481	Mammalian target of rapamycin (FRAP)	0.99
p38g MAPK	Pan-specific	Mitogen-activated protein-serine kinase p38 gamma, ((MAPK12, ERK6)	0.99
MKK7	T275	MAPK/ERK protein-serine kinase 7 (MEK7, MAP2K7)	0.97
PAK2	Y130	p21-activated kinase 2 (gamma) (serine/threonine-protein kinase PAK 2) (PAKg)	0.97
ErbB3 (HER3)	Y1328	Tyrosine kinase-type cell surface receptor HER3	0.96
p38g MAPK	Pan-specific	Mitogen-activated protein-serine kinase p38 gamma, ((MAPK12, ERK6)	0.96
PKCb2	T642	Protein-serine kinase C beta 2 (PRKCB2)	0.94
PKCe	S729	Protein-serine kinase C epsilon (PRKCE)	0.93
Huntingtin	S421	Huntington's disease protein	0.92
PAK1	S144	p21-activated kinase 1 (alpha) (serine/threonine-protein kinase PAK 1) (PAKa)	0.92
PAK1	Pan-specific	p21-activated kinase 1 (alpha) (serine/threonine-protein kinase PAK 1) (PAKa)	0.92
PKR1	T446	Double stranded RNA dependent protein-serine kinase (PRKR; EIF2AK2)	0.91
CBL	Y700	Signal transduction protein CBL	0.90
WNK1	T60	Serine/threonine-protein kinase WNK1 (PRKWNK1)	0.90
MYC	S373	Myc proto-oncogene protein	0.89
PTEN	S380+T382+S385	Phosphatidylinositol-3,4,5-trisphosphate 3-phosphatase and protein phosphatase and tensin homolog deleted on chromosome 10	0.88
CDK1/2	Y15	Cyclin-dependent protein-serine kinase 1/2 (CDC2)	0.87
TYK2	Pan-specific	Protein-tyrosine kinase 2 (Jak-related)	0.86
HDAC5	S498	Histone deacetylase 5	0.86
MAPKAPK3	Y76	MAP kinase-activated protein kinase 3	0.86
p38a MAPK	T180+Y182	Mitogen-activated protein-serine kinase p38 alpha (MAPK14)	0.85
HSP90AB1	Pan-specific	Heat shock protein HSP 90-beta (HSP90B)	0.85
CDK1/2	Y15	Cyclin-dependent protein-serine kinase 1/2 (CDC2)	0.84

EGFR	Y1172	Epidermal growth factor receptor-tyrosine kinase (ErbB1)	0.84
PKCq	S695	Protein-serine kinase C theta (PRKCQ)	0.83
ErbB2 (HER2)	Y877	ErbB2 (Neu) receptor-tyrosine kinase	0.83
MEKK1	Pan-specific	MAPK/ERK kinase kinase 1 (MAP3K1)	0.82
IRS1	S312	Insulin receptor substrate 1	0.82
SYK	Y323	Spleen protein-tyrosine kinase	0.79
PDGFRa	Pan-specific	Platelet-derived growth factor receptor kinase alpha	0.78
PTEN	S380+T382+T383	Phosphatidylinositol-3,4,5-trisphosphate 3-phosphatase and protein phosphatase and tensin homolog deleted on chromosome 10	0.78
p53	S33	Tumor suppressor protein p53 (antigenNY-CO-13) (TP53)	0.77
PLCg1	Y771	1-phosphatidylinositol-4,5-bisphosphate phosphodiesterase gamma-1	0.77
eIF4B	S422	Eukaryotic translation initiation factor 4B	0.77
PKCg	T655	Protein-serine kinase C gamma (PRKCG)	0.77
PAK1	T212	p21-activated kinase 1 (alpha) (serine/threonine-protein kinase PAK 1) (PAKa)	0.76
LCK	Y394	Lymphocyte-specific protein-tyrosine kinase	0.76
p38b MAPK	T180+Y182	Mitogen-activated protein-serine kinase p38 beta (MAPK11)	0.75
Ron	Pan-specific	Macrophage-stimulating protein receptor alpha chain (RONa)	0.75
Raf1 (c-Raf)	Pan-specific	Raf1 proto-oncogene-encoded protein-serine kinase (RafC)	0.75
Histone H2B	S14	Histone H2B	0.74
SYK	Y352	Spleen protein-tyrosine kinase	0.74
STAT2	Y690	Signal transducer and activator of transcription 2	0.73
EGFR	Y1110	Epidermal growth factor receptor-tyrosine kinase (ErbB1)	0.72
NFkappaB p65	S536	NF-kappa-B p65 nuclear transcription factor (Rel A)	0.72
JUN	Y170	Jun proto-oncogene-encoded AP1 transcription factor (c-Jun)	0.72
CDK6	Y13	Cyclin-dependent protein-serine kinase 6	0.70
Rb	S780	Retinoblastoma-associated protein 1	0.69
EGFR	Y1197	Epidermal growth factor receptor-tyrosine kinase (ErbB1)	0.69
TRKB	Y706	BNDF/NT3/4/5 receptor- tyrosine kinase (NTRK2)	0.68
MET	Pan-specific	Hepatocyte growth factor (HGF) receptor-tyrosine kinase	0.66
Rb	Pan-specific	Retinoblastoma-associated protein 1	0.66
HSP90a/b	Pan-specific	Heat shock 90 kDa protein alpha/beta	0.66
NBS1	S343	Nijmegen breakage syndrome protein 1 (NBN, Nibrin)	0.66
JNK3	Pan-specific	Jun N-terminus protein-serine kinase (stress-activated protein kinase (SAPKb)) 3 (SAPKb, MAPK10)	0.66
TTK	Pan-specific	Dual specificity protein kinase	0.66
PAK2	Pan-specific	p21-activated kinase 2 (gamma) (serine/threonine-protein kinase PAK 2) (PAKg)	0.66
HDAC4	Pan-specific	Histone deacetylase 4	0.65
HDAC4/5/9	S246	Histone deacetylase 4/5/9	0.64
ELK1	Pan-specific	ETS domain-containing protein Elk-1	0.64
GSK3b	Pan-specific	Glycogen synthase-serine kinase 3 beta	0.64
Catenin a	S641	Catenin (cadherin-associated protein) alpha (CTNNA1)	0.64
FAK (PTK2)	Y576+Y577	Focal adhesion protein-tyrosine kinase	0.63
EphA2	Y772	Ephrin type-A receptor 2 protein-tyrosine kinase	0.63
SCYL1	S754	N-terminal kinase-like protein	0.63
MYC	T58	Myc proto-oncogene protein	0.61
CSK	Pan-specific	C-terminus of Src tyrosine kinase	0.61
DYRK2	Y382	Dual specificity tyrosine-phosphorylation-regulated kinase 2	0.61
Caveolin 2	Pan-specific	Caveolin 2 (CAV2)	0.61

GluR1	S849	Glutamate receptor 1	0.59
FKHR	S256	Forkhead box protein O1 (FOXO1A)	0.59
PKCm	Pan-specific	Protein-serine kinase C mu (Protein kinase D) (PRKD1, PKD1, PRKCM)	0.58
JUN	S243	Jun proto-oncogene-encoded AP1 transcription factor (c-Jun)	0.57
PKR1	Pan-specific	Double stranded RNA dependent protein-serine kinase (PRKR; EIF2AK2)	0.57
PLCg2	Y753	1-phosphatidylinositol-4,5-bisphosphate phosphodiesterase gamma-2 (PLC R)	0.56
IRS1	S639	Insulin receptor substrate 1	0.56
PAK1	Pan-specific	p21-activated kinase 1 (alpha) (serine/threonine-protein kinase PAK 1) (PAKa)	0.56
EFNB2	Y316	EPH-related receptor tyrosine kinase ligand 5	0.55
HCA59	Y147	Uncharacterized protein C9orf78 (HSPC220)	0.54
EGFR	Y1172	Epidermal growth factor receptor-tyrosine kinase (ErbB1)	0.54
AurKB (Aurora B)	S227	Aurora Kinase B (serine/threonine protein kinase 12), (AIM-1)	0.52
JAK1	Pan-specific	Janus protein-tyrosine kinase 1	0.51
LIMK1	Pan-specific	LIM domain kinase 1	0.50
KHS1	S174	Kinase homologous to SPS1/STE20 (MAP kinase kinase kinase protein-serine kinase 5 (MEKKK5)	0.49
Caveolin 1	Y14	Caveolin 1 (CAV1)	0.48
SYK	Y323	Spleen protein-tyrosine kinase	0.46
MST1	Pan-specific	Mammalian STE20-like protein-serine kinase 1 (KRS2, STK4)	0.46
Cofilin 1	Pan-specific	Cofilin 1 (CFL1)	0.46
RSK1	T359	Ribosomal S6 protein-serine kinase 1 (RPS6KA1, p90RSK)	0.46
AurKB (Aurora B)	Pan-specific	Aurora Kinase B (serine/threonine protein kinase 12), (AIM-1)	0.45
PKCe	Pan-specific	Protein-serine kinase C epsilon (PRKCE)	0.44
NR1 (NMDAR1)	S896	N-methyl-D-aspartate (NMDA) glutamate receptor 1 subunit zeta	0.43
HSP90AB1	Y484	Heat shock protein HSP 90-beta (HSP90B)	0.42
TBK1	Pan-specific	Serine/threonine-protein kinase TBK1	0.41
FOS	T232	Fos-c FBJ murine osteosarcoma oncoprotein-related transcription factor	0.40
Cyclin E1	T395	Cyclin E1 (CCNE1)	0.39
FGFR2	Y656+Y657	Fibroblast growth factor receptor-tyrosine kinase 2 (BEK)	0.38
PKCd	S664	Protein-serine kinase C delta (PRKCD)	0.37
MEK1/2	S218+S222	MAPK/ERK protein-serine kinase 1/2 (MKK1/2, MAP2K1/2)	0.32
NEK7	Pan-specific	NIMA (never-in-mitosis)-related protein-serine kinase 7	0.31
FGR	Y412	Gardner-Rasheed feline sarcoma viral (v-fgr) oncogene	0.29
IkBb	Pan-specific	Inhibitor of NF-kappa-B beta (thyroid receptor interacting protein 9)	0.28
TGM2	Y369	Protein-glutamine gamma-glutamyltransferase 2	0.27
BRCA1	S1497	Breast cancer type 1 susceptibility protein	0.25
ZAP70	Pan-specific	Zeta-chain (TCR) associated protein-tyrosine kinase, 70 kDa	0.24
IGF1R	Y1165/Y1166	Insulin-like growth factor 1 receptor protein-tyrosine kinase	0.23
ER-alpha	S104	Estrogen receptor alpha (ESR1)	0.15
PCTAIRE2	S180	Cell division protein kinase 17 (CDK17, PCTK2)	0.08
Bmx	Y40	Bone marrow X protein-tyrosine kinase (Etk)	0.08
p70 S6K	Pan-specific	Ribosomal protein S6 kinase beta-1 (RPS6KB1 p70S6Ka)	0.07
Crystallin aB	Pan-specific	Crystallin alpha B (heat-shock 20 kDa like-protein) (HspB5; CRYA2; CRYAB)	0.07
Cyclin B1	S147	Cyclin B1 (CCNB1)	0.06
GATA1	S142	Erythroid transcription factor	0.03
EGFR	Y1172	Epidermal growth factor receptor-tyrosine kinase (ErbB1)	0.01
BCR	Y177	Breakpoint cluster region protein	0.00

Histone H3	S10	Histone H3.3 (H3F3A)	-0.01
EGFR	T693	Epidermal growth factor receptor-tyrosine kinase (ErbB1)	-0.02
DNAPK	T2609	DNA-activated protein-serine kinase (PRKDC)	-0.04
CDK7	Pan-specific	Cyclin-dependent protein-serine kinase 7	-0.05
ERK5 (MAPK7)	Y221	Extracellular regulated protein-serine kinase 5 (Big MAP kinase 1 (BMK1))	-0.08
DAPK3	S269	Death-associated protein kinase 3 (DLK, MAP3K12)	-0.08
eEF1A1	Y141	Elongation factor 1-alpha 1	-0.12
InsR (IR)	Y1189	Insulin receptor beta chain	-0.39
APP	T743	Amyloid beta A4 protein	-0.40
PAK5	S602	p21-activated kinase 5 (serine/threonine-protein kinase PAK 7)	-0.40
RSK1	T573	Ribosomal S6 protein-serine kinase 1 (RPS6KA1, p90RSK)	-0.41
TYRO3	Y681	Tyrosine-protein kinase receptor TYRO3	-0.41
ATF2	S112	Activating transcription factor 2 (CRE-BP1)	-0.42
ASK1	S1046	Apoptosis signal regulating protein-serine kinase 1 (MAP3K5)	-0.43
CDK10	T196	Cyclin-dependent protein-serine kinase 10 (PISSLRE)	-0.45
SIK3 (QSK)	Pan-specific	Serine/threonine-protein kinase SIK3	-0.46
Catenin b	Pan-specific	Catenin (cadherin-associated protein) beta 1 (CTNNB1)	-0.46
CDKL5	Y171	Cyclin-dependent kinase-like 5 (STK9)	-0.55
CDK11A	T583	Cell division cycle 2-like 2 protein kinase (Cdc2L2)	-0.56
FRS2	Y348	Fibroblast growth factor receptor substrate 2	-0.56
CDK9	Pan-specific	Cyclin-dependent protein-serine kinase 9	-0.57
CHK1	S280	Checkpoint protein-serine kinase 1 (CHEK1)	-0.58
A6	Y309	Twinfilin-1	-0.58
DDR2	Y736	Discoidin domain-containing receptor 2 (Tyro10)	-0.58
BTK	Pan-specific	Bruton's agammaglobulinemia tyrosine kinase	-0.61
GUK1	Y53	Guanylate kinase	-0.62
snRNP 70	Y126	U1 small nuclear ribonucleoprotein 70 kDa	-0.63
AMPKa2	S377	5'-AMP-activated protein kinase catalytic subunit alpha-2 (PRKAA2)	-0.64
PKCh	Pan-specific	Protein-serine kinase C eta (PRKCH)	-0.64
eIF2a	Pan-specific	Eukaryotic translation initiation factor 2 alpha	-0.64
CDK1	T161	Cyclin-dependent protein-serine kinase 1 (CDC2)	-0.64
VEGFR3 (FLT4)	Pan-specific	Vascular endothelial growth factor receptor-protein-tyrosine kinase 3 (VEGFR3)	-0.65
EGFR	Y869	Epidermal growth factor receptor-tyrosine kinase (ErbB1)	-0.66
AKT2 (PKBb)	Pan-specific	RAC-beta serine/threonine-protein kinase	-0.66
p53	Pan-specific	Tumor suppressor protein p53 (antigenNY-CO-13) (TP53)	-0.67
Sgk223	Y413	Tyrosine-protein kinase SgK223	-0.67
ANXA2	Y238	Annexin A2	-0.69
CDK12	S383+S385	Cell division protein kinase 12 (Cdc2L7)	-0.71
RIPK2	Y381	Receptor-interacting serine/threonine-protein kinase 2 (RIPK2, RIP2, RICK)	-0.71
ANKRD3	S438	Ankyrin repeat domain protein-serine kinase 3 (RIPK4, DIK)	-0.75
ANXA1	Y207	Annexin A1	-0.75
PBK	Y74	Lymphokine-activated killer T-cell-originated protein kinase	-0.76
ACK1	Y518	Activated CDC42 kinase 1 (TNK2)	-0.78
FAK (PTK2)	Pan-specific	Focal adhesion protein-tyrosine kinase	-0.87
CDK2	Pan-specific	Cyclin-dependent protein-serine kinase 2	-0.90
ILK1	Pan-specific	Integrin-linked protein-serine kinase 1	-0.90
CSK	Pan-specific	C-terminus of Src tyrosine kinase	-1.03
