# Supplementary material for: Distinct Group B Streptococcus Sequence and Capsule Types Differentially Impact Macrophage Stress and Inflammatory Signaling Responses
Source: Infect Immun. 2021 Apr 16;89(5):e00647-20. doi: 10.1128/IAI.00647-20 (PMC8091095; doi:10.1128/IAI.00647-20)
Supplement: Supplemental file 4 [file IAI.00647-20-s0004.pdf]

Supplemental Material - Figures

Figure S1. GBS-mediated changes in MAPK proteins in THP-1 macrophages. THP-1 macrophages were infected with GBS at an MOI of 10 for 1 h, washed, and treated with antibiotics for an additional hour prior to lysate collection and subsequent antibody-based array analysis. Significantly altered MAPK pathway proteins in THP-1 macrophage lysates are shown. Total or phosphorylated protein values for each strain are expressed as fold change from mock infection, such that positive (red) values represent increases and negative (blue) values represent decreases. Phospho-sites marked with an asterisk were averaged from multiple antibodies targeting the same phospho-site.

MAPK Signaling Pathway Fold Changes Relative to Mock Infection						
Protein	Phospho-site	GBS Strains				
		GB112	GB411	GB590	GB653	GB37
ACK1	Y518	-0.66	-0.58	-1.01	-0.57	-1.07
AKT2 (PKBb)	Pan-specific	-0.45	-0.44	-0.97	-0.44	-1.01
ANKRD3	S438	-0.33	-0.59	-1.04	-0.69	-1.09
ANXA1	Y207	-0.57	-0.61	-0.92	-0.54	-1.13
ANXA2	Y238	-0.78	-0.30	-0.95	-0.42	-1.02
ASK1	S1046	-0.52	0.39	-0.29	-0.60	-1.10
ATF2	S112	-0.43	0.24	-0.67	-0.25	-0.99
COT	Pan-specific	0.94	3.04	1.07	1.80	1.99
EGFR	T693	-0.36	0.85	-1.04	1.05	-0.58
EGFR	Y1110	0.38	1.52	0.49	1.03	0.19
EGFR	Y1172*	1.25	0.40	0.34	0.29	0.02
EGFR	Y1197	0.08	1.78	0.11	1.39	0.11
EGFR	Y869	-0.04	-0.77	-1.08	-0.67	-0.72
ELK1	Pan-specific	0.11	1.60	0.85	0.56	0.10
FGFR2	Y656+Y657	2.21	0.05	0.05	-0.13	-0.27
FOS	Pan-specific	-1.07	6.95	0.70	0.74	0.17
FOS	T232	-0.30	0.60	0.19	1.50	0.04
IKKa	Pan-specific	2.10	1.27	1.81	1.61	2.29
JNK2	Pan-specific	2.36	4.51	2.96	4.15	4.39
JNK3	Pan-specific	1.24	0.12	0.78	0.33	0.83
JUN	Pan-specific	1.32	0.68	2.83	0.78	1.20
JUN	S243	-0.26	1.44	0.35	1.58	-0.25
JUN	Y170	-0.33	1.40	0.29	1.78	0.44
MEK1/2	S218+S222	0.24	0.71	1.34	-0.37	-0.33
MEK3/6	S218/S207	1.34	2.73	2.07	0.92	1.08
MKK7	T275	1.10	0.40	1.41	1.22	0.74
MEKK1	Pan-specific	0.42	0.73	1.04	0.85	1.06
MAPKAPK3	Y76	1.92	0.06	1.17	0.47	0.67
MKK7	T275	1.10	0.40	1.41	1.22	0.74
MSK1	S376	0.93	1.17	2.52	1.76	1.05
MYC	T58	0.46	0.93	1.07	0.04	0.56
NFkappaB p65	S536	0.01	1.27	0.96	0.94	0.42
PAK1	Pan-specific	1.44	0.57	0.01	2.46	0.09
PAK1	S144	2.26	0.17	0.26	1.40	0.50
PAK1	T212	0.78	1.82	0.46	0.60	0.15
PAK1	Pan-specific	1.76	-0.23	0.13	0.50	0.62
PAK2	Pan-specific*	2.24	1.02	0.10	1.14	0.21
PAK2	S141	1.53	0.77	0.57	1.89	1.63
PAK2	Y130	2.82	1.34	-0.23	0.43	0.50
PAK5	S602	0.93	-0.74	-0.86	-0.26	-1.07
PDGFRa	Pan-specific	0.95	0.21	0.66	0.60	1.49
PRKACA	Pan-specific	1.00	1.65	1.52	0.80	1.36
PKCb	Pan-specific	0.36	1.34	0.60	2.07	1.26
PKCb2	T642	-0.02	1.11	1.70	0.59	1.31
PKCg	T655	0.72	-0.12	1.00	0.31	1.94
p38a MAPK	T180+Y182	3.92	-0.36	0.45	0.02	0.24
p38a MAPK	Pan-specific*	3.85	2.06	3.84	3.50	3.77
p38b MAPK	T180+Y182	1.22	0.27	0.05	1.80	0.44
p38b MAPK	Pan-specific*	3.15	0.06	0.66	1.71	0.63
p38d MAPK	Pan-specific	1.58	1.05	1.51	0.62	1.10
p38g MAPK	Pan-specific*	0.93	0.32	1.61	1.20	0.80
p53	Pan-specific	-0.20	-0.42	-0.94	-0.65	-1.11
p53	S33	0.41	0.45	0.97	1.03	1.02
p53	S37	0.63	1.33	1.14	2.92	1.46
p53	S6	0.54	0.65	2.00	1.61	2.34
p70 S6K	Pan-specific	0.25	-0.60	-0.51	0.15	1.06
p70 S6K	S434	-0.02	1.29	1.57	0.56	2.08
p70 S6K	S447	0.40	2.12	1.75	2.87	2.68
p70 S6K	T252	1.56	3.26	3.73	3.70	0.77
Raf1 (c-Raf)	Pan-specific	0.13	0.65	0.76	0.76	1.43
RelB	Pan-specific	1.15	2.06	3.08	2.20	2.25
RelB	S573	0.49	1.35	2.58	1.26	1.45
RSK1	T359	-0.20	-0.12	0.31	1.67	0.62
RSK1	T573	0.70	-0.24	-0.92	-1.10	-0.52
Tau	T522	1.03	1.43	1.05	2.45	2.05
TRKB	Y706	0.07	0.53	0.05	1.62	1.15

Figure S2. GBS induces stress responsive MAPK activation in response to additional diverse clinical isolates. THP-1 macrophages were infected with additional GBS strains at an MOI of 10 for 1 h, washed, and treated with antibiotics for an additional hour prior to lysate collection. Lysates were assessed for phosphorylated (active) or total protein levels of p38 (**A, B, and E**), JNK (**C, D, and E**), and densitometry was used to compare differences between infection conditions. Densitometry values represent pooled results from at least three independent biological replicates, and error bars represent standard deviations of the mean. Significance was determined by ANOVA (p-values: phospho-p38, 0.04873; total p38, 0.9335; phospho-JNK, 0.0609, total JNK, 0.9985), with post-hoc Dunnett's testing to compare each infection condition to the mock infection (*, p=0.01-0.05; **, p=0.001-0.01; ***, p=0.0001-0.001; ****, p<0.0001). Representative Western blots from one biological replicate with its corresponding loading control (GAPDH) are shown (**E**). Equal amounts of the same protein lysate preparations were loaded onto the gels for each protein.

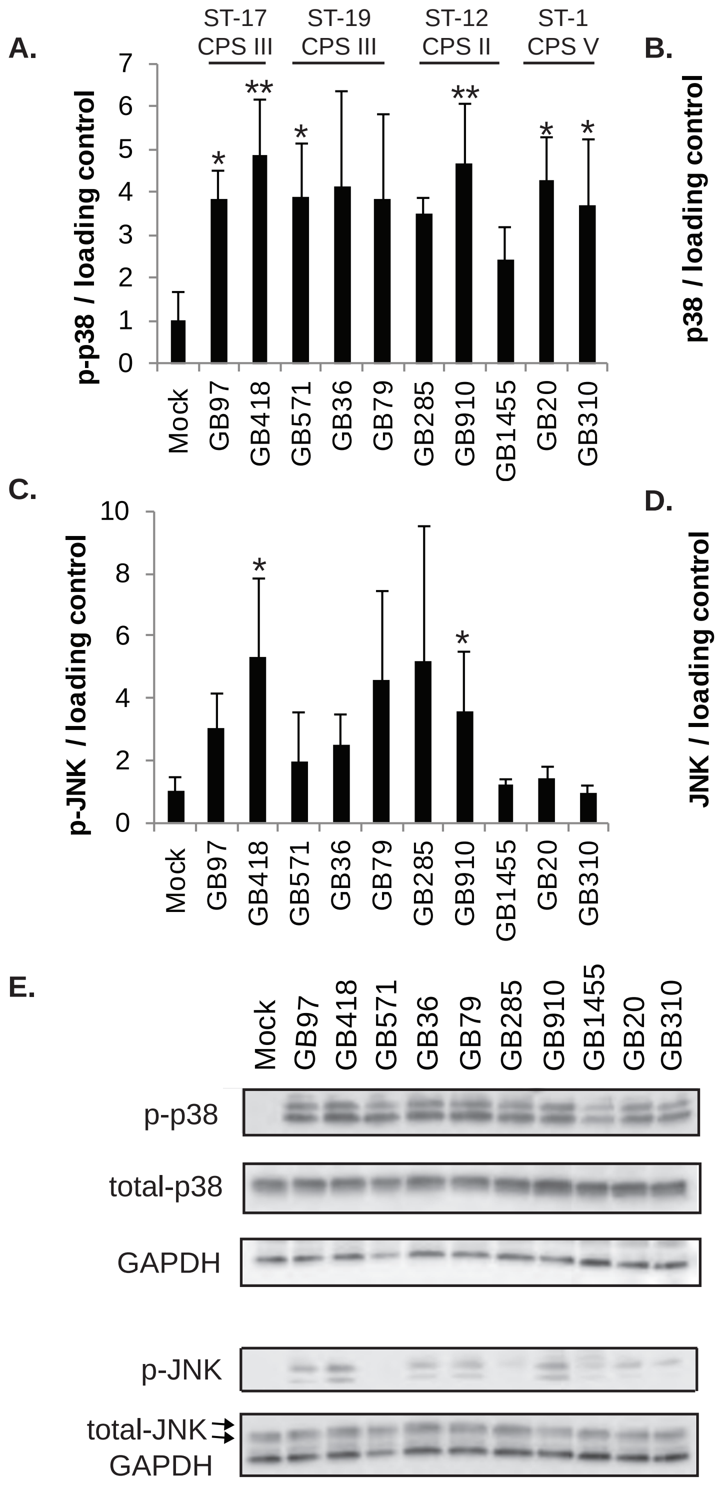

A.
ST-17 CPS III
ST-19 CPS III
ST-12 CPS II
ST-1 CPS V
p-p38 / loading control
Mock
GB97
GB418
GB571
GB36
GB79
GB285
GB910
GB1455
GB20
GB310
B.
p38 / loading control
C.
p-JNK / loading control
D.
JNK / loading control
E.
p-p38
total-p38
GAPDH
p-JNK
total-JNK
GAPDH

Figures S3-S6. Representative images of Nuclear Factor kappa B activation by immunofluorescence microscopy. THP-1 macrophages were infected with GBS at an MOI of 10 for one hour, washed, and treated with antibiotics for an additional hour prior to fixation, nuclear staining (DAPI) and detection of NFκB p65 (Alexafluor488) by immunofluorescence microscopy. Images were used to determine percent NFκB nuclear localization by comparing the number of cells with positive nuclear localization (Alexafluor488; shown in green) to the total cell number in a given field (DAPI; shown in blue) using ImageJ. Results were obtained from three independent biological replicates for each of the 15 GBS strains analyzed, which are grouped according to ST (Fig. S1 shows ST-17 strains, Fig. S2 shows ST-19 strains, Fig. S3 shows ST-12 strains, and Fig. S4 shows ST-1 strains). For each biological replicate, at least three separate fields were captured to obtain data from a minimum of 2500 cells per condition. Representative images from each strain visualizing NFκB (Alexafluor488; shown in green) and the nucleus (DAPI; shown in blue) are shown.

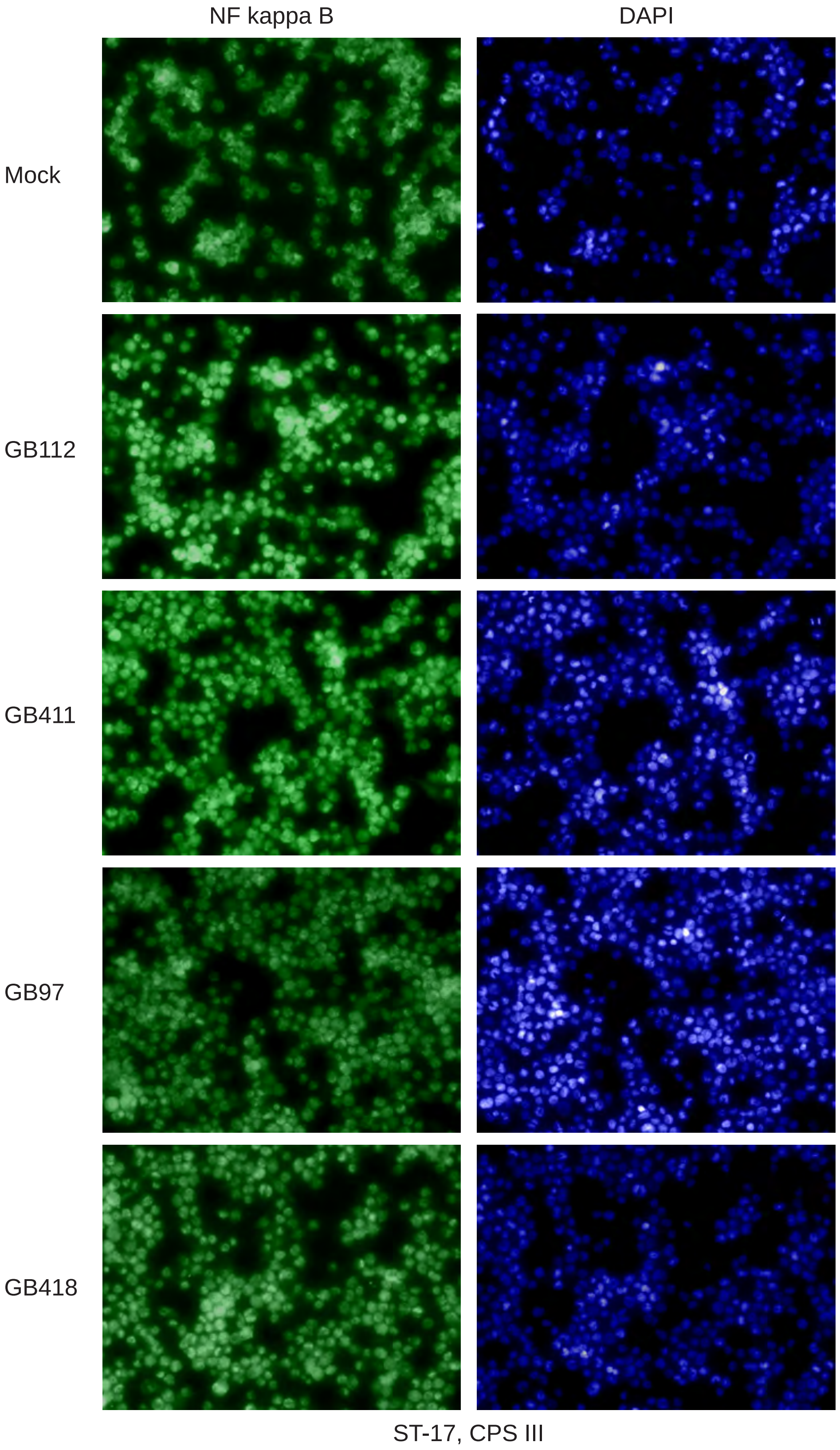
NF kappa B
DAPI
Mock
GB112
GB411
GB97
GB418
ST-17, CPS III

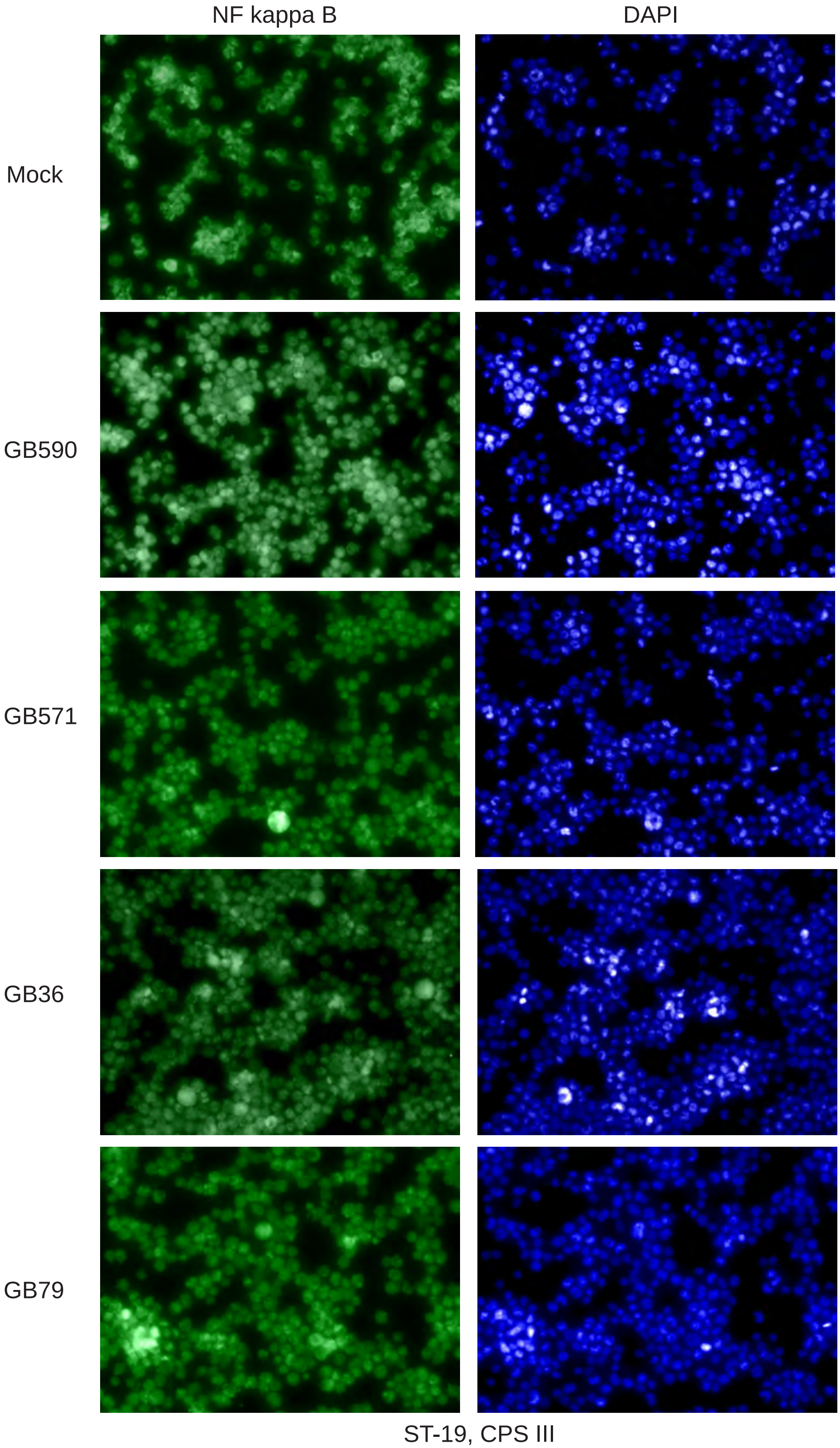
NF kappa B
DAPI
Mock
GB590
GB571
GB36
GB79
ST-19, CPS III

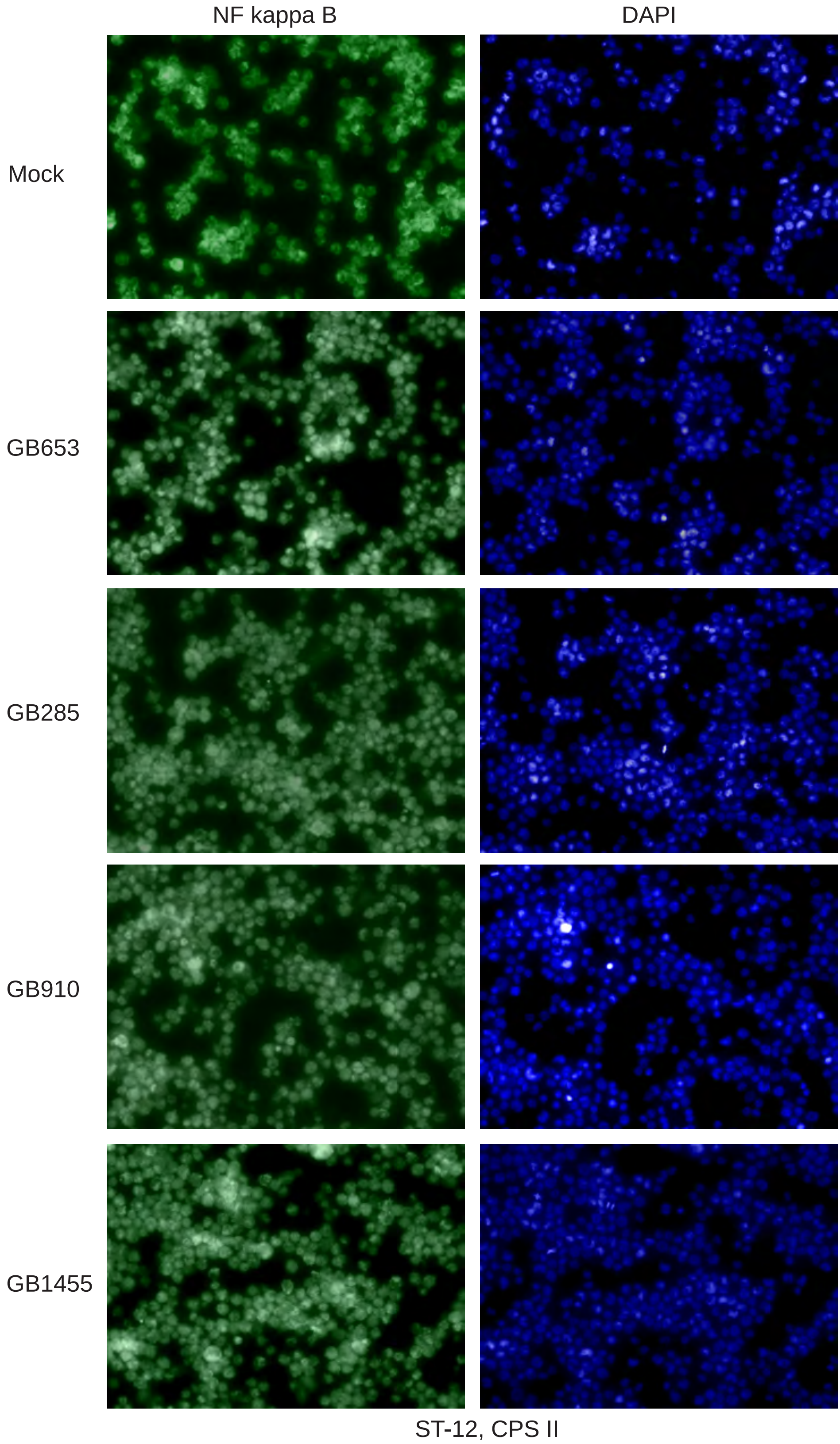
NF kappa B
DAPI
Mock
GB653
GB285
GB910
GB1455
ST-12, CPS II

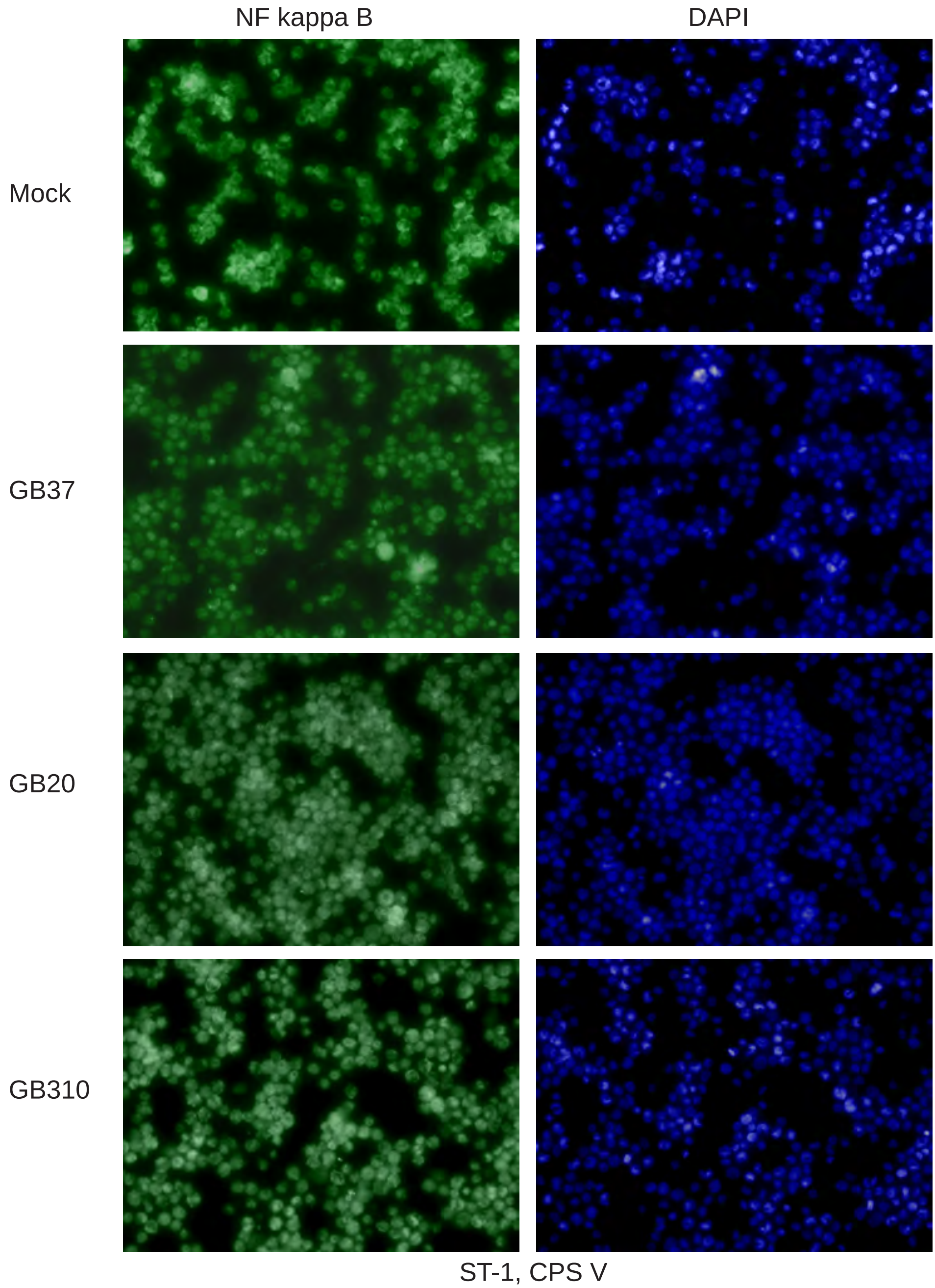
NF kappa B
DAPI
Mock
GB37
GB20
GB310
ST-1, CPS V
