# Supplementary material for: Distinct Group B Streptococcus Sequence and Capsule Types Differentially Impact Macrophage Stress and Inflammatory Signaling Responses
Source: Infect Immun. 2021 Apr 16;89(5):e00647-20. doi: 10.1128/IAI.00647-20 (PMC8091095; doi:10.1128/IAI.00647-20)
Supplement: Supplemental file 3 [file IAI.00647-20-s0003.pdf]

Table S3. Summary of Significant Comparisons Between All Strains

Final Inoculum for All Strains	
Tukey's test pairing:	Significance
GB590 vs. GB653	**
GB590 vs. GB20	**
GB36 vs. GB653	**
GB36 vs. GB20	**
GB79 vs. GB653	*
GB79 vs. GB20	*

Survival at 1hr for All Strains	
Tukey's test pairing:	Significance
GB112 vs. GB418	****
GB112 vs. GB590	***
GB112 vs. GB571	****
GB112 vs. GB36	****
GB112 vs. GB79	****
GB112 vs. GB653	****
GB112 vs. GB285	****
GB112 vs. GB910	****
GB112 vs. GB1455	****
GB112 vs. GB37	****
GB411 vs. GB97	**
GB411 vs. GB418	****
GB411 vs. GB571	**
GB411 vs. GB653	****
GB411 vs. GB285	****
GB411 vs. GB910	****
GB411 vs. GB1455	****
GB411 vs. GB37	*
GB97 vs. GB590	****
GB97 vs. GB571	****
GB97 vs. GB36	****
GB97 vs. GB79	****
GB97 vs. GB653	****
GB97 vs. GB285	****
GB97 vs. GB910	****
GB97 vs. GB1455	****
GB97 vs. GB37	****
GB418 vs. GB590	****
GB418 vs. GB571	****
GB418 vs. GB36	****
GB418 vs. GB79	****
GB418 vs. GB653	****
GB418 vs. GB285	****
GB418 vs. GB910	****
GB418 vs. GB1455	****
GB418 vs. GB37	****
GB418 vs. GB20	***
GB418 vs. GB310	***
GB590 vs. GB653	*
GB590 vs. GB285	*
GB590 vs. GB910	*
GB590 vs. GB1455	*
GB590 vs. GB20	**
GB590 vs. GB310	**
GB571 vs. GB20	****
GB571 vs. GB310	****
GB36 vs. GB20	***
GB36 vs. GB310	***
GB79 vs. GB20	***
GB79 vs. GB310	****
GB653 vs. GB20	****
GB653 vs. GB310	****
GB285 vs. GB20	****
GB285 vs. GB310	****
GB910 vs. GB20	****
GB910 vs. GB310	****
GB1455 vs. GB20	****
GB1455 vs. GB310	****
GB37 vs. GB20	****
GB37 vs. GB310	****

Survival at 24hrs for All Strains	
Tukey's test pairing:	Significance
GB112 vs. GB97	****
GB112 vs. GB590	****
GB112 vs. GB571	****
GB112 vs. GB36	****
GB112 vs. GB79	****
GB112 vs. GB653	****
GB112 vs. GB285	****
GB112 vs. GB910	****
GB112 vs. GB1455	****
GB112 vs. GB37	****
GB112 vs. GB20	****
GB112 vs. GB310	****
GB411 vs. GB97	***
GB411 vs. GB590	**
GB411 vs. GB571	****
GB411 vs. GB36	****
GB411 vs. GB79	****
GB411 vs. GB653	****
GB411 vs. GB285	****
GB411 vs. GB910	****
GB411 vs. GB1455	****
GB411 vs. GB37	****
GB411 vs. GB20	****
GB411 vs. GB310	****
GB97 vs. GB571	****
GB97 vs. GB36	**
GB97 vs. GB79	***
GB97 vs. GB653	****
GB97 vs. GB285	****
GB97 vs. GB910	****
GB97 vs. GB1455	****
GB97 vs. GB20	**
GB97 vs. GB310	*
GB418 vs. GB571	****
GB418 vs. GB36	****
GB418 vs. GB79	****
GB418 vs. GB653	****
GB418 vs. GB285	****
GB418 vs. GB910	****
GB418 vs. GB1455	****
GB418 vs. GB37	****
GB418 vs. GB20	****
GB418 vs. GB310	****
GB590 vs. GB571	****
GB590 vs. GB36	****
GB590 vs. GB79	****
GB590 vs. GB653	****
GB590 vs. GB285	****
GB590 vs. GB910	****
GB590 vs. GB1455	****
GB590 vs. GB20	****
GB590 vs. GB310	**
GB571 vs. GB37	*
GB653 vs. GB37	***
GB285 vs. GB37	***
GB910 vs. GB37	***
GB1455 vs. GB37	***
