# Supplementary material for: Distinct Group B Streptococcus Sequence and Capsule Types Differentially Impact Macrophage Stress and Inflammatory Signaling Responses
Source: Infect Immun. 2021 Apr 16;89(5):e00647-20. doi: 10.1128/IAI.00647-20 (PMC8091095; doi:10.1128/IAI.00647-20)
Supplement: Supplemental file 1 [file IAI.00647-20-s0001.pdf]

Table S1: THP-1 Post-Infection Array Analysis with Fold Changes Compared to Mock Infection and Annotated Functions

Target Protein Name	Phospho Site (Human)	Full Target Protein Name	Fold Ch. GB112	Fold Ch. GB411	Fold Ch. GB590	Fold Ch. GB653	Fold Ch. GB37	Function (Summarized from Uniprot Database)	Phosphosite Information (Kinexus PhosphoNET Database)
A6	Y309	Twinfilin-1	-0.39	-0.33	-0.85	-0.30	-1.04	Actin-binding protein involved in motile and morphological processes. Inhibits actin polymerization, likely by sequestering G-actin. By capping the barbed ends of filaments, it also regulates motility. Seems to play an important role in clathrin-mediated endocytosis and distribution of endocytic organelles.	No data available.
ACK1	Y518	Activated CDC42 kinase 1 (TNK2)	-0.66	-0.58	-1.01	-0.57	-1.07	Non-receptor tyrosine-protein and serine/threonine-protein kinase that is implicated in cell spreading and migration, cell survival, cell growth and proliferation. Transduces extracellular signals to cytosolic and nuclear effectors. Implicated in trafficking and clathrin-mediated endocytosis through binding to EGFR and clathrin. Regulates ligand-induced degradation of EGFR, thereby contributing to the accumulation of EGFR at the limiting membrane of early endosomes. Downstream effector of CDC42 which mediates CDC42-dependent cell migration.	Stimulates phosphotransferase activity. Y518 is phosphorylated by the following protein kinases in vitro: ACK1 (TNK2).
ACTA1	Pan-specific	ACTA1 (Alpha-actin)	0.71	8.38	2.06	4.25	-0.24	Actins are highly conserved proteins that are involved in various types of cell motility and are ubiquitously expressed in eukaryotic cells.	NA
AKT2 (PKBb)	Pan-specific	RAC-beta serine/threonine-protein kinase	-0.45	-0.44	-0.97	-0.44	-1.01	Serine/threonine-protein kinase which regulates many processes including metabolism, proliferation, cell survival, growth and angiogenesis. This is mediated through serine and/or threonine phosphorylation of a range of downstream substrates. Over 100 substrate candidates have been reported.	NA
AMPKa2	S377	5'-AMP-activated protein kinase catalytic subunit alpha-2 (PRKAA2)	-0.50	-0.43	-0.77	-0.48	-0.99	Catalytic subunit of AMPK, an energy sensor protein kinase that plays a key role in regulating cellular energy metabolism. In response to reduction of intracellular ATP levels, AMPK activates energy-producing pathways and inhibits energy-consuming processes. Also acts as a regulator of cellular polarity by remodeling the actin cytoskeleton and activating myosin.	No data available. Other sites are phosphoryated byLKB1 (STK11).
ANKRD3	S438	Ankyrin repeat domain protein-serine kinase 3 (RIPK4, DIK)	-0.33	-0.59	-1.04	-0.69	-1.09	Involved in stratified epithelial development. It is a direct transcriptional target of TP63. Plays a role in NF-kappa-B activation.	Within major catalytic domain; predicted to increase phospho-transferase activity.

ANXA1	Y207	Annexin A1	-0.57	-0.61	-0.92	-0.54	-1.13	Plays important roles in the innate immune response as an effector of glucocorticoid-mediated responses and as a negative regulator of the inflammatory process. Promotes resolution of inflammation and wound healing. Promotes chemotaxis of granulocytes and monocytes via activation of the formyl peptide receptors. Contributes to adaptive immune response by enhancing signaling cascades that are triggered by T-cell activation, regulates differentiation and proliferation of activated T-cells. Promotes the differentiation of T-cells into Th1 cells and negatively regulates differentiation into Th2s.	No data available.
ANXA2	Y238	Annexin A2	-0.78	-0.30	-0.95	-0.42	-1.02	Calcium-regulated membrane-binding protein whose affinity for calcium is greatly enhanced by anionic phospholipids. May be involved in heat-stress response.	No data available.
APP	T743	Amyloid beta A4 protein	-0.37	0.06	-0.30	-0.31	-1.07	Functions as a cell surface receptor and performs physiological functions on the surface of neurons relevant to neurite growth, neuronal adhesion and axonogenesis. Involved in cell mobility and transcription regulation through protein-protein interactions.	T743 is phosphorylated by CDK1 (CDC2), GSK3b, JNK1 (MAPK8), JNK2 (MAPK9), JNK3 (MAPK10).
Arrestin b	Pan-specific	Arrestin beta 1 (ARRB1)	1.59	1.23	2.19	0.71	0.47	Functions in regulating agonist-mediated GPCR signaling by mediating both receptor desensitization and resensitization processes. Targets many receptors for internalization by acting as an endocytic adapter (CLASPs, clathrin-associated sorting proteins) and recruiting the GPRCs to the adapter protein 2 complex 2 (AP-2) in clathrin-coated pits (CCPs). Can also play a role in MAPK, AKT and NFkB signaling.	NA
ASK1	S1046	Apoptosis signal regulating protein-serine kinase 1 (MAP3K5)	-0.52	0.39	-0.29	-0.60	-1.10	Serine/threonine kinase which acts as an essential component of the MAPK pathway in response to changes in the environment to determine cell fates such as differentiation and survival. Regulates apoptosis through mitochondria-dependent caspase activation, and is required for the innate immune response. Mediates signal transduction of various stressors such as oxidative stress as receptor-mediated inflammatory signals (i.e. TNF and LPS). Acts as an upstream activator of the MKK/JNK and p38 MAPK signal transduction cascades through the phosphorylation and activation of MAP2Ks (MAP2K4/SEK1, MAP2K3/MKK3, MAP2K6/MKK6 and MAP2K7/MKK7).	No data available.

ATF2	S112	Activating transcription factor 2 (CRE-BP1)	-0.43	0.24	-0.67	-0.25	-0.99	Transcriptional activator for genes regulating anti-apoptotic signaling, cell growth, and DNA damage response. Binds to CRE (cAMP response element) consensus sequences or to AP-1 (activator protein 1) consensus sequences. In the cytoplasm, interacts with and perturbs HK1- and VDAC1-containing complexes at the mitochondrial outer membrane, thereby impairing mitochondrial membrane potential, inducing mitochondrial leakage and promoting cell death.	No data available. Other sites are phosphoryated by ERK, JNK or p38 and stimulate transcriptional activity.
AurKB (Aurora B)	S227	Aurora Kinase B (serine/threonine protein kinase 12), (AIM-1)	1.00	0.13	0.36	0.70	0.40	Serine/threonine-protein kinase component of the chromosomal passenger complex (CPC), a complex that acts as a key regulator of mitosis that ensures correct chromosome alignment and segregation as well as chromatin-induced microtubule stabilization and spindle assembly. Involved in the bipolar attachment of spindle microtubules to kinetochores and is a key regulator for the onset of cytokinesis during mitosis.	No data available. Other sites stimulate phosphotransferase activity.
AurKB (Aurora B)	Pan-specific	Aurora Kinase B (serine/threonine protein kinase 12), (AIM-1)	1.81	0.49	-0.01	0.13	-0.16	See above.	NA
BCR	Y177	Breakpoint cluster region protein	-0.55	1.09	-0.26	0.21	-0.50	GTPase-activating protein for RAC1, RAC2, and CDC42. The C-terminus is a GTPase-activating protein (GAP) domain which stimulates GTP hydrolysis by RAC1, RAC2 and CDC42 - this accelerates the intrinsic rate of GTP hydrolysis, leading to down-regulation of the active GTP-bound form. The central Dbl homology (DH) domain functions as guanine nucleotide exchange factor (GEF) that modulates the GTPases CDC42, RHOA and RAC1 - this promotes the conversion of CDC42, RHOA and RAC1 from the GDP-bound to the GTP-bound form. The amino terminus contains an intrinsic kinase activity. Regulates macrophage functions such as CSF1-directed motility and phagocytosis through the modulation of RAC1 activity. Plays a major role as a RHOA GEF in focal adhesion formation.	Induces interaction with Gab2, Grb2 and SOS1, and inhibits interaction with Hck. Y177 is phosphorylated by Abl1, Bcr, Fes, Fyn, Hck, Lyn.
Bmx	Y40	Bone marrow X protein-tyrosine kinase (Etk)	-0.08	1.53	0.04	-0.37	-0.72	Non-receptor tyrosine kinase that contributes to the regulation of actin reorganization, cell migration, cell proliferation and survival, cell adhesion, and apoptosis. Participates in signal transduction stimulated by growth factor receptors, cytokine receptors, G-protein coupled receptors, antigen receptors and integrins.	Stimulates phosphotransferase activity and regulates cell motility, cytoskeletal reorganization, intracellular location, and induces interaction with FAK.
BRCA1	S1497	Breast cancer type 1 susceptibility protein	1.04	0.69	0.00	0.08	-0.54	E3 ubiquitin-protein ligase that specifically mediates the formation of 'Lys-6'-linked polyubiquitin chains and plays a central role in DNA repair by facilitating cellular responses to DNA damage.	S1497 is phosphorylated by the following protein kinases in vitro: ATM, CDK2

BTK	Pan-specific	Bruton's agammaglobulinemia tyrosine kinase	-0.69	0.20	-0.89	-0.60	-1.06	Non-receptor tyrosine kinase that regulates apoptosis and is required for B lymphocyte development, differentiation and signaling. After BCR engagement and activation, phosphorylates PLCG2, igniting the downstream signaling pathway through calcium mobilization, followed by activation of PKC family members.	NA
Catenin a	S641	Catenin (cadherin-associated protein) alpha (CTNNA1)	-0.35	0.70	0.72	1.89	0.22	Associates with the cytoplasmic domain of a variety of cadherins. The association of catenins to cadherins produces a complex which is linked to the actin filament network, and which seems to be of primary importance for cadherins cell-adhesion properties. Can associate with both E- and N-cadherins.	No data available.
Catenin b	Pan-specific	Catenin (cadherin-associated protein) beta 1 (CTNNB1)	-0.87	7.13	-0.33	-0.42	-0.12	Key downstream component of the canonical Wnt signaling pathway. In the absence of Wnt, forms a complex with AXIN1, AXIN2, APC, CSNK1A1 and GSK3B that promotes phosphorylation and ubiquitination of CTNNB1 via BTRC and its subsequent degradation by the proteasome. In the presence of Wnt, it is not ubiquitinated and accumulates in the nucleus, where it acts as a coactivator for TFs of the TCF/LEF family to activate Wnt responsive genes. Involved in the regulation of cell adhesion.	NA
Catenin b	Pan-specific	Catenin (cadherin-associated protein) beta 1 (CTNNB1)	0.29	-0.33	-0.98	-0.59	-0.72	See above.	NA
Caveolin 1	Y14	Caveolin 1 (CAV1)	0.09	0.60	0.21	1.50	0.02	May act as a scaffolding protein within caveolar membranes. Interacts directly with G-protein alpha subunits and functionally regulates their activity. Involved in the costimulatory signal essential for T-cell receptor (TCR)-mediated T-cell activation. Its binding to DPP4 induces T-cell proliferation and NF-kappa-B activation in a T-cell receptor/CD3-dependent manner. Recruits CTNNB1 to caveolar membranes; regulates CTNNB1-mediated signaling through the Wnt pathway. Negatively regulates TGFB1-mediated activation of SMAD2/3 via internalization of TGFBR1 from membrane rafts leading to its subsequent degradation.	Y14 is phosphorylated by Fyn, InsR, Src.
Caveolin 2	Pan-specific	Caveolin 2 (CAV2)	1.86	0.80	1.04	-0.39	-0.28	May act as a scaffolding protein within caveolar membranes. Interacts directly with G-protein alpha subunits and can functionally regulate their activity. Acts as an accessory protein in conjunction with CAV1 in targeting to lipid rafts and driving caveolae formation. The phospho-Ser-36 form modulates mitosis in endothelial cells. Positive regulator of cellular mitogenesis of the MAPK pathway. Required for insulin-stimulated activation of MAPK1 and STAT3, and subsequent regulation of cell cycle progression.	NA

CBL	Y700	Signal transduction protein CBL	0.33	1.31	1.27	0.80	0.81	Adapter protein and E3 ubiquitin-protein ligase that functions as a negative regulator of many signaling pathways that are triggered by activation of cell surface receptors by targeting proteins for degradation by the proteasome. Recognizes activated receptor tyrosine kinases, including KIT, FLT1, FGFR1, FGFR2, PDGFRA, PDGFRB, EGFR, CSF1R, EPHA8 and KDR and terminates signaling. Recognizes membrane-bound HCK, SRC and other kinases of the SRC family and mediates their ubiquitination and degradation. Participates in signal transduction in hematopoietic cells. Regulates osteoblast differentiation and apoptosis.	Y700 is phosphorylated by the following protein kinases in vitro: Abl1, Fyn, InsR, Lck, Src, Syk, Yes
CDK1	T161	Cyclin-dependent protein-serine kinase 1 (CDC2)	0.31	-1.23	-0.30	-1.33	-0.65	Plays a key role in the control of the eukaryotic cell cycle by modulating the centrosome cycle and mitotic onset; promotes G2-M transition, and regulates G1 progress and G1-S transition via association with multiple interphase cyclins. Required for entry into S-phase and mitosis.	Stimulates phosphotransferase activity and protein interaction. Phosphorylation regulates cell cycle progression, cell growth and molecular association. T161 is phosphorylated by CDK7.
CDK1/2	Y15	Cyclin-dependent protein-serine kinase 1/2 (CDC2)	0.13	1.94	1.01	1.04	0.08	See above for CDK1. For CDK2: Serine/threonine-protein kinase involved in the control of the cell cycle; essential for meiosis, but dispensable for mitosis. Phosphorylates CTNNB1, USP37, p53/TP53, NPM1, CDK7, RB1, BRCA2, MYC, NPAT, EZH2. Triggers duplication of centrosomes and DNA. Acts at the G1-S transition to promote the E2F transcriptional program and the initiation of DNA synthesis, and modulates G2 progression; controls the timing of entry into mitosis/meiosis by controlling the activation of cyclin B/CDK1 by phosphorylation. Orchestrates the balance between cellular proliferation, cell death, and DNA repair.	Inhibits phosphotransferase activity, and regulates cell cycle progression and apoptosis. Y15 is phosphorylated by CDK1 (CDC2), Chk1 (CHEK1), MYT1 (PKMYT1), Src, Wee1.
CDK1/2	Y15	Cyclin-dependent protein-serine kinase 1/2 (CDC2)	0.05	1.18	1.09	1.71	0.33	See above.	Inhibits phosphotransferase activity, and regulates cell cycle progression and apoptosis. Y15 is phosphorylated by CDK1 (CDC2), Chk1 (CHEK1), MYT1 (PKMYT1), Src, Wee1.
CDK1/2	Pan-specific	Cyclin-dependent protein-serine kinase 1/2 (CDC2)	1.87	0.65	0.96	0.90	1.49	See above.	NA
CDK2	Pan-specific	Cyclin-dependent protein-serine kinase 2	-0.49	-1.10	-1.08	-0.75	-1.10	See above.	NA
CDK2	Pan-specific	Cyclin-dependent protein-serine kinase 2	-0.49	-1.10	-1.08	-0.75	-1.10	See above.	NA

CDK6	Y13	Cyclin-dependent protein-serine kinase 6	0.07	1.27	0.67	0.99	0.52	Serine/threonine-protein kinase involved in the control of the cell cycle and differentiation; promotes G1/S transition. Phosphorylates pRB/RB1 and NPM1. Interacts with D-type G1 cyclins during interphase at G1 to form a pRB/RB1 kinase and controls the entrance into the cell cycle. Involved in initiation and maintenance of cell cycle exit during cell differentiation; prevents cell proliferation in most cell types and negatively regulates differentiation. Required for thymocyte development. Promotes production of new neurons and changes in the actin cytoskeleton including loss of stress fibers, and enhanced motility during cell differentiation.	No data available.
CDK7	Pan-specific	Cyclin-dependent protein-serine kinase 7	1.36	-0.60	-0.31	-0.44	-0.24	Serine/threonine kinase involved in cell cycle control and in RNA polymerase II-mediated RNA transcription. Required for both activation and complex formation of CDK1/cyclin-B during G2-M transition, and for activation of CDK2/cyclins during G1-S transition. CDK7 is the catalytic subunit of the CDK-activating kinase (CAK) complex. Upon DNA damage, triggers p53/TP53 activation (phosphorylation), allowing cell cycle arrest and recovery or apoptosis.	NA
CDK9	Pan-specific	Cyclin-dependent protein-serine kinase 9	0.05	-0.14	-1.24	-0.49	-1.03	Protein kinase involved in the regulation of transcription, cotranscriptional histone modification, and mRNA processing and export. . Member of the CDK9/cyclin-T complex (aka P-TEFb), which facilitates the transition from abortive to productive elongation by phosphorylating RNA polymerase II (RNAP II). Regulates cytokine inducible transcription networks by facilitating promoter recognition of target transcription factors (e.g. TNF-inducible RELA/p65 activation and IL-6-inducible STAT3 signaling). Promotes RNA synthesis for cell growth and differentiation. The CDK9/cyclin-K complex is required for genome integrity maintenance, by promoting cell cycle recovery from replication arrest and limiting ss-DNA in response to replication stress.	NA
CDK10	T196	Cyclin-dependent protein-serine kinase 10 (PISSLRE)	0.43	-0.24	-0.54	-1.05	-0.83	Cyclin-dependent kinase that phosphorylates the transcription factor ETS2 (in vitro) and positively controls its proteasomal degradation (in cells). Involved in the regulation of actin cytoskeleton organization through the phosphorylation of actin dynamics regulators such as PKN2. Also a negative regulator of ciliogenesis through phosphorylation of PKN2 and promotion of RhoA signaling.	Stimulates phosphotransferase activity.

CDK11A	T583	Cell division cycle 2-like 2 protein kinase (Cdc2L2)	0.43	-0.61	-0.65	-1.25	-0.71	Appears to play multiple roles in cell cycle progression, cytokinesis and apoptosis. The p110 isoforms have been suggested to be involved in pre-mRNA splicing, potentially by phosphorylating the splicing protein SFRS7. The p58 isoform may act as a negative regulator of normal cell cycle progression.	No data available.
CDK12	S383+S385	Cell division protein kinase 12 (Cdc2L7)	0.05	-0.75	-0.51	-1.24	-1.08	Cyclin-dependent kinase that phosphorylates the C-terminal domain (CTD) of the large subunit of RNA polymerase II (POLR2A), thereby acting as a key regulator of transcription elongation. Regulates the expression of genes involved in DNA repair and is required for the maintenance of genomic stability. Preferentially phosphorylates 'Ser-5' in CTD repeats that are already phosphorylated at 'Ser-7', but can also phosphorylate 'Ser-2'. Required for RNA splicing, possibly by phosphorylating SRSF1/SF2. Involved in regulation of MAP kinase activity, possibly leading to affect the response to estrogen inhibitors.	No data available.
CDKL5	Y171	Cyclin-dependent kinase-like 5 (STK9)	0.02	-0.65	-0.47	-1.23	-0.39	Mediates phosphorylation of MECP2. May regulate ciliogenesis.	Stimulates phosphotransferase activity. Y171 is phosphorylated by the following protein kinases in vitro: CDKL5 (STK9).
CHK1	S280	Checkpoint protein-serine kinase 1 (CHEK1)	0.29	-0.72	-0.99	-0.69	-0.77	Serine/threonine-protein kinase which is required for checkpoint-mediated cell cycle arrest and activation of DNA repair in response to the presence of DNA damage or unreplicated DNA. May also negatively regulate cell cycle progression during unperturbed cell cycles.	Inhibits phosphotransferase activity. Phosphorylation regulates cell cycle progression, intracellular location, protein degradation by promoting mono and/or diubiquitination and molecular association. S280 is phosphorylated by Akt1 (PKBa).
CLK1	S337	Dual specificity protein kinase CLK1	1.76	0.74	0.87	0.92	0.97	Dual specificity kinase acting on both serine/threonine and tyrosine-containing substrates. Phosphorylates serine- and arginine-rich (SR) proteins of the spliceosomal complex and may be a constituent of a network of regulatory mechanisms that enable SR proteins to control RNA splicing. Phosphorylates: SRSF1, SRSF3 and PTPN1.	Predicted to be stimulatory for phosphotransferase activity.
Cofilin 1	Pan-specific	Cofilin 1 (CFL1)	1.53	-0.23	1.27	-0.27	0.01	Binds to F-actin and exhibits pH-sensitive F-actin depolymerizing activity. Important for normal progress through mitosis and normal cytokinesis. Plays a role in the regulation of cell morphology and cytoskeletal organization. Required for the up-regulation of atypical chemokine receptor ACKR2 from endosomal compartment to cell membrane, increasing its efficiency in chemokine uptake and degradation.	NA

Cofilin 1	S3	Cofilin 1 (CFL1)	45.59	21.30	35.72	1.93	1.96	See above.	Phosphorylation reduces binding to F-actin and G-actin, and nuclear localization. 14-3-3 binds to phosphorylated Cofilin and prevents its dephosphorylation by phosphatase to prolong its functional activity. S3 is phosphorylated by LIM kinase 1, LIM kinase 2.
Connexin 43	S368	Gap junction alpha-1 protein (Cx43, GJA1)	5.83	1.60	2.61	2.03	2.09	Gap junction protein that acts as a regulator of bladder capacity and communication in the ventricles. A gap junction consists of a cluster of closely packed pairs of transmembrane channels, the connexons, through which materials of low MW diffuse from one cell to a neighboring cell. May play a critical role in the physiology of hearing by participating in the recycling of potassium to the cochlear endolymph and in cell growth inhibition.	S368 is phosphorylated by PKACa (PRKACA), PKCa (PRKCA), PKCe (PRKCE).
COT	Pan-specific	Osaka thyroid oncogene protein-serine kinase (TPL2) (MAP3K8)	0.94	3.04	1.07	1.80	1.99	Required for LPS-induced, TLR4-mediated activation of the MAPK/ERK pathway in macrophages, thus being critical for production of the proinflammatory cytokine production (i.e. TNF). Involved in the regulation of T-helper cell differentiation and IFNG expression. Involved in mediating host resistance to bacterial infection through negative regulation of type I interferon (IFN) production. Activates MAPK/ERK pathway in response to IL1 in an IRAK1-independent manner, leading to up-regulation of IL8 and CCL4. Transduces CD40 and TNFRSF1A signals that activate ERK in B-cells and macrophages. May also play a role in the transduction of TNF signals that activate JNK and NF-kappa-B in some cell types. Plays a role in the cell cycle.	NA
Crystallin aB	Pan-specific	Crystallin alpha B (heat-shock 20 kDa like-protein) (HspB5; CRYA2; CRYAB)	1.61	-0.38	-0.35	-0.50	-0.04	Has chaperone-like activity, preventing aggregation of various proteins under a wide range of stress conditions.	NA
CSK	Pan-specific	C-terminus of Src tyrosine kinase	1.55	0.91	0.74	0.04	-0.20	Non-receptor tyrosine-protein kinase that plays an important role in the regulation of cell growth, differentiation, migration and immune response. Phosphorylates Src-family kinases (SFKs) including LCK, SRC, HCK, FYN, LYN or YES1.	NA
CSK	Pan-specific	C-terminus of Src tyrosine kinase	-1.06	-0.82	-1.46	-0.72	-1.09	See above.	NA
Cyclin B1	S147	Cyclin B1 (CCNB1)	0.12	1.25	-0.60	0.06	-0.51	Essential for the control of the cell cycle at the G2/M (mitosis) transition.	S147 is phosphorylated by the following protein kinases in vitro: Plk1 (PLK).

Cyclin E1	T395	Cyclin E1 (CCNE1)	0.06	1.34	-0.13	1.00	-0.31	Essential for the control of the cell cycle at the G1/S (start) transition.	T395 is phosphorylated by the following protein kinases in vitro: CDK2, GSK3a, GSK3b.
DAPK3	S269	Death-associated protein kinase 3 (DLK, MAP3K12)	1.07	-0.57	-0.51	-0.14	-0.27	Serine/threonine kinase involved in the regulation of apoptosis, autophagy, transcription, translation and actin cytoskeleton reorganization. Also regulates smooth muscle contraction. Regulates caspase-dependent apoptotic cell death and caspase-independent autophagic cell death. Regulates myosin phosphorylation in non-muscle cells and in smooth muscle (via MYL12B and MYL9 phosphorylation or via inhibition of smooth muscle myosin phosphatase). Enhances muscle responsiveness to Ca2+ to promote a contractile state. Phosphorylates MYL12B in non-muscle cells leading to reorganization of actin cytoskeleton. Involved in actin filament focal adhesion dynamics modulated by RhoD. Positively regulates canonical Wnt/beta-catenin signaling through interaction with NLK and TCF7L2. Involved in regulation of cell cycle progression and cell proliferation.	No data available.
DDR2	Y736	Discoidin domain-containing receptor 2 (Tyro10)	0.23	-0.87	-0.97	-0.53	-0.77	Tyrosine kinase that functions as cell surface receptor for fibrillar collagen and regulates cell differentiation, remodeling of the extracellular matrix, cell migration and cell proliferation. Required for normal bone development. Regulates osteoblast differentiation and chondrocyte maturation via a signaling pathway that involves MAPKs and leads to the activation of the transcription factor RUNX2. Regulates remodeling of the extracellular matrix by up-regulation of the collagenases MMP1, MMP2 and MMP13, and thereby facilitates cell migration and tumor cell invasion. Promotes fibroblast migration and proliferation, and thereby contributes to cutaneous wound healing.	Stimulates phosphotransferase activity. Y736 is phosphorylated by Src.
DNAPK	T2609	DNA-activated protein-serine kinase (PRKDC)	2.41	-0.56	-0.78	-0.72	-0.56	Serine/threonine-protein kinase that acts as a molecular sensor for DNA damage. Involved in DNA non-homologous end joining (NHEJ) required for double-strand break (DSB) repair and V(D)J recombination. Must be bound to DNA to express its catalytic properties.	Stimulates phosphotransferase activity and regulates cell growth, chromatin reorganization, and intracellular location. T2609 is phosphorylated by the following protein kinases in vitro: ATM, ATR, DNAPK (PRKDC). T2609 is dephos-phorylated by PPP5C.

DYRK2	Y382	Dual specificity tyrosine-phosphorylation-regulated kinase 2	1.76	0.33	0.08	0.20	0.67	Serine/threonine-protein kinase involved in the regulation of the mitotic cell cycle, cell proliferation, apoptosis, organization of the cytoskeleton and neurite outgrowth. Functions in part via its role in ubiquitin-dependent proteasomal protein degradation. Functions downstream of ATM and phosphorylates p53/TP53 at 'Ser-46', and thereby contributes to the induction of apoptosis in response to DNA damage.	Predicted to be stimulatory for phosphotransferase activity.
eEF1A1	Y141	Elongation factor 1-alpha 1	1.46	-0.62	-0.41	-0.40	-0.62	Promotes the GTP-dependent binding of aminoacyl-tRNA to the A-site of ribosomes during protein biosynthesis. With PARP1 and TXK, forms a complex that acts as a T helper 1 (Th1) cell-specific transcription factor and binds the promoter of IFN-gamma to directly regulate its transcription, and is thus involved importantly in Th1 cytokine production.	No data available.
EFNB2	Y316	EPH-related receptor tyrosine kinase ligand 5	-0.20	1.71	0.60	0.40	0.24	Cell surface transmembrane ligand for Eph receptors, a family of receptor tyrosine kinases which are crucial for migration, repulsion and adhesion during neuronal, vascular and epithelial development. Also binds Eph receptors residing on adjacent cells, leading to contact-dependent bidirectional signaling into neighboring cells.	No data available.
EGFR	T693	Epidermal growth factor receptor-tyrosine kinase (ErbB1)	-0.36	0.85	-1.04	1.05	-0.58	Receptor tyrosine kinase binding ligands of the EGF family and activating several signaling cascades to convert extracellular cues into appropriate cellular responses. Known ligands: EGF, TGFA/TGF-alpha, amphiregulin, epigen/EPGN, BTC/betacellulin, epiregulin/EREG and HBEGF/heparin-binding EGF. Ligand binding triggers receptor homo- and/or heterodimerization and autophosphorylation on key cytoplasmic residues. The phosphorylated receptor recruits adapter proteins like GRB2 which activate downstream signaling cascades. Activates at least 4 major pathways including the RAS-RAF-MEK-ERK, PI3 kinase-AKT, PLCgamma-PKC and STATs modules. May also activate the NF-kappa-B signaling.	Inhibits phosphotransferase activity. T693 is phosphorylated by ERK1 (MAPK3), ERK2 (MAPK1), p38a MAPK (MAPK14), PRKD1.
EGFR	Y1110	Epidermal growth factor receptor-tyrosine kinase (ErbB1)	0.38	1.52	0.49	1.03	0.19	See above.	Stimulates phosphotransferase activity, induces binding of STAT3, and regulates transcription, protein conformation, intracellular location, receptor internalization, molecular association, and ubquitination. Y1110 is phosphorylated by EGFR.

EGFR	Y1172	Epidermal growth factor receptor-tyrosine kinase (ErbB1)	0.94	1.14	0.78	0.96	0.35	See above.	Stimulates phosphotransferase activity. Phosphorylation regulates cell growth, protein degradation, receptor internalization, and molecular association. Y1172 is phosphorylated by the following protein kinases in vitro: EGFR.
EGFR	Y1172	Epidermal growth factor receptor-tyrosine kinase (ErbB1)	1.42	0.50	0.64	0.22	-0.08	See above.	See above.
EGFR	Y1172	Epidermal growth factor receptor-tyrosine kinase (ErbB1)	1.39	-0.44	-0.39	-0.31	-0.22	See above.	See above.
EGFR	Y1197	Epidermal growth factor receptor-tyrosine kinase (ErbB1)	0.08	1.78	0.11	1.39	0.11	See above.	Stimulates phospho-transferase activity. Autophosphorylation at Tyr-1197 is stimulated by methylation at Arg-1199 and enhances interaction with PTPN6. Phosphorylation regulates cell growth, intracellular location, protein degradation, receptor internalization and molecular association. Y1197 is phosphorylated by Abl1, EGFR.
EGFR	Y869	Epidermal growth factor receptor-tyrosine kinase (ErbB1)	-0.04	-0.77	-1.08	-0.67	-0.72	See above.	Stimulates phosphotransferase activity and protein interaction. Y869 is phosphorylated by Src.
eIF2a	Pan-specific	Eukaryotic translation initiation factor 2 alpha	-0.56	-0.26	-1.04	-0.39	-0.93	Functions in the early steps of protein synthesis of a small number of specific mRNAs by directing the binding of methionyl-tRNAi to 40S ribosomal subunits. In contrast to the eIF-2 complex, it binds methionyl-tRNAi to 40S subunits in a codon-dependent manner, whereas the eIF-2 complex binds methionyl-tRNAi to 40S subunits in a GTP-dependent manner.	NA
eIF4B	S422	Eukaryotic translation initiation factor 4B	0.22	1.18	0.97	1.20	0.28	Required for the binding of mRNA to ribosomes. Functions in close association with EIF4-F and EIF4-A. Binds near the 5'-terminal cap of mRNA in presence of EIF-4F and ATP. Promotes the ATPase activity and the ATP-dependent RNA unwinding activity of both EIF4-A and EIF4-F.	S422 is phosphorylated by p70S6K (RPS6KB1), RSK1 (RPS6KA2).

eIF4G	S1106	Eukaryotic translation initiation factor 4 gamma 1 (eIF4G1)	2.15	2.12	2.38	0.26	2.77	Component of the protein complex eIF4F, which is involved in the recognition of the mRNA cap, ATP-dependent unwinding of 5'-terminal secondary structure and recruitment of mRNA to the ribosome. A member of the eIF4F complex; required for ER stress-induced ATF4 mRNA translation.	No data available.
ELK1	Pan-specific	ETS domain-containing protein Elk-1	0.11	1.60	0.85	0.56	0.10	Transcription factor that binds to purine-rich DNA sequences. Forms a ternary complex with SRF and the ETS and SRF motifs of the serum response element (SRE) on the promoter region of immediate early genes such as FOS and IER2. Induces target gene transcription upon JNK pathway stimulation.	NA
EphA2	Y772	Ephrin type-A receptor 2 protein-tyrosine kinase	1.07	1.16	0.66	0.37	-0.11	Receptor tyrosine kinase which binds membrane-bound ephrin-A family ligands residing on adjacent cells, leading to contact-dependent bidirectional signaling into neighboring cells. Activated by the ligand ephrin-A1/EFNA1 regulates migration, integrin-mediated adhesion, proliferation and differentiation of cells. Regulates cell adhesion and differentiation through DSG1/desmoglein-1 and inhibition of the ERK1/ERK2 (MAPK3/MAPK1, respectively) signaling pathway. May also participate in UV radiation-induced apoptosis and have a ligand-independent stimulatory effect on chemotactic cell migration. During development, may function in distinctive aspects of pattern formation and subsequently in development of several fetal tissues. Involved in angiogenesis, in early hindbrain development and epithelial proliferation and branching morphogenesis during mammary gland development. With ephrin-A2/EFNA2 may play a role in bone remodeling through regulation of osteoclastogenesis and osteoblastogenesis.	Y772 is phosphorylated by the following protein kinases in vitro: EphA2.
EphA3	Y779	Ephrin type-A receptor 3 protein-tyrosine kinase	0.10	2.57	1.33	0.69	1.08	Receptor tyrosine kinase which binds promiscuously membrane-bound ephrin family ligands residing on adjacent cells, leading to contact-dependent bidirectional signaling into neighboring cells. Upon activation by EFNA5 regulates cell-cell adhesion, cytoskeletal organization and cell migration. Also plays a role in cardiac cell migration and differentiation, retinotectal mapping of neurons, and segregation of motor and sensory axons during neuromuscular circuit development.	Stimulates phosphotransferase activity. Phosphorylation regulates cell adhesion, cytoskeletal reorganization, and molecular association.

ER-alpha	S104	Estrogen receptor alpha (ESR1)	-0.19	1.13	0.25	-0.10	-0.33	Nuclear hormone receptor. The steroid hormones and their receptors are involved in the regulation of eukaryotic gene expression and affect cellular proliferation and differentiation in target tissues. Ligand-dependent nuclear transactivation involves either direct homodimer binding to a palindromic estrogen response element (ERE) sequence or association with other DNA-binding transcription factors, such as AP-1/c-Jun, c-Fos, ATF-2, Sp1 and Sp3, to mediate ERE-independent signaling. Decreases NF-kappa-B DNA-binding activity and transcription from the IL6 promoter and displaces RELA/p65 and associated coregulators from the promoter. Recruited to the NF-kappa-B response element of the CCL2 and IL8 promoters; displaces CREBBP.	Stimulates transcriptional activity and regulates molecular association, including inducing interactions with NCoA2, SRC-3 and Src. S104 is phosphorylated by BARK2 (GRK3, ADRBK2), CDK2, ERK1 (MAPK3), ERK2 (MAPK1), GSK3b.
ErbB2 (HER2)	Y877	ErbB2 (Neu) receptor-tyrosine kinase	1.26	1.36	0.61	0.66	0.25	Protein tyrosine kinase that is part of several cell surface receptor complexes but needs a coreceptor for ligand binding. Essential component of a neuregulin-receptor complex. GP30 is a potential ligand. Regulates outgrowth and stabilization of peripheral microtubules (MTs). Upon ERBB2 activation, the MEMO1-RHOA-DIAPH1 signaling pathway elicits the phosphorylation and inhibition of GSK3B at cell membrane. This prevents the phosphorylation of APC and CLASP2, allowing its association with the cell membrane. Membrane-bound APC allows the localization of MACF1 to the cell membrane (for microtubule capture and stabilization).	Stimulates phosphotransferase activity. Phosphorylation regulates cell growth.
ErbB3 (HER3)	Y1328	Tyrosine kinase-type cell surface receptor HER3	0.36	1.81	1.14	0.60	0.90	Tyrosine-protein kinase that plays an essential role as cell surface receptor for neuregulins. Binds to neuregulin-1 (NRG1) and is activated by it; ligand-binding increases phosphorylation on tyrosine residues and promotes its association with the p85 subunit of phosphatidylinositol 3-kinase.	No data available.
ERK5 (MAPK7)	Y221	Extracellular regulated protein-serine kinase 5 (Big MAP kinase 1 (BMK1))	1.01	-0.09	-0.32	-0.33	-0.68	Plays a role in various cellular processes such as proliferation, differentiation and cell survival. The upstream activator of MAPK7 is MAP2K5. Upon activation, it translocates to the nucleus and phosphorylates various downstream targets including MEF2C. EGF activates MAPK7 through a Ras-independent and MAP2K5-dependent pathway. May have a role in muscle cell differentiation, endothelial function and maintenance of blood vessel integrity. MAP2K5 and MAPK7 interact specifically with one another and not with MEK1/ERK1 or MEK2/ERK2 pathways. Phosphorylates SGK1 at Ser-78 and this is required for growth factor-induced cell cycle progression. Involved in the regulation of p53/TP53 by disrupting the PML-MDM2 interaction.	Stimulates phosphotransferase activity. This phosphosite is located in the kinase activation loop between catalytic subdomains VII and VIII. Phosphorylation regulates apoptosis. Y221 is phosphorylated by the following protein kinases in vitro: MEK5 (MAP2K5).

FAK (PTK2)	Pan-specific	Focal adhesion protein-tyrosine kinase	-0.89	-0.66	-0.95	-0.80	-1.04	Non-receptor tyrosine kinase that regulates cell migration, adhesion, spreading, reorganization of the actin cytoskeleton, formation and disassembly of focal adhesions and cell protrusions, cell cycle progression, cell proliferation and apoptosis. Required for early embryonic development, placenta development, embryonic angiogenesis, normal cardiomyocyte migration and proliferation, and normal heart development. Regulates axon growth and neuronal cell migration, axon branching and synapse formation; required for normal development of the nervous system. Plays a role in osteogenesis and differentiation of osteoblasts. Aids in signaling downstream of integrins, growth factor receptors, GPCRs, EPHA2, netrin receptors and LDL receptors. Forms signaling complexes with SRC and SRC family members, leading to creation of binding sites for scaffold proteins, effectors and substrates. Promotes activation of PI3K-AKT1 signaling, the MAPK pathway and RAC1. Promotes activation of GEFs and GAPs to regulate activity of Rho family GTPases. Recruits MDM2 to P53/TP53 in the nucleus, and phosphorylates ACTN1, ARHGEF7, GRB7, RET and WASL. Promotes phosphorylation of PXN, STAT1, BCAR1, GIT2, SHC1, BMX and PIK3R1.	NA
FAK (PTK2)	Y576+Y577	Focal adhesion protein-tyrosine kinase	-0.29	0.54	1.29	1.13	0.50	See above.	Stimulates phospho-transferase activity. Phosphorylation regulates cell adhesion, cell differentiation, cyto-skeletal reorganization, transcription, and alters intracellular location. Y576 is phosphorylated by FAK, Fgr, Met, Src.
FGFR2	Y656+Y657	Fibroblast growth factor receptor-tyrosine kinase 2 (BEK)	2.21	0.05	0.05	-0.13	-0.27	Tyrosine-protein kinase that acts as cell-surface receptor for fibroblast growth factors and regulates cell proliferation, differentiation, migration and apoptosis, and embryonic development. Required for normal embryonic patterning, trophoblast function, limb bud development, lung morphogenesis, osteogenesis and skin development. Regulates osteoblast differentiation, proliferation and apoptosis, and is required for normal skeleton development. Promotes cell proliferation in keratinocytes and immature osteoblasts, but promotes apoptosis in differentiated osteoblasts. Phosphorylates PLCG1, FRS2 and PAK4, leading to PI and DAG signaling, recruitment of GRB2, GAB1, PIK3R1 and SOS1, and activation of RAS, ERK1/2 and AKT1 signaling.	Predicted to be stimulatory for phosphotransferase activity.

FGR	Y412	Gardner-Rasheed feline sarcoma viral (v-fgr) oncogene	1.46	0.45	0.02	-0.25	-0.23	Non-receptor tyrosine-protein kinase that transmits signals from cell surface receptors devoid of kinase activity and contributes to the regulation of immune responses, including neutrophil, monocyte, macrophage and mast cell functions, cytoskeleton remodeling in response to extracellular stimuli, phagocytosis, cell adhesion and migration. Promotes mast cell degranulation, release of inflammatory cytokines and IgE-mediated anaphylaxis. Acts downstream of Fc-receptors, such as MS4A2/FCER1B, FCGR2A and/or FCGR2B as well as ITGB1 and ITGB2 to regulate actin cytoskeleton reorganization, cell spreading and adhesion. Negative regulator of ITGB2 signaling, phagocytosis and SYK activity in monocytes. Required for normal ITGB1 and ITGB2 signaling, normal cell spreading and adhesion in neutrophils and macrophages. Positive regulator of cell migration and regulates cytoskeleton reorganization via RAC1 activation. Phosphorylates SYK to promote AKT1 and MAPK signaling. Phosphorylates PLD2 in antigen-stimulated mast cells, leading to PLD2 activation. Promotes activation of PIK3R1. Phosphorylates FASLG, ABL1 and HCLS1. Promotes phosphorylation of CBL, CTTN, PIK3R1, PTK2/FAK1, PTK2B/PYK2 and VAV2.	Predicted to be stimulatory for phosphotransferase activity. Y412 is phosphorylated by Fgr.
FKHR	S256	Forkhead box protein O1 (FOXO1A)	-0.18	0.82	1.04	0.90	0.37	Transcription factor that is the main target of insulin signaling and regulates metabolic homeostasis in response to oxidative stress. Binds to the insulin response element (IRE). Important regulator of cell death acting downstream of CDK1, PKB/AKT1 and SKT4/MST1.	Inhibits transcription by preventing nuclear import, regulates protein degradation and molecular association; phosphorylation induces interaction with 14-3-3 beta, SKP2, and inhibits interaction with DNA. S256 is phosphorylated by Akt1 (PKBa), Akt3 (PKBg).
FOS	Pan-specific	Fos-c FBJ murine osteosarcoma oncoprotein-related transcription factor	-1.07	6.95	0.70	0.74	0.17	Nuclear phosphoprotein which forms a complex with the JUN/AP-1 transcription factor. On TGF-beta activation, forms a multimeric SMAD3/SMAD4/JUN/FOS complex at the AP1/SMAD-binding site to regulate TGF-beta signaling. Has an important role in signal transduction, cell proliferation and differentiation. Aids in regulation of cells involved in skeletal development, and, in growing cells, activates phospholipid synthesis, possibly via CDS1 and PI4K2A.	NA
FOS	T232	Fos-c FBJ murine osteosarcoma oncoprotein-related transcription factor	-0.30	0.60	0.19	1.50	0.04	See above.	Stimulates transcriptional activity. T232 is phosphorylated by ERK1, ERK2, ERK5, Frk.

FRS2	Y348	Fibroblast growth factor receptor substrate 2	-0.35	-0.14	-0.68	-0.58	-1.06	Adapter protein that links activated FGR and NGF receptors to downstream pathways. Aids in activation of MAPKs and phosphorylation of PIK3R1, the regulatory subunit of PI3K, in response to ligand-mediated activation of FGFR1. Modulates signaling via SHC1 by competing for a common binding site on NTRK1.	No data available.
GATA1	S142	Erythroid transcription factor	-0.45	-0.31	-0.26	1.50	-0.36	Transcriptional activator or repressor which probably serves as a general switch factor for erythroid development.	No data available.
GFAP	S8	Glial fibrillary acidic protein	0.95	1.03	1.35	0.90	0.76	A class-III intermediate filament and cell-specific marker; during the development of the central nervous system, it distinguishes astrocytes from other glial cells.	S8 is phosphorylated by PKACa (PRKACA), PKCa (PRKCA).
GluR1	S849	Glutamate receptor 1	0.04	1.33	0.75	0.56	0.26	Ionotropic glutamate receptor. L-glutamate acts as an excitatory neurotransmitter at many synapses in the central nervous system.	S849 is phosphorylated by the following protein kinases in vitro: CaMK2a, PKACa (PRKACA), PKCa (PRKCA).
GSK3b	Pan-specific	Glycogen synthase-serine kinase 3 beta	1.36	0.19	1.04	0.24	0.36	Constitutively active protein kinase that acts as a negative regulator in the hormonal control of glucose homeostasis, Wnt signaling and regulation of transcription factors and microtubules, by phosphorylating and inactivating glycogen synthase (GYS1 or GYS2), EIF2B, CTNNB1/beta-catenin, APC, AXIN1, DPYSL2/CRMP2, JUN, NFATC1/NFATC, MAPT/TAU and MACF1.	NA
GUK1	Y53	Guanylate kinase	-0.36	-1.24	-0.69	-0.15	-0.68	Catalyzes the phosphorylation of GMP to GDP. Essential enzyme for recycling GMP and indirectly, cyclic GMP (cGMP). May also have a role in the survival and growth progression of some tumors.	No data available.
HCA59	Y147	Uncharacterized protein C9orf78 (HSPC220)	2.39	0.25	0.67	-0.22	-0.37	Involved in the regulation of telomeric heterochromatin assembly and control of telomere length.	Y147 is phosphorylated by the following protein kinases in vitro: Syk.
HDAC4	Pan-specific	Histone deacetylase 4	0.18	0.36	1.04	0.70	0.96	Responsible for the deacetylation of lysine residues on the N-terminal part of the core histones (H2A, H2B, H3 and H4). Histone deacetylation gives a tag for epigenetic repression and plays an important role in transcriptional regulation, cell cycle progression and developmental events. Histone deacetylases act via the formation of large multiprotein complexes.	NA
HDAC4/5/9	S246	Histone deacetylase 4/5/9	-0.04	1.16	0.45	0.84	0.81	Responsible for the deacetylation of lysine residues on the N-terminal part of the core histones (H2A, H2B, H3 and H4). Histone deacetylation gives a tag for epigenetic repression and plays an important role in transcriptional regulation, cell cycle progression and developmental events. Represses MEF2-dependent transcription.	S246 is phosphorylated by the following protein kinases in vitro: CaMK1a, CaMK4, MARK2, TAK1 (MAP3K7)

HDAC5	S498	Histone deacetylase 5	0.33	1.76	0.91	0.70	0.59	Responsible for the deacetylation of lysine residues on the N-terminal part of the core histones (H2A, H2B, H3 and H4). Histone deacetylation gives a tag for epigenetic repression and plays an important role in transcriptional regulation, cell cycle progression and developmental events.	Promotes nuclear export of HDAC5 and binding to 14-3-3. S498 is phosphorylated by the following protein kinases in vitro: CaMK1a, CaMK4, PKD1 (PRKCM), PKD2, PKD3 (PRKCN).
Histone H2A.X	S139	Histone H2A variant X	2.19	0.15	2.11	0.59	0.69	Variant histone H2A which replaces conventional H2A in a subset of nucleosomes. Nucleosomes wrap and compact DNA into chromatin, limiting DNA accessibility to the cellular machineries which require DNA as a template. Histones thereby play a central role in transcription regulation, DNA repair, DNA replication and chromosomal stability.	No data available for S139, but S140 phosphorylation is critical for efficient 53BP1 foci formation for the accumulation of 53BP1 at DNA break sites. This site is phosphorylated by ATM.
Histone H2B	S14	Histone H2B	0.24	0.46	0.78	1.25	0.98	Core component of nucleosome. Nucleosomes wrap and compact DNA into chromatin, limiting DNA accessibility to the cellular machineries which require DNA as a template. Histones thereby play a central role in transcription regulation, DNA repair, DNA replication and chromosomal stability.	No data available for S14, but S15 is phosphorylated by the following protein kinases in vitro: MST1 (STK4), PKCd (PRKCD).
Histone H3	S10	Histone H3.3 (H3F3A)	1.20	-0.71	-0.39	-0.12	-0.02	Variant histone H3 which replaces conventional H3 in a wide range of nucleosomes in active genes. Constitutes the predominant form of histone H3 in non-dividing cells and is incorporated into chromatin independently of DNA synthesis. Deposited at sites of nucleosomal displacement throughout transcribed genes, suggesting that it represents an epigenetic imprint of transcriptionally active chromatin. Nucleosomes wrap and compact DNA into chromatin, limiting DNA accessibility to the cellular machineries which require DNA as a template, causing histones to play a central role in transcription regulation, DNA repair, DNA replication and chromosomal stability.	No data available.
HSP90a/b	Pan-specific	Heat shock 90 kDa protein alpha/beta	0.39	1.06	0.74	0.56	0.56	Molecular chaperone that promotes the maturation, structural maintenance and proper regulation of specific target proteins involved in cell cycle control and signal transduction. Undergoes a functional cycle that is linked to its ATPase activity which is essential for its chaperone activity. It also plays a role in the regulation of the transcription machinery. HSP90 and its co-chaperones modulate transcription at least at three different levels (alter the steady-state levels of TFs, in response to various physiological cues, modulate the activity of epigenetic modifiers, and participate in the eviction of histones from promoters). Also binds bacterial LPS and mediates LPS-induced inflammatory response, including TNF secretion by monocytes.	NA

HSP90AB1	Pan-specific	Heat shock protein HSP 90-beta (HSP90B)	0.51	1.34	0.94	0.63	0.82	See above.	NA
HSP90AB1	Y484	Heat shock protein HSP 90-beta (HSP90B)	2.10	-0.28	0.45	-0.46	0.28	See above.	No data available.
Huntingtin	S421	Huntington's disease protein	2.56	-0.21	1.08	0.42	0.75	May play a role in microtubule-mediated transport or vesicle function.	No data available.
IGF1R	Y1165/Y1166	Insulin-like growth factor 1 receptor protein-tyrosine kinase	-0.58	-0.42	0.25	1.38	0.50	Receptor tyrosine kinase which mediates the pleiotropic actions of insulin. Binding of insulin leads to phosphorylation of several intracellular substrates, including, insulin receptor substrates (IRS1, 2, 3, 4), SHC, GAB1, CBL and other signaling intermediates. Each of these phosphorylated proteins serve as docking proteins for other signaling proteins that contain Src-homology-2 domains (SH2 domain) that specifically recognize different phosphotyrosine residues, including the p85 regulatory subunit of PI3K and SHP2. Phosphorylation of IRSs proteins lead to the activation of two main signaling pathways: the PI3K-AKT/PKB pathway and the Ras-MAPK pathway. The result of activating the MAPK pathway is increased cellular proliferation, whereas activating the PI3K pathway inhibits apoptosis and stimulates protein synthesis.	(1165) Stimulates phosphotransferase activity. Phosphorylation regulates conformation. Phosphorylated by Src. (1166) Stimulates phosphotransferase activity. Phosphorylation regulates cell growth and protein conformation. Y1166 is phosphorylated by IGF1R, Src
IGF1R	Y1280	Insulin-like growth factor 1 receptor protein-tyrosine kinase	1.89	1.46	2.06	2.41	1.73	See above.	Phosphorylation regulates cell differentiation and cell growth. Y1280 is phosphorylated by the following protein kinases in vitro: IGF1R.
IkBb	Pan-specific	Inhibitor of NF-kappa-B beta (thyroid receptor interacting protein 9)	-0.52	1.78	-0.22	0.69	-0.32	Inhibits the activity of dimeric NF-kappa-B/REL complexes by trapping REL dimers in the cytoplasm through masking of their nuclear localization signals. On cellular stimulation by immune and proinflammatory responses, becomes phosphorylated promoting ubiquitination and degradation, enabling the dimeric RELA to translocate to the nucleus and activate transcription.	NA
IkBe	S161	NF-kappa-B inhibitor epsilon	0.33	0.95	1.50	1.76	1.40	Inhibits NF-kappa-B by complexing with and trapping it in the cytoplasm. Inhibits DNA-binding of NF-kappa-B p50-p65 and p50-c-Rel complexes.	No data available.
IKKa	Pan-specific	Inhibitor of NF-kappa-B protein-serine kinase alpha (CHUK, IkBKA)	2.10	1.27	1.81	1.61	2.29	Serine kinase that plays an essential role in the NF-kappa-B signaling pathway which is activated by multiple stimuli such as inflammatory cytokines, bacterial or viral products, DNA damages or other cellular stresses. Acts as part of the canonical IKK complex in the conventional pathway of NF-kappa-B activation and phosphorylates inhibitors of NF-kappa-B on serine residues.	NA

ILK1	Pan-specific	Integrin-linked protein-serine kinase 1	0.01	-1.44	-0.83	-1.27	-0.99	Receptor-proximal protein kinase regulating integrin-mediated signal transduction. May act as a mediator of inside-out integrin signaling. Focal adhesion protein part of the complex ILK-PINCH. This complex is considered to be one of the convergence points of integrin-and growth factor-signaling pathway. Could be implicated in mediating cell architecture, adhesion to integrin substrates and anchorage-dependent growth in epithelial cells. Phosphorylates beta-1 and beta-3 integrin subunit on serine and threonine residues, but also AKT1 and GSK3B.	NA
InsR (IR)	Y1189	Insulin receptor beta chain	0.00	-0.98	-0.23	-0.67	-0.05	Receptor tyrosine kinase which mediates the pleiotropic actions of insulin. Binding of insulin leads to phosphorylation of several intracellular substrates, including, insulin receptor substrates (IRS1, 2, 3, 4), SHC, GAB1, CBL and other signaling intermediates. Each of these phosphorylated proteins serve as docking proteins for other signaling proteins that contain Src-homology-2 domains (SH2 domain) that specifically recognize different phosphotyrosine residues, including the p85 regulatory subunit of PI3K and SHP2. Phosphorylation of IRSs proteins lead to the activation of the PI3K-AKT/PKB pathway, which is responsible for most of the metabolic actions of insulin, and the Ras-MAPK pathway, which regulates expression of some genes and cooperates with the PI3K pathway to control cell growth and differentiation.	Stimulates phosphotransferase activity, receptor internalization and induces interaction with SOCS1, IRS2 and PTP1B. Y1189 is phosphorylated by InsR.
InsR (IR)	Pan-specific	Insulin receptor beta chain	2.18	1.80	5.52	3.33	2.34	See above.	NA
InsR (IR)	Y999	Insulin receptor beta chain	0.54	1.43	1.45	2.13	1.78	See above.	Stimulates phosphotransferase activity and induces interaction with IRS1 and Shc1 (via their PTB/PID domains), STAT5B and SOCS3. Phosphorylation regulates cell cycle progression, receptor internalization, and molecular association. Y999 is phosphorylated by InsR.
Integrin a4	S1021	Integrin alpha 4 (VLA4, ITGA4)	0.17	1.18	1.19	1.83	1.14	Integrins alpha-4/beta-1 (VLA-4) and alpha-4/beta-7 are receptors for fibronectin. They recognize one or more domains within the alternatively spliced CS-1 and CS-5 regions of fibronectin. They are also receptors for VCAM1. Integrin alpha-4/beta-1 recognizes the sequence Q-I-D-S in VCAM1. Integrin alpha-4/beta-7 is a receptor for MADCAM1.	No data available.

IRS1	S312	Insulin receptor substrate 1	0.71	0.19	0.81	1.10	1.27	May mediate the control of various cellular processes by insulin. When phosphorylated by the insulin receptor binds specifically to various cellular proteins containing SH2 domains such as PI3K p85 subunit or GRB2.	Regulates apoptosis, alters intracellular location, protein degradation, regulates molecular association, and inhibits interaction with IKK-alpha, IKK-beta. S312 is phosphorylated by CK2a1 (CSNK2A1), ERK1, ERK2, IKKa, IKKb, JNK1, p70S6K, PKCa (PRKCA), PKCz (PRKCZ).
IRS1	S639	Insulin receptor substrate 1	-0.33	0.93	0.48	1.43	0.29	See above.	Regulates intracellular location and Inhibition of tyrosine phosphorylation. S639 is phosphorylated by BARK1 (GRK2; ADRBK1), ERK2, FRAP1 (mTOR), p70S6K (RPS6KB1), ROCK1, ROCK2.
ITSN2	Y968	Intersectin-2	2.43	0.43	1.36	1.13	1.81	Adapter protein that may provide indirect link between the endocytic membrane traffic and the actin assembly machinery. May regulate the formation and maturation of clathrin-coated vesicles (CCPs). Involved in endocytosis of integrin beta-1 (ITGB1) and transferrin receptor (TFR).	No data available.
JAK1	Y1034	Janus protein-tyrosine kinase 1	0.26	2.18	3.44	3.86	2.98	Tyrosine kinase of the non-receptor type, involved in the IFN-alpha/beta/gamma signal pathway. Kinase partner for the interleukin (IL)-2 and interleukin (IL)-10 receptors.	Stimulates phosphotransferase activity.
JAK1	Pan-specific	Janus protein-tyrosine kinase 1	0.33	-0.05	0.68	0.57	1.02	See above.	NA
JNK2	Pan-specific	Jun N-terminus protein-serine kinase (stress-activated protein kinase (SAPK)) 2 (SAPKa, MAPK9)	2.36	4.51	2.96	4.15	4.39	Serine/threonine-protein kinase involved in cell proliferation, differentiation, migration, transformation and programmed cell death. Extracellular stimuli such as proinflammatory cytokines or physical stress stimulate the stress-activated protein kinase/c-Jun N-terminal kinase (SAP/JNK) signaling pathway. Activated by MAP2K4/MKK4 and MAP2K7/MKK7. Phosphorylates a number of transcription factors, primarily components of AP-1 such as JUN, JDP2 and ATF2.	NA
JNK3	Pan-specific	Jun N-terminus protein-serine kinase (stress-activated protein kinase (SAPKb)) 3 (SAPKb, MAPK10)	1.24	0.12	0.78	0.33	0.83	Serine/threonine-protein kinase involved in cell proliferation, differentiation, migration, transformation and programmed cell death. Extracellular stimuli such as proinflammatory cytokines or physical stress stimulate the SAP/JNK signaling pathway. Activated by MAP2K4/MKK4 and MAP2K7/MKK7. Phosphorylates a number of transcription factors, primarily components of AP-1 such as JUN, JDP2 and ATF2.	NA

JUN	Pan-specific	Jun proto-oncogene-encoded AP1 transcription factor (c-Jun)	1.32	0.68	2.83	0.78	1.20	Transcription factor that recognizes and binds to the enhancer heptamer motif 5'-TGA[CG]TCA-3'. Promotes activity of NR5A1 when phosphorylated by HIPK3 leading to increased steroidogenic gene expression upon cAMP signaling pathway stimulation. Also involved in K-RAS signaling response.	NA
JUN	S243	Jun proto-oncogene-encoded AP1 transcription factor (c-Jun)	-0.26	1.44	0.35	1.58	-0.25	See above.	Inhibits transcriptional activity - reducing binding of DNA. S243 is phosphorylated by CDK1 (CDC2), CK2a1 (CSNK2A1), DYRK2, ERK1 (MAPK3), ERK2 (MAPK1), GSK3b.
JUN	Y170	Jun proto-oncogene-encoded AP1 transcription factor (c-Jun)	-0.33	1.40	0.29	1.78	0.44	See above.	Y170 is phosphorylated by Abl1.
KHS1	S174	Kinase homologous to SPS1/STE20 (MAP kinase kinase kinase protein-serine kinase 5 (MEKKK5)	1.18	-0.34	0.67	0.04	0.88	May play a role in the response to environmental stress. Appears to act upstream of the JUN N-terminal pathway.	Stimulates phosphotransferase activity. This phosphosite is located in the kinase activation loop between catalytic subdomains VII and VIII.
KIT	Y721	'Mast/stem cell growth factor receptor Kit	0.21	2.30	1.73	2.02	1.50	Tyrosine-protein kinase that acts as cell-surface receptor for the cytokine KITLG/SCF, leading to regulation of cell survival and proliferation, hematopoiesis, stem cell maintenance, gametogenesis, mast cell development, migration and function, and in melanogenesis. Phosphorylates PIK3R1, PLCG1, SH2B2/APS and CBL. Activates the AKT1 signaling pathway by phosphorylation of PIK3R1, the regulatory subunit of PI3K. Activated KIT also signals via GRB2 and activation of RAS, RAF1 and ERK1/2 MAPKs. Promotes activation of STAT family members STAT1, STAT3, STAT5A and STAT5B.	Stimulates phosphotransferase activity and induces interaction with PIK3R1. Phosphorylation regulates cell adhesion. Y721 is phosphorylated by the following protein kinases in vitro: Kit.
KIT	Y936	'Mast/stem cell growth factor receptor Kit	4.59	2.71	3.14	3.41	2.76	See above.	Stimulates phosphotransferase activity and induces interaction with Grb2 and Grb7. Phosphorylation also induces receptor internalization and protein degradation. Y936 is phosphorylated by Kit.

KSR	Pan-specific	Protein-serine kinase suppressor of Ras 1	1.52	2.08	2.53	3.10	3.90	Scaffolding protein that is part of a multiprotein signaling complex. Promotes phosphorylation of Raf family members and activation of downstream MAPKs. Promotes activation of MAPK1 and/or MAPK3 in response to EGF and to cAMP. Independently of its kinase activity, acts as MAP2K1/MEK1 and MAP2K2/MEK2-dependent allosteric activator of BRAF; upon binding to MAP2K1/MEK1 or MAP2K2/MEK2, dimerizes with BRAF and promotes BRAF-mediated phosphorylation of MAP2K1/MEK1 and/or MAP2K2/MEK2.	NA
LCK	Y394	Lymphocyte-specific protein-tyrosine kinase	0.01	1.41	0.78	1.44	0.16	Non-receptor tyrosine kinase that plays an essential role in the selection and maturation of developing T-cells in the thymus and in the function of mature T-cells. Plays a key role in T-cell antigen receptor (TCR)-linked signal transduction pathways.	Stimulates phosphotransferase activity. Phosphorylation regulates cell growth and molecular association. Y394 is phosphorylated by Lck.
LIMK1	Pan-specific	LIM domain kinase 1	1.44	-0.21	1.01	-0.16	0.42	Serine/threonine kinase that regulates actin filament dynamics. Acts downstream of several Rho family GTPase signal transduction pathways. Activated by upstream kinases including ROCK1, PAK1 and PAK4. LIMK1 subsequently phosphorylates and inactivates the actin binding/depolymerizing factors cofilin-1/CFL1, cofilin-2/CFL2 and destrin/DSTN, thereby preventing the cleavage of F-actin, and stabilizing the actin cytoskeleton.	NA
MAPKAPK3	Y76	MAP kinase-activated protein kinase 3	1.92	0.06	1.17	0.47	0.67	Stress-activated serine/threonine-protein kinase involved in cytokine production, endocytosis, reorganization of the cytoskeleton, cell migration, cell cycle control, chromatin remodeling, DNA damage response and transcriptional regulation. Following stress, it is phosphorylated and activated by MAPK p38-alpha/MAPK14, leading to phosphorylation of substrates.	No data available.
MEK1/2	S218+S222	MAPK/ERK protein-serine kinase 1/2 (MKK1/2, MAP2K1/2)	0.24	0.71	1.34	-0.37	-0.33	Dual specificity kinase which acts as an essential component of the MAPK signal transduction pathway. Binding of extracellular ligands such as growth factors, cytokines and hormones to their cell-surface receptors activates RAS and initiates RAF1 activation. RAF1 activates the MAP2K1/MEK1 and MAP2K2/MEK2. Both MAP2K1/MEK1 and MAP2K2/MEK2 phosphorylate and activate MAPK3/ERK1 and MAPK1/ERK2. Depending on the cellular context, this pathway mediates diverse biological functions such as cell growth, adhesion, survival and differentiation, predominantly through the regulation of transcription, metabolism and cytoskeletal rearrangements.	Stimulates phosphotransferase activity and regulates cell cycle progression and cell growth. S218 is phosphorylated by B-Raf, COT (MAP3K8), MEKK1 (MAP3K1), Mos, Raf1, RafA. S222 is phosphorylated by B-Raf, COT (MAP3K8), MEKK1 (MAP3K1), Mos, PDK1 (PDPK1), Raf1, RafA.

MEK3/6	S218/S207	MAPK/ERK protein-serine kinase 3 beta isoform (MKK3 beta, MAP2K3)	1.34	2.73	2.07	0.92	1.08	Dual specificity kinase. Is activated by cytokines and environmental stress in vivo. Phosphorylates and activates the MAPK p38. Part of a signaling cascade that begins with the activation of the adrenergic receptor ADRA1B and leads to the activation of MAPK14.	Stimulates phosphotransferase activity and regulates transcription. S218 is phosphorylated by ASK1 (MAP3K5), COT (MAP3K8), MEKK3 (MAP3K3), MKK3 (MAP2K3, MEK3), MLK3 (MAP3K11).
MEKK1	Pan-specific	MAPK/ERK kinase kinase 1 (MAP3K1)	0.42	0.73	1.04	0.85	1.06	Upstream member of the MAPK family that activates the ERK and JNK kinase pathways by phosphorylation of MAP2K1 and MAP2K4. Activates CHUK and IKBKB, the central protein kinases of the NF-kappa-B pathway.	NA
MET	Pan-specific	Hepatocyte growth factor (HGF) receptor-tyrosine kinase	0.15	0.33	1.00	0.86	0.98	Receptor tyrosine kinase that transduces signals from the ECM into the cytoplasm by binding to hepatocyte growth factor/HGF ligand. Regulates many physiological processes including proliferation, scattering, morphogenesis and survival. Ligand binding induces autophosphorylation of MET, providing docking sites for downstream signaling molecules. Following activation, interacts with the PI3K subunit PIK3R1, PLCG1, SRC, GRB2, STAT3 or the adapter GAB1. Recruitment of these effectors leads to activation of RAS-ERK, PI3K-AKT, and PLCgamma-PKC signaling.	NA
MKK7	T275	MAPK/ERK protein-serine kinase 7 (MEK7, MAP2K7)	1.10	0.40	1.41	1.22	0.74	Dual specificity protein kinase which is an essential component of the stress-activated protein kinase/c-Jun N-terminal kinase (SAP/JNK) MAPK signaling pathway. With MAP2K4/MKK4, directly activates MAPK8/JNK1, MAPK9/JNK2 and MAPK10/JNK3. MAP2K4/MKK4 and MAP2K7/MKK7 both activate the JNKs by phosphorylation, but they differ in their preference for the phosphorylation site in the Thr-Pro-Tyr motif. MAP2K4/MKK4 shows preference for phosphorylation of the Tyr residue and MAP2K7/MKK7 for the Thr residue. The monophosphorylation of JNKs on the Thr residue is sufficient to increase JNK activity indicating that MAP2K7/MKK7 is important to trigger JNK activity, while the additional phosphorylation of the Tyr residue by MAP2K4/MKK4 ensures optimal JNK activation. Has a specific role in JNK signal transduction pathway activated by proinflammatory cytokines. The MKK/JNK signaling pathway is also involved in mitochondrial death signaling pathway, including the release cytochrome c, leading to apoptosis.	Stimulates phosphotransferase activity. This phosphosite is located in the kinase activation loop between catalytic subdomains VII and VIII. T275 is phosphorylated by the following protein kinases in vitro: ASK1 (MAP3K5), DLK (MAP3K12), MEKK1 (MAP3K1), MEKK2 (MAP3K2), MEKK3 (MAP3K3), MLK3 (MAP3K11).

MSK1	S376	Mitogen & stress-activated protein-serine kinase 1 (RPS6KA5)	0.93	1.17	2.52	1.76	1.05	Serine/threonine kinase that is required for mitogen, cytokine, or other stress-induced phosphorylation of CREB1 and ATF1 and for the regulation of RELA, STAT3 and ETV1/ER81 in response to these stressors. This contributes to gene activation by histone phosphorylation and functions in the regulation of inflammatory genes.	Stimulates phosphotransferase activity. S376 is phosphorylated by MSK1 (RPS6KA5).
MST1	Pan-specific	Mammalian STE20-like protein-serine kinase 1 (KRS2, STK4)	0.02	0.99	2.97	-0.63	-1.05	Stress-activated, pro-apoptotic kinase which, following caspase-cleavage, enters the nucleus and induces chromatin condensation followed by internucleosomal DNA fragmentation. Key component of the Hippo signaling pathway which plays a pivotal role in organ size control and tumor suppression by restricting proliferation and promoting apoptosis. STK3/MST2 and STK4/MST1, in complex with SAV1, phosphorylate and activate LATS1/2 in complex with its regulatory protein MOB1. This leads to phosphorylation and inactivation of YAP1 and WWTR1/TAZ.	NA
mTOR	S2478+S2481	Mammalian target of rapamycin (FRAP)	0.31	0.87	1.37	1.79	0.63	Serine/threonine protein kinase which is a central regulator of cellular metabolism, growth and survival in response to hormones, growth factors, nutrients, energy and stress signals. MTOR directly or indirectly regulates the phosphorylation of at least 800 proteins. Functions as part of 2 structurally and functionally distinct signaling complexes mTORC1 and mTORC2 (mTOR complex 1 and 2). Activated mTORC1 up-regulates protein synthesis by phosphorylating key regulators of mRNA translation and ribosome synthesis. This includes phosphorylation of EIF4EBP1 and release of its inhibition toward the elongation initiation factor 4E (eiF4E). Moreover, phosphorylates and activates RPS6KB1 and RPS6KB2 that promote protein synthesis by modulating the activity of their downstream targets including ribosomal protein S6, eukaryotic translation initiation factor EIF4B, and the inhibitor of translation initiation PDCD4.	S2478 is phosphorylated by the following protein kinases in vitro: FRAP1 (mTOR
mTOR	Pan-specific	Mammalian target of rapamycin (FRAP)	0.57	1.11	2.16	0.81	1.14	See above.	NA
MYC	S373	Myc proto-oncogene protein	0.87	1.19	1.70	0.39	0.32	Transcription factor that binds DNA in a non-specific manner, yet also specifically recognizes the core sequence 5'-CAC[GA]TG-3'. Activates the transcription of growth-related genes. Binds VEGFA promoter, promoting VEGFA production and angiogenesis.	S373 is phosphorylated by the following protein kinases in vitro: PAK2
MYC	T58	Myc proto-oncogene protein	0.46	0.93	1.07	0.04	0.56	See above.	T58 is phosphorylated by the following protein kinases in vitro: ERK2 (MAPK1), GSK3a, GSK3b, JNK3 (MAPK10)

NBS1	S343	Nijmegen breakage syndrome protein 1 (NBN, Nibrin)	0.50	0.91	1.40	0.20	0.31	Component of the MRN complex which plays a critical role in the cellular response to DNA damage and the maintenance of chromosome integrity. The complex is involved in double-strand break repair, DNA recombination, maintenance of telomere integrity, cell cycle checkpoint control and meiosis.	S343 is phosphorylated by ATM.
NEK2	T170+S171	NIMA (never-in-mitosis)-related protein-serine kinase 2	0.22	1.96	2.09	3.39	2.02	Protein kinase which is involved in the control of centrosome separation and bipolar spindle formation in mitotic cells and chromatin condensation in meiotic cells. Regulates centrosome separation by phosphorylating CROCC, CEP250 and NINL. Regulates kinetochore microtubule attachment stability via phosphorylation of NDC80. Involved in regulation of mitotic checkpoint protein complex via phosphorylation of CDC20 and MAD2L1. Regulates chromatin condensation through phosphorylation of HMGA2.	Stimulates phosphotransferase activity.
NEK7	Pan-specific	NIMA (never-in-mitosis)-related protein-serine kinase 7	1.15	0.12	-0.15	0.37	0.06	Protein kinase which plays an important role in mitotic cell cycle progression. Required for microtubule nucleation activity of the centrosome, robust mitotic spindle formation and cytokinesis. Phosphorylates RPS6KB1.	NA
NFkappaB p65	S536	NF-kappa-B p65 nuclear transcription factor (Rel A)	0.01	1.27	0.96	0.94	0.42	Pleiotropic transcription factor present in almost all cell types and is the endpoint of signal transduction events initiated by a vast array of stimuli related to many biological processes such as inflammation, immunity, differentiation, cell growth, tumorigenesis and apoptosis. NF-kappa-B is a homo- or heterodimeric complex formed by the Rel-like domain-containing proteins RELA/p65, RELB, NFKB1/p105, NFKB1/p50, REL and NFKB2/p52 and the heterodimeric p65-p50 complex appears to be most abundant one.	S536 is phosphorylated by the following protein kinases in vitro: CaMK4, IKKa (CHUK), IKKb (IKBKINASE), IKKe (IKBKE), TBK1
NR1 (NMDAR1)	S896	N-methyl-D-aspartate (NMDA) glutamate receptor 1 subunit zeta	-0.37	0.63	1.63	0.34	-0.07	NMDA receptor subtype of glutamate-gated ion channels with high Ca permeability and voltage-dependent sensitivity to Mg. Mediated by glycine. Key role in synaptic plasticity, synaptogenesis, excitotoxicity, memory acquisition and learning. It mediates neuronal functions in glutamate neurotransmission.	S896 is phosphorylated by the following protein kinases in vitro: PKCa (PRKCA).
p38a MAPK	T180+Y182	Mitogen-activated protein-serine kinase p38 alpha (MAPK14)	3.92	-0.36	0.45	0.02	0.24	MAPK pathway serine/threonine kinase. One of the four p38 MAPKs which play an important role in the cascades of cellular responses evoked by extracellular stimuli such as proinflammatory cytokines or physical stress leading to direct activation of transcription factors (TFs). Phosphorylates a broad range of proteins (200- 300 substrates). Many targets are kinases which phosphorylate additional downstream targets. RPS6KA5/ MSK1 and RPS6KA4/MSK2 directly phos-phorylate/activate TFs CREB1, ATF1, the NF-kappa-B isoform RELA/NFKB3, STAT1 and STAT3, but can also phos-phorylate histone H3 and nucleosomal protein HMGN1.	Stimulates phospho-transferase activity. Phosphorylation regulates apoptosis, cell adhesion, cell cycle progression, cell motility, cytoskeletal reorganization, and molecular association, including inducing interaction w/ Fyn, p38a, Nck1. T180 phosphoryl-ated by MKK3 (MAP2K3, MEK3), MKK4 (MAP2K4, MEK4), MKK6 (MAP2K6, MEK6), p38a, PBK/TOPK

p38a MAPK	Pan-specific	Mitogen-activated protein-serine kinase p38 alpha (MAPK14)	3.77	2.34	4.26	3.71	4.15	See above.	NA
p38a MAPK	Pan-specific	Mitogen-activated protein-serine kinase p38 alpha (MAPK14)	3.93	1.78	3.42	3.28	3.39	See above.	NA
p38b MAPK	T180+Y182	Mitogen-activated protein-serine kinase p38 beta (MAPK11)	1.22	0.27	0.05	1.80	0.44	Serine/threonine kinase which acts as an essential component of the MAP kinase signal transduction pathway. MAPK11 is one of the four p38 MAPKs which play an important role in the cascades of cellular responses evoked by extracellular stimuli such as proinflammatory cytokines or physical stress leading to direct activation of transcription factors. Accordingly, p38 MAPKs phosphorylate a broad range of proteins and it has been estimated that they may have approximately 200 to 300 substrates each. MAPK11 functions are mostly redundant with those of MAPK14.	Stimulates phospho-transferase activity. Phosphorylation regulates apoptosis, cell adhesion, cell cycle progression, cell motility, cytoskeletal reorganization, and molecular association, including inducing interaction with Fyn, Nck1, p38a. T180 is phosphorylated by MKK3 (MAP2K3, MEK3), MKK4 (MAP2K4, MEK4), MKK6 (MAP2K6, MEK6), p38a MAPK (MAPK14), PBK (TOPK).
p38b MAPK	Pan-specific	Mitogen-activated protein-serine kinase p38 beta (MAPK11)	2.93	-0.46	1.19	1.59	0.32	See above.	NA
p38b MAPK	Pan-specific	Mitogen-activated protein-serine kinase p38 beta (MAPK11)	3.37	0.57	0.14	1.83	0.94	See above.	NA
p38d MAPK	Pan-specific	Mitogen-activated protein-serine kinase p38 delta (MAPK13)	1.58	1.05	1.51	0.62	1.10	Serine/threonine kinase which acts as an essential component of the MAP kinase signal transduction pathway. MAPK13 is one of the four p38 MAPKs which play an important role in the cascades of cellular responses evoked by extracellular stimuli such as proinflammatory cytokines or physical stress leading to direct activation of transcription factors such as ELK1 and ATF2. MAPK13 is one of the less studied p38 MAPK isoforms. Some of its targets are: MAPKAPK2, EEF2K, MAPT, STMN1, and CXCL14.	NA
p38g MAPK	Pan-specific	Mitogen-activated protein-serine kinase p38 gamma, ((MAPK12, ERK6)	0.58	0.35	2.08	0.88	0.89	See above.	NA
p38g MAPK	Pan-specific	Mitogen-activated protein-serine kinase p38 gamma, ((MAPK12, ERK6)	1.29	0.29	1.13	1.52	0.71	See above.	NA

p53	Pan-specific	Tumor suppressor protein p53 (antigenNY-CO-13) (TP53)	-0.20	-0.42	-0.94	-0.65	-1.11	Induces growth arrest or apoptosis depending on the physiological circumstances and cell type. Involved in cell cycle regulation as a trans-activator that acts to negatively regulate cell division by controlling genes required for this process. One of the activated genes is an inhibitor of cyclin-dependent kinases. Apoptosis induction is mediated by stimulation of BAX and FAS antigen expression, or by repression of Bcl-2 expression. In cooperation with mitochondrial PPIF, is involved in activating oxidative stress-induced necrosis (largely independent of transcription).	NA
p53	S33	Tumor suppressor protein p53 (antigenNY-CO-13) (TP53)	0.41	0.45	0.97	1.03	1.02	See above.	Activation; Binding to CBP, Pin1: Inhibits binding to MDM2. S33 is phosphorylated by the following protein kinases in vitro: BARK2 (GRK3, ADRBK2), CDK5, CDK7, CDK9, GSK3b, p38a MAPK (MAPK14).
p53	S37	Tumor suppressor protein p53 (antigenNY-CO-13) (TP53)	0.63	1.33	1.14	2.92	1.46	See above.	Activation; Binding to CBP, PPP2CA: Inhibits binding to MDM2. S37 is phosphorylated by the following protein kinases in vitro: ATM, ATR, Chk1 (CHEK1), Chk2 (CHEK2), DNAPK (PRKDC), JNK1 (MAPK8), JNK3 (MAPK10), MAPKAPK5.
p53	S6	Tumor suppressor protein p53 (antigenNY-CO-13) (TP53)	0.54	0.65	2.00	1.61	2.34	See above.	Activation of transcriptional activity. S6 is phosphorylated by ATM, CK1d1 (CSNK1D), CK1e1 (CSNK1E).
p70 S6K	Pan-specific	Ribosomal protein S6 kinase beta-1 (RPS6KB1 p70S6Ka)	0.25	-0.60	-0.51	0.15	1.06	Serine/threonine kinase that acts downstream of mTOR signaling in response to growth factors and nutrients to promote cell proliferation, cell growth and cell cycle progression. Regulates protein synthesis through phosphorylation of EIF4B, RPS6 and EEF2K, and contributes to cell survival by repressing the pro-apoptotic function of BAD. Under conditions of nutrient depletion, the inactive form associates with the EIF3 translation initiation complex to inhibit activity.	NA
p70 S6K	S434	Ribosomal protein S6 kinase beta-1 (RPS6KB1, p70S6Ka)	-0.02	1.29	1.57	0.56	2.08	See above.	Stimulates phosphotransferase activity. S434 is phosphorylated by CDK1 (CDC2), CDK5, ERK1 (MAPK3), ERK2 (MAPK1), FRAP1 (mTOR), JNK1 (MAPK8), JNK2 (MAPK9).

p70 S6K	S447	Ribosomal protein S6 kinase beta-1 (RPS6KB1, p70S6Ka)	0.40	2.12	1.75	2.87	2.68	See above.	Possible activation site.
p70 S6K	T252	Ribosomal protein S6 kinase beta-1 (RPS6KB1, p70S6Ka)	1.56	3.26	3.73	3.70	0.77	See above.	Stimulates phosphotransferase activity. Phosphorylation regulates protein translation and protein conformation. T252 is phosphorylated by PDK1 (PDPK1), PIK3CD.
PAK1	Pan-specific	p21-activated kinase 1 (alpha) (serine/threonine-protein kinase PAK 1) (PAKa)	1.44	0.57	0.01	2.46	0.09	Protein kinase involved in intracellular signaling downstream of integrins and receptor-type kinases that plays an important role in actin and microtubule cytoskeleton dynamics (including actin stress fibers, focal adhesion complexes, and microtubule biogenesis and organization), in cell adhesion, migration, proliferation, apoptosis, mitosis, and in vesicle-mediated transport processes. Phosphorylates BAD to protect cells against apoptosis. Activated by interaction with CDC42 and RAC1. Functions as GTPase effector that links the Rho-related GTPases CDC42 and RAC1 to the JNK MAP kinase pathway. Phosphorylates and activates MAP2K1, and thereby mediates activation of downstream MAP kinases. Plays a role in the regulation of insulin secretion in response to elevated glucose levels.	NA
PAK1	S144	p21-activated kinase 1 (alpha) (serine/threonine-protein kinase PAK 1) (PAKa)	2.26	0.17	0.26	1.40	0.50	See above.	Stimulates phosphotransferase activity and regulates cytoskeletal reorganization, and intracellular location. S144 is phosphorylated by PAK1.
PAK1	T212	p21-activated kinase 1 (alpha) (serine/threonine-protein kinase PAK 1) (PAKa)	0.78	1.82	0.46	0.60	0.15	See above.	T212 is targeted by the neuronal p35/Cdk5 kinase; results suggest that this site regulates the microtubule cytoskeleton. Cyclin B1/Cdc2 phosphorylates Pak1 in cells undergoing mitosis.
PAK1	Pan-specific	p21-activated kinase 1 (alpha) (serine/threonine-protein kinase PAK 1) (PAKa)	1.76	-0.23	0.13	0.50	0.62	See above.	NA

PAK2	Pan-specific	p21-activated kinase 2 (gamma) (serine/threonine-protein kinase PAK 2) (PAKg)	2.35	0.71	-0.63	1.16	-0.30	Serine/threonine protein kinase that plays a role in cytoskeleton regulation, cell motility, cell cycle progression, apoptosis or proliferation. Acts as downstream effector of the small GTPases CDC42 and RAC1. Full-length PAK2 stimulates cell survival and cell growth. Phosphorylates MAPK4 and MAPK6 and activates the downstream target MAPKAPK5, a regulator of F-actin polymerization and cell migration. Phosphorylates JUN and plays a role in EGF-induced cell proliferation. Phosphorylates many other substrates including histone H4 to promote assembly of H3.3 and H4 into nucleosomes, BAD, ribosomal protein S6, or MBP. Apoptotic stimuli such as DNA damage lead to caspase-mediated cleavage of PAK2, generating an active p34 fragment that translocates to the nucleus and promotes apoptosis via the JNK pathway. Caspase-activated PAK2 phosphorylates MKNK1 and reduces cellular translation.	NA
PAK2	S141	p21-activated kinase 2 (gamma) (serine/threonine-protein kinase PAK 2) (PAKg)	1.53	0.77	0.57	1.89	1.63	See above.	Stimulates phosphotransferase activity. S141 is phosphorylated by the following protein kinases in vitro: PAK2.
PAK2	Y130	p21-activated kinase 2 (gamma) (serine/threonine-protein kinase PAK 2) (PAKg)	2.82	1.34	-0.23	0.43	0.50	See above.	Stimulates phosphotransferase activity. Y130 is phosphorylated by the following protein kinases in vitro: Src.
PAK2	Pan-specific	p21-activated kinase 2 (gamma) (serine/threonine-protein kinase PAK 2) (PAKg)	2.13	1.33	0.84	1.11	0.71	See above.	NA
PAK5	S602	p21-activated kinase 5 (serine/threonine-protein kinase PAK 7)	0.93	-0.74	-0.86	-0.26	-1.07	Serine/threonine protein kinase that plays a role in a variety of different signaling pathways including cytoskeleton regulation, cell migration, proliferation or cell survival. Activation by various effectors including growth factor receptors or active CDC42 and RAC1 results in a conformational change and a subsequent autophosphorylation on several serine and/or threonine residues. Phosphorylates the proto-oncogene RAF1 and stimulates its kinase activity. Promotes cell survival by phosphorylating the BCL2 antagonist of cell death BAD. Phosphorylates CTNND1, probably to regulate cytoskeletal organization and cell morphology. Keeps microtubules stable through MARK2 inhibition and destabilizes the F-actin network leading to the disappearance of stress fibers and focal adhesions.	Predicted to be stimulatory for phosphotransferase activity.

Paxillin 1	Y118	Paxillin 1 (PXN)	0.02	1.48	1.19	1.43	1.04	Cytoskeletal protein involved in actin-membrane attachment at sites of cell adhesion to the extracellular matrix (focal adhesion).	Activation and binding to CrkL, Src. Y118 is phosphorylated by Abl1, Brk (PTK6), FAK (PTK2), Fyn, PYK2 (PTK2B), Src.
PBK	Y74	Lymphokine-activated killer T-cell-originated protein kinase	-0.59	-0.63	-1.07	-0.53	-0.99	Phosphorylates MAP kinase p38. Seems to be active only in mitosis. May also play a role in the activation of lymphoid cells. When phosphorylated, forms a complex with TP53, leading to TP53 destabilization and attenuation of G2/M checkpoint during doxorubicin-induced DNA damage.	No data available.
PCTAIRE2	S180	Cell division protein kinase 17 (CDK17, PCTK2)	1.11	-0.08	-0.63	0.23	-0.21	May play a role in terminally differentiated neurons. Has a Ser/Thr-phosphorylating activity for histone H1.	No data available; some of other sites phosphorylated by PKACA.
PDGFRa	Pan-specific	Platelet-derived growth factor receptor kinase alpha	0.95	0.21	0.66	0.60	1.49	Tyrosine kinase that acts as a cell-surface receptor for PDGFA, PDGFB and PDGFC and plays an essential role in the regulation of embryonic development, cell proliferation, survival and chemotaxis. Depending on the context, promotes or inhibits cell proliferation and cell migration. Plays an important role in the differentiation of bone marrow-derived mesenchymal stem cells. Required for normal skeleton development and cephalic closure during embryonic development. Required for normal development of the mucosa lining the gastrointestinal tract, and for recruitment of mesenchymal cells and normal development of intestinal villi. Plays a role in cell migration and chemotaxis in wound healing. Plays a role in platelet activation, secretion of agonists from platelet granules, and in thrombin-induced platelet aggregation.	NA
PKCb	Pan-specific	Protein-serine kinase C beta 1 (PRKCB1)	0.36	1.34	0.60	2.07	1.26	Calcium-activated, phospholipid- and diacylglycerol (DAG)-dependent serine/threonine kinase involved in various cellular processes such as regulation of the B-cell receptor (BCR) signalosome, oxidative stress-induced apoptosis, androgen receptor-dependent transcription regulation, insulin signaling and endothelial cell proliferation. Plays a key role in B-cell activation by regulating BCR-induced NF-kappa-B activation. Mediates the activation of the canonical NF-kappa-B pathway.	NA
PKCb2	T642	Protein-serine kinase C beta 2 (PRKCB2)	-0.02	1.11	1.70	0.59	1.31	See above.	Stimulates phosphotransferase activity and regulates intracellular location. T642 is phosphorylated by the following protein kinases in vitro: PKCb1 (PRKCB1).

PKCd	S645	Protein-serine kinase C delta (PRKCD)	0.79	1.20	0.87	1.76	1.24	Ca-independent, phospholipid- and DAG-dependent serine/threonine kinase that plays contrasting roles in cell death and cell survival by functioning as a pro-apoptotic protein during DNA damage-induced apoptosis (via BCLAF1/Btf and p53), but acting as an anti-apoptotic protein during cytokine receptor-initiated cell death. Required for oxygen radical production by NADPH oxidase and acts as positive or negative regulator in platelet functional responses. Negatively regulates B cell proliferation and also has an important function in self-antigen induced B cell tolerance induction. In response to oxidative stress, activates CHUK/IKKA, causing the phosphorylation of p53/TP53. During ER stress or DNA damage-induced apoptosis, complexes with ABL1 to trigger apoptosis independently of p53/TP53. In the cytosol it triggers apoptosis by activating MAPK11 or MAPK14, inhibiting AKT1 and decreasing the level of XIAP, but in nucleus it induces apoptosis via the activation of MAPK8 or MAPK9. Upon ionizing radiation, required for the activation of BAX and BAK, which trigger the mitochondrial cell death pathway.	Stimulates phosphotransferase activity. S645 is phosphorylated by PKCd (PRKCD).
PKCd	S664	Protein-serine kinase C delta (PRKCD)	0.08	-0.08	-1.01	1.14	1.73	See above.	No data available.
PKCe	Pan-specific	Protein-serine kinase C epsilon (PRKCE)	0.13	0.27	-0.08	1.03	0.87	Ca-independent, phospholipid- and di-acylglycerol (DAG)-dependent serine/ threonine kinase that plays essential roles in the regulation of multiple cellular processes linked to cytoskeletal proteins, such as cell adhesion, motility, migration and cell cycle, functions in neuron growth and ion channel regulation, and is involved in immune response, cancer cell invasion and regulation of apoptosis. Mediates cell adhesion to the ECM via integrin-dependent signaling, by mediating angiotensin-2-induced activation of integrin beta-1 (ITGB1). Phosphorylates MARCKS, which phosphorylates and activates PTK2/FAK. Involved in control of the directional transport of ITGB1 in mesenchymal cells by phosphorylating vimentin. In epithelial cells, associates with and phosphorylates keratin-8 (KRT8), which induces targeting of desmoplakin at desmosomes and regulates cell-cell contact.	NA
PKCe	S729	Protein-serine kinase C epsilon (PRKCE)	0.51	0.79	1.39	0.98	0.98	See above.	Stimulates phosphotransferase activity. Phosphorylation regulates molecular association. S729 is phosphorylated by the following protein kinases in vitro: PKCe (PRKCE).

PKCg	T655	Protein-serine kinase C gamma (PRKCG)	0.72	-0.12	1.00	0.31	1.94	Calcium-activated, phospholipid- and diacylglycerol (DAG)-dependent serine/threonine kinase that plays diverse roles in neuronal cells and eye tissues, such as regulation of the neuronal receptors GRIA4/GLUR4 and GRIN1/NMDAR1, modulation of receptors and neuronal functions related to sensitivity to opiates, pain and alcohol, mediation of synaptic function and cell survival after ischemia, and inhibition of gap junction activity after oxidative stress.	Stimulates phosphotransferase activity. T655 is phosphorylated by the following protein kinases in vitro: PKCg (PRKCG).
PKCh	Pan-specific	Protein-serine kinase C eta (PRKCH)	-0.69	-0.74	-1.15	-0.41	-0.19	Calcium-independent, phospholipid- and DAG-dependent serine/threonine kinase involved in regulating cell differentiation in keratinocytes, pre-B cell receptor signaling, epithelial tight junction integrity and foam cell formation, actin depolymerization, and proliferation and apoptosis in some cell types. Activates FYN, which blocks EGFR signaling and leads to growth arrest and differentiation in some cell types. Associates with the cyclin CCNE1-CDK2-CDKN1B complex and inhibits CDK2 kinase activity, leading to RB1 dephosphorylation and G1 arrest. Regulates tight junctions (TJs) by phosphorylating occludin (OCLN), which is necessary for assembly and maintenance of TJs. In association with PLD2 and via TLR4 signaling, is involved in LPS-induced RGS2 down-regulation and foam cell formation. Can activate the mTOR pathway, the PI3K/AKT pathway and ERK1. Can regulate NF-kappa-B by activating IKBKB, and confer protection against DNA damage-induced apoptosis. Promotes oncogenic functions of ATF2 in the nucleus while blocking its apoptotic function at mitochondria.	NA
PKCl/I	Pan-specific	Protein-serine kinase C lambda/iota (PRKCI)	0.88	0.67	0.94	1.53	1.36	Calcium- and diacylglycerol-independent serine/ threonine kinase that plays a general protective role against apoptotic stimuli, is involved in NF-kappa-B activation, cell survival, differentiation and polarity, and contributes to the regulation of microtubule dynamics in the early secretory pathway. Is necessary for BCR-ABL oncogene-mediated resistance to apoptotic drugs in leukemia cells. In cultured neurons, prevents amyloid beta protein-induced apoptosis by interrupting cell death process at a very early step.	NA
PKCl/I	T564	Protein-serine kinase C lambda/iota (PRKCI)	0.25	1.86	4.08	3.48	4.88	See above.	No data available.

PKCm	Pan-specific	Protein-serine kinase C mu (Protein kinase D) (PRKD1, PKD1, PRKCM)	0.40	0.28	0.01	1.08	1.12	Serine/threonine kinase that converts transient DAG signals into prolonged physiological effects downstream of PKC, and is involved in the regulation of MAPK8/JNK1 and Ras signaling, Golgi membrane integrity and trafficking, cell survival through NF-kappa-B activation, cell migration, cell differentiation by mediating HDAC7 nuclear export, cell proliferation via ERK1/2 signaling, and plays a role in cardiac hypertrophy, VEGFA-induced angiogenesis, genotoxic-induced apoptosis and flagellin-stimulated inflammatory response. Phosphorylates EGFR, which leads to the suppression of EGF-induced MAPK8/JNK1 activation and subsequent JUN phosphorylation.	NA
PKCq	S695	Protein-serine kinase C theta (PRKCQ)	0.06	0.54	0.58	1.74	1.21	Calcium-independent, phospholipid- and DAG-dependent serine/threonine-protein kinase that mediates non-redundant functions in T-cell receptor (TCR) signaling, including T-cell activation, proliferation, differentiation and survival, by mediating activation of multiple transcription factors such as NF-kappa-B, JUN, NFATC1 and NFATC2. In TCR-CD3/CD28-co-stimulated T-cells, it is required for the activation of NF-kappa-B and JUN, which in turn are essential for IL2 production, and participates in the calcium-dependent NFATC1 and NFATC2 transactivation.	Stimulates phosphotransferase activity. Phosphorylation induces interaction with PDK1 and protein stabilization. S695 is phosphorylated by PKCt (PRKCQ).
PKCq	Pan-specific	Protein-serine kinase C theta (PRKCQ)	1.44	1.56	2.77	1.99	1.88	See above.	NA
PKR1	Pan-specific	Double stranded RNA dependent protein-serine kinase (PRKR; EIF2AK2)	0.45	0.78	0.40	0.05	1.15	IFN-induced dsRNA-dependent serine/threonine kinase which regulates the innate immune response to viral infection and is also involved in the regulation of signal transduction, apoptosis, cell proliferation and differentiation. Regulates replication via EIF2S1. Phosphorylates p53/TP53, PPP2R5A, DHX9, ILF3, IRS1 and the HHV-1 viral protein US11. Also has tyrosine-protein kinase activity and phosphorylates CDK1 upon DNA damage, facilitating its ubiquitination and proteosomal degradation. Either as an adapter protein and/or via its kinase activity, can regulate signaling through p38 MAP kinase, NF-kappa-B and insulin signaling pathways and transcription factors (JUN, STAT1, STAT3, IRF1, ATF3) regulating proinflammatory cytokines and IFNs.	NA
PKR1	T446	Double stranded RNA dependent protein-serine kinase (PRKR; EIF2AK2)	0.46	1.11	0.93	0.90	1.16	See above.	Stimulates phosphotransferase activity. Phosphorylation regulates cell cycle progression and cell growth. T446 is phosphorylated by PKR (PRKR; EIF2AK2).

PLCg1	Y771	1-phosphatidylinositol-4,5-bisphosphate phosphodiesterase gamma-1	-0.06	1.15	0.81	1.00	0.95	Mediates the production of the second messenger molecules diacylglycerol (DAG) and inositol 1,4,5-trisphosphate (IP3). Plays an important role in the regulation of intracellular signaling cascades. Becomes activated in response to ligand-mediated activation of receptor-type tyrosine kinases, such as PDGFRA, PDGFRB, FGFR1, FGFR2, FGFR3 and FGFR4. Plays a role in actin reorganization and cell migration.	Y771 is phosphorylated by EGFR, Syk.
PLCg2	Y753	1-phosphatidylinositol-4,5-bisphosphate phosphodiesterase gamma-2 (PLC R)	0.62	0.75	1.13	-0.66	0.98	The production of the second messenger molecules diacylglycerol (DAG) and inositol 1,4,5-trisphosphate (IP3) is mediated by activated phosphatidylinositol-specific phospholipase C enzymes. It is a crucial enzyme in transmembrane signaling.	Stimulates phospholipase activity, and regulates molecular association, including inducing interaction with Lyn. Y753 is phosphorylated by Btk, Fyn, Lck, Src.
PRKACA	Pan-specific	cAMP-dependent protein kinase catalytic subunit alpha	1.00	1.65	1.52	0.80	1.36	Phosphorylates a large number of substrates in the cytoplasm and the nucleus. Regulates the abundance of compartmentalized pools of its regulatory subunits through phosphorylation of PJA2 which binds and ubiquitinates these subunits, leading to proteolysis. Phosphorylates CDC25B, ABL1, NFKB1, CLDN3, PSMC5/RPT6, PJA2, RYR2, RORA and VASP. Involved in the regulation of platelets in response to thrombin and collagen; maintains circulating platelets in a resting state by phosphorylating proteins in numerous platelet inhibitory pathways when in complex with NF-kappa-B and I-kappa-B-alpha. Thrombin and collagen disrupt these complexes and free active PRKACA stimulates platelets and leads to platelet aggregation by phosphorylating VASP.	NA
PTEN	S380+T382+S385	Phosphatidylinositol-3,4,5-trisphosphate 3-phosphatase and protein phosphatase and tensin homolog deleted on chromosome 10	0.66	1.28	0.69	0.66	1.13	Acts as a dual-specificity protein phosphatase, dephosphorylating tyrosine-, serine- and threonine-phosphorylated proteins. Also acts as a lipid phosphatase, removing the phosphate in the D3 position of the inositol ring from phosphatidylinositols. Antagonizes the PI3K-AKT/PKB signaling pathway by dephosphorylating phosphoinositides and thereby modulating cell cycle progression and cell survival. The unphosphorylated form cooperates with AIP1 to suppress AKT1 activation. Key tumor suppressor.	Inhibits phosphatase activity, regulates cell differentiation, cell motility, transcription, and induces/inhibits interaction with PTEN. S380 is phosphorylated by CK2a1 (CSNK2A1), LKB1 (STK11), PKCz (PRKCZ). T382/T383 are phosphorylated by CK2a1 (CSNK2A1), LKB1 (STK11), MAGI2 (AIP1), PKCz (PRKCZ).
PTEN	S380+T382+T383	Phosphatidylinositol-3,4,5-trisphosphate 3-phosphatase and protein phosphatase and tensin homolog deleted on chromosome 10	0.60	0.71	1.03	0.58	0.98	See above.	See above.

Raf1 (c-Raf)	Pan-specific	Raf1 proto-oncogene-encoded protein-serine kinase (RafC)	0.13	0.65	0.76	0.76	1.43	Serine/threonine-protein kinase that acts as a regulatory link between the membrane-associated Ras GTPases and the MAPK/ERK cascade. Critical regulatory link functions as a switch determining cell fate decisions including proliferation, differentiation, apoptosis, survival and oncogenic transformation. RAF1 activation initiates a MAPK cascade via MEK1 and MEK2, leading to activation of ERK1/2. Phosphorylated RAF1 (Ser-338 and Ser-339, by PAK1) phosphorylates BAD/Bcl2-antagonist of cell death, adenylyl cyclases (ADCY2, ADCY5 and ADCY6) resulting in activation, PPP1R12A to inhibit phosphatase activity, and TNNT2/ cardiac muscle troponin T. Promotes NF-kappa-B activation and inhibits signal transducers involved in motility (ROCK2), apoptosis (MAP3K5/ASK1 and STK3/MST2), proliferation and angiogenesis (RB1). Can protect cells from apoptosis by translocating to the mitochondria to bind BCL2 and displace BAD/Bcl2-antagonist of cell death. Regulates Rho signaling and migration, and is required for normal wound healing. Represses the TJ protein, occludin (OCLN) via up-regulation of SNAI2/SLUG. Restricts caspase activation in response to selected stimuli, notably Fas stimulation, pathogen-mediated macrophage apoptosis, and erythroid differentiation.	NA
Rb	Pan-specific	Retinoblastoma-associated protein 1	0.18	1.06	0.76	0.47	0.84	Key regulator of entry into cell division; acts as a tumor suppressor. Promotes G0-G1 transition when phosphorylated by CDK3/cyclin-C. Acts as a transcription repressor of E2F1 target genes. The underphosphorylated, active form of RB1 interacts with E2F1 and represses its transcription activity, leading to cell cycle arrest. Directly involved in heterochromatin formation by maintaining overall chromatin structure and, in particular, that of constitutive heterochromatin by stabilizing histone methylation. Recruits and targets histone methyltransferases SUV39H1, KMT5B and KMT5C, leading to epigenetic transcriptional repression.	NA
Rb	S780	Retinoblastoma-associated protein 1	0.27	2.18	-0.04	0.30	0.76	See above.	Reported to both inhibit and induce interaction with E2F1. S780 is phosphorylated by the following protein kinases in vitro: CDK4, CDK6, PKACa (PRKACA).
Rb	S807	Retinoblastoma-associated protein 1	1.25	1.02	0.62	1.71	1.57	See above.	Inhibits interaction with Abl and EF21. S807 is phosphorylated by CDK1 (CDC2), CDK2, CDK3, CDK4, CDK6, CDK9.

RelB	Pan-specific	Transcription factor RelB	1.15	2.06	3.08	2.20	2.25	NF-kappa-B is a pleiotropic transcription factor which is present in almost all cell types and is involved in many biological processed such as inflammation, immunity, differentiation, cell growth, tumorigenesis and apoptosis. NF-kappa-B is a homo- or heterodimeric complex formed by the Rel-like domain-containing proteins RELA/p65, RELB, NFKB1/p105, NFKB1/p50, REL and NFKB2/p52.	NA
RelB	S573	Transcription factor RelB	0.49	1.35	2.58	1.26	1.45	See above.	No data available.
RIPK2	Y381	Receptor-interacting serine/threonine-protein kinase 2 (RIPK2, RIP2, RICK)	-0.54	-0.72	-1.07	-0.62	-0.62	Serine/threonine/tyrosine kinase that plays an essential role in modulation of innate and adaptive immune responses. Upon stimulation by bacterial peptidoglycans, NOD1 and NOD2 are activated, oligomerize and recruit RIPK2 through CARD-CARD domains. Contributes to the tyrosine phosphorylation of the guanine exchange factor ARHGEF2 through Src tyrosine kinase leading to NF-kappaB activation by NOD2. Once recruited, RIPK2 autophosphorylates and undergoes 'Lys-63'-linked polyubiquitination and mediates the recruitment of MAP3K7/TAK1 to IKBKG/NEMO. This ultimately leads to activation of IKBKB/IKKB and subsequent NF-kappa-B activation, which drives the transcription of genes involved in immune response, growth control, or protection against apoptosis. Also plays a role during engagement of the T-cell receptor (TCR).	No data available.
ROCK2	Pan-specific	Rho-associated protein kinase 2 (ROKa)	0.32	1.62	2.41	0.83	1.80	Protein kinase which is a key regulator of actin cytoskeleton and cell polarity. Involved in regulation of smooth muscle contraction, actin cytoskeleton organization, stress fiber and focal adhesion formation, neurite retraction, cell adhesion and motility via phosphorylation of ADD1, BRCA2, CNN1, EZR, DPYSL2, EP300, MSN, MYL9/MLC2, NPM1, RDX, PPP1R12A and VIM. Phosphorylates SORL1 and IRF4. Acts as a negative regulator of VEGF-induced angiogenic endothelial cell activation. Positively regulates the activation of p42/MAPK1-p44/MAPK3 and of p90RSK/RPS6KA1 during myogenic differentiation. Plays an important role in the timely initiation of centrosome duplication.	NA

Ron	Pan-specific	Macrophage-stimulating protein receptor alpha chain (RONa)	0.10	0.31	1.46	0.47	1.40	RTK that transduces signals from the ECM into the cytoplasm by binding to MST1 ligand or GFs. Regulates many physiological processes including cell survival, migration and differentiation. Ligand binding at the cell surface induces autophosphorylation of RON on its intracellular domain, providing docking sites for downstream signaling molecules. This leads to interaction with PI3K subunit PIK3R1, PLCG1 or the adapter GAB1 and subsequent activation of several signaling cascades including the RAS-ERK, PI3K-AKT, or PLCgamma-PKC. RON signaling activates the wound healing response, proliferation as well as survival at the wound site. Also plays a role in the innate immune response by regulating the migration and phagocytic activity of macrophages.	NA
RPS6	S235	40S ribosomal protein S6	0.51	0.52	2.64	1.43	2.25	May play an important role in controlling cell growth and proliferation through the selective translation of particular classes of mRNA.	Phosphorylation at S235 and S236 facilitates the assembly of the preinitiation complex. S235 is phosphorylated by Akt1 (PKBa), Akt2 (PKBb), AurB (STK12), p70S6K (RPS6KB1), PKACa (PRKACA), PKCd (PRKCD), RSK1 (RPS6KA2), RSK2 (RPS6KA3).
RSK1	T359	Ribosomal S6 protein-serine kinase 1 (RPS6KA1, p90RSK)	-0.20	-0.12	0.31	1.67	0.62	Serine/threonine kinase that acts downstream of ERK signaling and positively regulates mitogenic and stress-induced activation of CREB1, CREBBP, ETV1/ER81 and NR4A1/NUR77, positively regulates translation through RPS6 and EIF4B phosphorylation, and mediates cellular proliferation, survival, and differentiation by modulating mTOR signaling and repressing pro-apoptotic function of BAD and DAPK1. Upon insulin-derived signal, phosphorylates GSK3B to inhibit its activity and EIF4B to enhance EIF4B affinity for the EIF3 complex to stimulating cap-dependent translation. Involved in the mTOR nutrient-sensing pathway by phosphorylating TSC2 to prevent its suppression of mTOR signaling, and via phosphorylation of RPTOR (regulates mTORC1 activity). Involved in cell cycle regulation by phosphorylating the CDK inhibitor CDKN1B. Phosphorylates EPHA2 to regulate cell migration.	Stimulates phosphotransferase activity. T359 is phosphorylated by ERK1 (MAPK3), ERK2 (MAPK1).
RSK1	T573	Ribosomal S6 protein-serine kinase 1 (RPS6KA1, p90RSK)	0.70	-0.24	-0.92	-1.10	-0.52	See above.	Stimulates phosphotransferase activity. T573 is phosphorylated by: ERK1 (MAPK3), ERK2 (MAPK1).

SCYL1	S754	N-terminal kinase-like protein	-0.22	0.69	0.41	1.36	0.91	Regulates COPI-mediated retrograde protein traffic at the interface between the Golgi apparatus and the endoplasmic reticulum.	No data available.
Sgk223	Y413	Tyrosine-protein kinase SgK223	-0.65	-0.78	-1.01	-0.29	-0.61	Tyrosine protein kinase family; a similar protein in rat binds to Rho family GTPases and regulates neurite outgrowth via activation of RhoA.	No data available.
SIK3 (QSK)	Pan-specific	Serine/threonine-protein kinase SIK3	0.74	-1.01	-0.78	-0.82	-0.42	Positive regulator of mTOR signaling that functions by triggering the degradation of DEPTOR, an mTOR inhibitor. Involved in the dynamic regulation of mTOR signaling in chondrocyte differentiation during skeletogenesis; negatively regulates cAMP signaling pathway possibly by acting on CRTC2/TORC2 and CRTC3/TORC3; prevents HDAC4 translocation to the nucleus.	NA
SMC1	S957	Structural maintenance of chromosomes protein 1A	1.42	0.95	1.26	2.07	1.63	Involved in chromosome cohesion during cell cycle and in DNA repair. Central component of cohesin complex. The cohesin complex is required for the cohesion of sister chromatids after DNA replication. The cohesin complex may also play a role in spindle pole assembly during mitosis. Involved in DNA repair via its interaction with BRCA1 and its related phosphorylation by ATM, or via its phosphorylation by ATR. Works as a downstream effector both in the ATM/NBS1 branch and in the ATR/MSH2 branch of S-phase checkpoint.	S957 is phosphorylated by ATM, ATR.
snRNP 70	Y126	U1 small nuclear ribonucleoprotein 70 kDa	-0.29	-0.94	-0.62	-0.31	-0.99	Component of the spliceosomal U1 snRNP, which is essential for recognition of the pre-mRNA 5' splice-site and the subsequent assembly of the spliceosome. SNRNP70 binds to the loop I region of U1-snRNA. The truncated isoforms cannot bind U1-snRNA.	No data available.
SRC	Pan-specific	Src proto-oncogene-encoded protein-tyrosine kinase	0.62	2.04	0.19	2.97	1.73	Non-receptor protein tyrosine kinase which is activated following engagement of many different classes of cellular receptors including immune response receptors, integrins and other adhesion receptors, receptor protein tyrosine kinases, G protein-coupled receptors as well as cytokine receptors. Participates in signaling pathways that control a diverse spectrum of biological activities including gene transcription, immune response, cell adhesion, cell cycle progression, apoptosis, migration, and transformation. Receptor clustering or dimerization leads to recruitment of SRC to the receptor complexes where it phosphorylates the tyrosine residues within the receptor cytoplasmic domains.	NA

STAT1	Y701	Signal transducer and activator of transcription 1 beta	0.91	7.41	4.86	7.52	3.16	Signal transducer and transcription activator that mediates cellular responses to interferons (IFNs), cytokine KITLG/SCF and other cytokines and other growth factors. Following type I IFN (IFN-alpha and IFN-beta) binding to cell surface receptors, signaling via protein kinases leads to activation of Jak kinases (TYK2 and JAK1) and to tyrosine phosphorylation of STAT1 and STAT2. In response to type II IFN (IFN-gamma), STAT1 is tyrosine- and serine-phosphorylated. It then forms a homodimer termed IFN-gamma-activated factor (GAF), migrates into the nucleus and binds to the IFN gamma activated sequence (GAS) to drive the expression of the target genes. Activation of the JAK-STAT pathway drives the cell into an antiviral state. May mediate cellular responses to activated FGFR1, FGFR2, FGFR3 and FGFR4.	S222 is phosphorylated by CK2a1 (CSNK2A1), CK2a2 (CSNK2A2).
STAT2	Pan-specific	Signal transducer and activator of transcription 2	0.63	1.15	0.42	3.61	1.63	Signal transducer and activator of transcription that mediates signaling by type I IFNs. Following IFN binding to cell surface receptors, Jak kinases (TYK2 and JAK1) are activated, leading to phosphorylation of STAT1/2. The phosphorylated STATs dimerize, and associate with IRF9/ISGF3G to form a complex that enters the nucleus and drives antiviral responses. Acts as a regulator of mitochondrial fission by modulating the phosphorylation of DNM1L.	NA
STAT2	Y690	Signal transducer and activator of transcription 2	-0.78	2.20	1.00	0.14	1.07	See above.	Stimulates transcriptional activity. Y690 is phosphorylated by JAK1, Lck, Tyk2.
STAT3	Pan-specific	Signal transducer and activator of transcription 3 (acute phase response factor)	0.47	3.66	4.05	3.83	2.63	Signal transducer and transcription activator that mediates cellular responses to interleukins, KITLG/SCF, LEP and other growth factors. Involved in cell cycle regulation by inducing expression of key genes for progression from G1 to S phase, such as CCND1. Mediates the effects of LEP on melanocortin production, body energy homeostasis and lactation. May play an apoptotic role by transctivating BIRC5 expression under LEP activation. Cytoplasmic STAT3 represses macroautophagy by inhibiting EIF2AK2/PKR activity.	NA
STAT5A	Pan-specific	Signal transducer and activator of transcription 5A	0.75	2.15	1.94	4.81	3.03	Carries out a dual function: signal transduction and activation of transcription. Mediates cellular responses to the cytokine KITLG/SCF and other growth factors. Mediates cellular responses to ERBB4. May mediate cellular responses to activated FGFR1, FGFR2, FGFR3 and FGFR4. Binds to the GAS element and activates PRL-induced transcription. Regulates the expression of milk proteins during lactation.	NA

STAT5A	S780	Signal transducer and activator of transcription 5A	0.46	1.61	1.09	2.66	1.11	See above.	Regulates molecular association, including inhibiting interaction with ERK1. S780 is phosphorylated by ERK1 (MAPK3), ERK2 (MAPK1), PAK1.
STAT5B	Pan-specific	Signal transducer and activator of transcription 5B	0.51	1.67	1.46	4.66	3.68	Carries out a dual function: signal transduction and activation of transcription. Mediates cellular responses to the cytokine KITLG/SCF and other growth factors. Mediates cellular responses to ERBB4. May mediate cellular responses to activated FGFR1, FGFR2, FGFR3 and FGFR4. Binds to the GAS element and activates PRL-induced transcription. Regulates the expression of milk proteins during lactation.	NA
SYK	Pan-specific	Spleen protein-tyrosine kinase	0.91	1.90	1.27	0.76	0.46	Non-receptor tyrosine kinase which mediates signal transduction downstream of a variety of transmembrane receptors such as the B-cell receptor (BCR). Regulates several biological processes including innate and adaptive immunity, cell adhesion, osteoclast maturation, platelet activation and vascular development. Assembles into signaling complexes with activated receptors at the plasma membrane via interaction between its SH2 domains and the receptor tyrosine-phosphorylated ITAM domains.	NA
SYK	Y323	Spleen protein-tyrosine kinase	0.29	0.51	0.07	1.07	0.39	See above.	Stimulates phosphotransferase activity and regulates molecular association, including inducing interaction with Cbl, and Fyn. Y323 is phosphorylated by Lck, Lyn, Syk.
SYK	Y323	Spleen protein-tyrosine kinase	0.14	0.85	0.75	1.45	0.74	See above.	Stimulates phosphotransferase activity and regulates molecular association, including inducing interaction with Cbl, and Fyn. Y323 is phosphorylated by Lck, Lyn, Syk.
SYK	Y352	Spleen protein-tyrosine kinase	-0.70	0.06	0.15	4.06	0.13	See above.	Stimulates phosphotransferase activity and regulates molecular association, including inducing interaction with ITGAM, PLCG1, and CLTC. Y352 is phosphorylated by the following protein kinases in vitro: Lck, Lyn, Syk.

Tau	T522	Microtubule-associated protein tau	1.03	1.43	1.05	2.45	2.05	Promotes microtubule assembly and stability. The C-terminus binds axonal microtubules while the N-terminus binds neural plasma membrane components, suggesting that tau functions as a linker protein between both. The short isoforms allow plasticity of the cytoskeleton whereas the longer isoforms may preferentially play a role in its stabilization.	Phosphorylation disrupts tau binding to microtubules. T522 is phosphorylated by GSK3-beta, CDK5, CK1-delta.
TBK1	Pan-specific	Serine/threonine-protein kinase TBK1	0.11	1.05	0.20	0.73	-0.02	Serine/threonine kinase that plays an essential role in regulating inflammatory responses to foreign agents. Following activation of TLRs by viral or bacterial components, associates with TRAF3 and TANK and phosphorylates interferon regulatory factors (IRFs) IRF3 and IRF7 and DDX3X to promote inflammatory responses. Several scaffolding molecules including FADD, TRADD, MAVS, AZI2, TANK or TBKBP1/SINTBAD can be recruited to the TBK1-containing-complexes. Can induce NF-kappa-B and AKT1 activation. Restricts bacterial proliferation by phosphorylating the autophagy receptor OPTN/Optineurin on 'Ser-177', thus enhancing LC3 binding affinity and antibacterial autophagy. Phosphorylates SMCR8 of the C9orf72-SMCR8 complex, promoting autophagosome maturation.	NA
TEC	Y519	Tyrosine-protein kinase Tec	1.02	1.90	1.70	3.30	1.84	Non-receptor tyrosine kinase that contributes to signaling from many receptors and participates as a signal transducer in multiple downstream pathways, including regulation of the actin cytoskeleton. Redundant role to ITK in regulation of the adaptive immune response. Regulates development, function and differentiation of conventional T-cells and nonconventional NKT-cells. Required for TCR-dependent IL2 gene induction. Phosphorylates DOK1 to contribute to CD28-signaling. Negatively regulates IL2RA expression induced by TCR cross-linking. Redundant role to BTK in BCR-signaling for B-cell development and activation. Required in mast cells for efficient cytokine production. Promotes growth, differentiation and activation of myeloid cells through activation by CSF3 (a critical cytokine for myeloid cells). Participates in platelet signaling downstream of integrin activation. Cooperates with JAK2 to mediate cytokine-driven activation of FOS. Involved in GPCR and integrin-mediated signaling in blood platelets, in hepatocyte proliferation and liver regeneration, and in HGF-induced ERK signaling pathway. Regulates FGF2 unconventional secretion (endoplasmic reticulum (ER)/Golgi-independent mechanism) through phosphorylation of FGF2 'Tyr-215'. May also be involved in the regulation of osteoclast differentiation.	Stimulates phosphotransferase activity.

TGM2	Y369	Protein-glutamine gamma-glutamyltransferase 2	0.06	0.15	-0.17	1.24	0.05	Catalyzes the cross-linking of proteins and the conjugation of polyamines to proteins.	No data available.
TRKB	Y706	BNDF/NT3/4/5 receptor-tyrosine kinase (NTRK2)	0.07	0.53	0.05	1.62	1.15	Receptor tyrosine kinase involved in the development and the maturation of the central and the peripheral nervous systems through regulation of neuron survival, proliferation, migration, differentiation, and synapse formation and plasticity.	Stimulates phosphotransferase activity. Y706 is phosphorylated by TrkB (NTRK2).
TTK	Pan-specific	Dual specificity protein kinase	0.22	0.42	0.42	1.04	1.19	Phosphorylates proteins on serine, threonine, and tyrosine. Probably associated with cell proliferation. Essential for chromosome alignment by enhancing AURKB activity (via direct CDCA8 phosphorylation) at the centromere, and for the mitotic checkpoint.	NA
TYK2	Pan-specific	Protein-tyrosine kinase 2 (Jak-related)	0.06	1.54	0.24	1.03	1.44	Involved in intracellular signal transduction by being involved in the initiation of type I IFN signaling. Phosphorylates the interferon-alpha/beta receptor alpha chain.	NA
TYRO3	Y681	Tyrosine-protein kinase receptor TYRO3	-1.06	-0.34	-0.31	-0.09	-0.28	Receptor tyrosine kinase that transduces signals from the extracellular matrix into the cytoplasm by binding to several ligands including TULP1 or GAS6. Regulates many physiological processes including cell survival, migration and differentiation. Ligand binding induces dimerization and autophosphorylation of TYRO3 on its intracellular domain that provides docking sites for downstream signaling molecules. Following activation by ligand, interacts with PIK3R1 and thereby enhances PI3-kinase activity. Activates the AKT survival pathway and subsequently upregulates NF-kappa-B-regulated genes. TYRO3 signaling plays a role in various processes such as neuron protection from excitotoxic injury, platelet aggregation and cytoskeleton reorganization. Also inhibits Toll-like receptor (TLR)-mediated innate immune response by activating STAT1, which selectively induces production of suppressors of cytokine signaling SOCS1 and SOCS3.	Predicted to be stimulatory for phosphotransferase activity.
VEGFR3 (FLT4)	Pan-specific	Vascular endothelial growth factor receptor-protein-tyrosine kinase 3 (VEGFR3)	-1.00	-0.72	-0.92	-0.36	-0.26	Tyrosine-protein kinase that acts as a cell-surface receptor for VEGFC and VEGFD, and plays an essential role in adult lymphangiogenesis and in the development of the vascular network and the cardiovascular system during embryonic development. Promotes proliferation, survival and migration of endothelial cells, and regulates angiogenic sprouting. Signaling by activated FLT4 leads to enhanced production of VEGFC, and to a lesser degree VEGFA, thereby creating a positive feedback loop that enhances FLT4 signaling. Modulates KDR signaling by forming heterodimers.	NA

Vimentin	S34	VIM (Vimentin)	0.53	1.46	1.82	1.26	1.49	Vimentins are class-III intermediate filaments found in various non-epithelial cells, especially mesenchymal cells. Vimentin is attached to the nucleus, endoplasmic reticulum, and mitochondria, either laterally or terminally.	S34 is phosphorylated by PKCa (PRKCA).
WNK1	T60	Serine/threonine-protein kinase WNK1 (PRKWNK1)	0.33	0.81	0.80	1.06	1.50	Serine/threonine kinase; regulates electrolyte homeostasis, cell signaling, survival, and proliferation. Acts as an activator and inhibitor of sodium-coupled chloride cotransporters and potassium-coupled chloride cotransporters respectively. Activates SCNN1A, SCNN1B, SCNN1D and SGK1. Controls sodium and chloride ion transport by inhibiting the activity of WNK4. WNK4 regulates activity of the thiazide-sensitive Na-Cl cotransporter, SLC12A3, by phosphorylation. WNK1 may regulate actin cytoskeletal reorganization.	Stimulates phosphotransferase activity. T60 is phosphorylated by Akt1 (PKBa), Akt3 (PKBg), MSK1 (RPS6KA5), SGK1.
ZAP70	Pan-specific	Zeta-chain (TCR) associated protein-tyrosine kinase, 70 kDa	-0.38	0.20	0.13	-0.04	1.29	Tyrosine kinase that plays an essential role in regulation of the adaptive immune response. Regulates motility, adhesion and cytokine expression of mature T-cells, as well as thymocyte development. Contributes also to the development and activation of primary B-lymphocytes. Release of ZAP70 active conformation is stabilized by phosphorylation mediated by LCK. ZAP70 phosphorylates at least 2 essential adapter proteins: LAT and LCP2. In turn, a large number of signaling molecules are recruited and ultimately lead to lymphokine production, T-cell proliferation and differentiation. Furthermore, ZAP70 controls cytoskeleton modifications, adhesion and mobility of T-lymphocytes, thus ensuring correct delivery of effectors to the APC.	NA
